# VizBin - an application for reference-independent visualization and human-augmented binning of metagenomic data

**DOI:** 10.1186/s40168-014-0066-1

**Published:** 2015-01-20

**Authors:** Cedric C Laczny, Tomasz Sternal, Valentin Plugaru, Piotr Gawron, Arash Atashpendar, Houry Hera Margossian, Sergio Coronado, Laurens van der Maaten, Nikos Vlassis, Paul Wilmes

**Affiliations:** Luxembourg Centre for Systems Biomedicine, University of Luxembourg, Esch-sur-Alzette, 4362 Luxembourg; Institute of Computing Science, Poznan University of Technology, Poznan, 60-965 Poland; Computer Science and Communications Research Unit, University of Luxembourg, Luxembourg, 1359 Luxembourg; Pattern Recognition and Bioinformatics Group, Delft University of Technology, CD Delft, 2628 Netherlands; Adobe Research, Adobe, San Jose, 95110 USA

**Keywords:** Metagenomics, Machine learning, Visualization, Binning

## Abstract

**Background:**

Metagenomics is limited in its ability to link distinct microbial populations to genetic potential due to a current lack of representative isolate genome sequences. Reference-independent approaches, which exploit for example inherent genomic signatures for the clustering of metagenomic fragments (binning), offer the prospect to resolve and reconstruct population-level genomic complements without the need for prior knowledge.

**Results:**

We present VizBin, a Java™-based application which offers efficient and intuitive reference-independent visualization of metagenomic datasets from single samples for subsequent human-in-the-loop inspection and binning. The method is based on nonlinear dimension reduction of genomic signatures and exploits the superior pattern recognition capabilities of the human eye-brain system for cluster identification and delineation. We demonstrate the general applicability of VizBin for the analysis of metagenomic sequence data by presenting results from two cellulolytic microbial communities and one human-borne microbial consortium. The superior performance of our application compared to other analogous metagenomic visualization and binning methods is also presented.

**Conclusions:**

VizBin can be applied *de novo* for the visualization and subsequent binning of metagenomic datasets from single samples, and it can be used for the *post hoc* inspection and refinement of automatically generated bins. Due to its computational efficiency, it can be run on common desktop machines and enables the analysis of complex metagenomic datasets in a matter of minutes. The software implementation is available at https://claczny.github.io/VizBin under the BSD License (four-clause) and runs under Microsoft Windows™, Apple Mac OS X™ (10.7 to 10.10), and Linux.

**Electronic supplementary material:**

The online version of this article (doi:10.1186/s40168-014-0066-1) contains supplementary material, which is available to authorized users.

## Background

Mixed microbial communities are ubiquitous and play fundamental roles in the Earth’s biogeochemical cycles, as well as in human health. Shotgun sequencing of extracted DNA from microbial consortia allows for culture-independent analysis of their composition and/or genetic potential. So far, metagenomics has been applied to a panoply of microbial communities of differing complexities [1-6].

In metagenomic analyses, the characterization of constituent populations is typically carried out using reference-based approaches whereby sequence fragments, e.g., filtered sequence reads or reconstructed genomic fragments (contigs), are aligned to previously characterized isolate genomes [7,8]. However, disparities between the genomes of isolate strains and natural populations [9] as well as the lack of a comprehensive set of representative reference genomes [7] results in the need for reference-independent analysis approaches.

Reference-independent deconvolution of metagenomic datasets from single samples generally relies on the use of data-inherent characteristics, e.g., oligonucleotide composition [10], to group metagenomic fragments into clusters (bins) comprising sequence fragments derived from distinct microbial populations. To determine oligonucleotide sequence composition, distinct *k*mers are counted over sequence fragments and counts are normalized to represent frequency distributions [11] resulting in vectors (genomic signatures) of fixed dimensionality, 4^*k*^. So far, the exploration of the signature space has been hampered by the comparably high dimensionality of genomic signatures: for *k*=5, the vectors are embedded in a 1,024-dimensional space. To reduce the dimensionality of the data, approaches based on self-organizing maps (SOMs) have been used for the visualization and delineation of population-specific sequence clusters, e.g., emergent SOMs (ESOMs) [12,13].

We have recently demonstrated [14] that nonlinear dimension reduction of centered log-ratio transformed genomic signatures via Barnes-Hut stochastic neighbor embedding (BH-SNE; [15]) results in improved performance in terms of decreased input sequence lengths, decreased computation time, increased homogeneity of clusters, and more intuitive interpretation compared to the more traditional ESOM-based approaches. Here, we present VizBin, a cross-platform software implementation of the method for the rapid and reliable reference-independent visualization and subsequent human-augmented binning of metagenomic datasets from single samples based on a parallelized version of BH-SNE (https://claczny.github.io/VizBin).

## Implementation

VizBin is a graphical user interface (GUI)-based desktop application for Microsoft Windows™, Apple Mac OS X™ (10.7 to 10.10), and Linux. The GUI is written in Java™ and makes use of different Java™ libraries as described in https://github.com/claczny/VizBin. The only runtime requirements of VizBin are a working Java™ installation and the Java™ Standard Edition Runtime Environment (JRE; http://www.oracle.com/technetwork/java/javase/index.html). This work implements parallelization into the original C source code for BH-SNE (http://homepage.tudelft.nl/19j49/t-SNE.html) by integrating the OpenMP®; Application Programming Interface (API). VizBin incorporates this parallelized version of BH-SNE for the computation of the two-dimensional embedding which is then visualized by the GUI. A copy of the VizBin software can be downloaded from http://claczny.github.io/VizBin.

### General description

**Figure 1:**
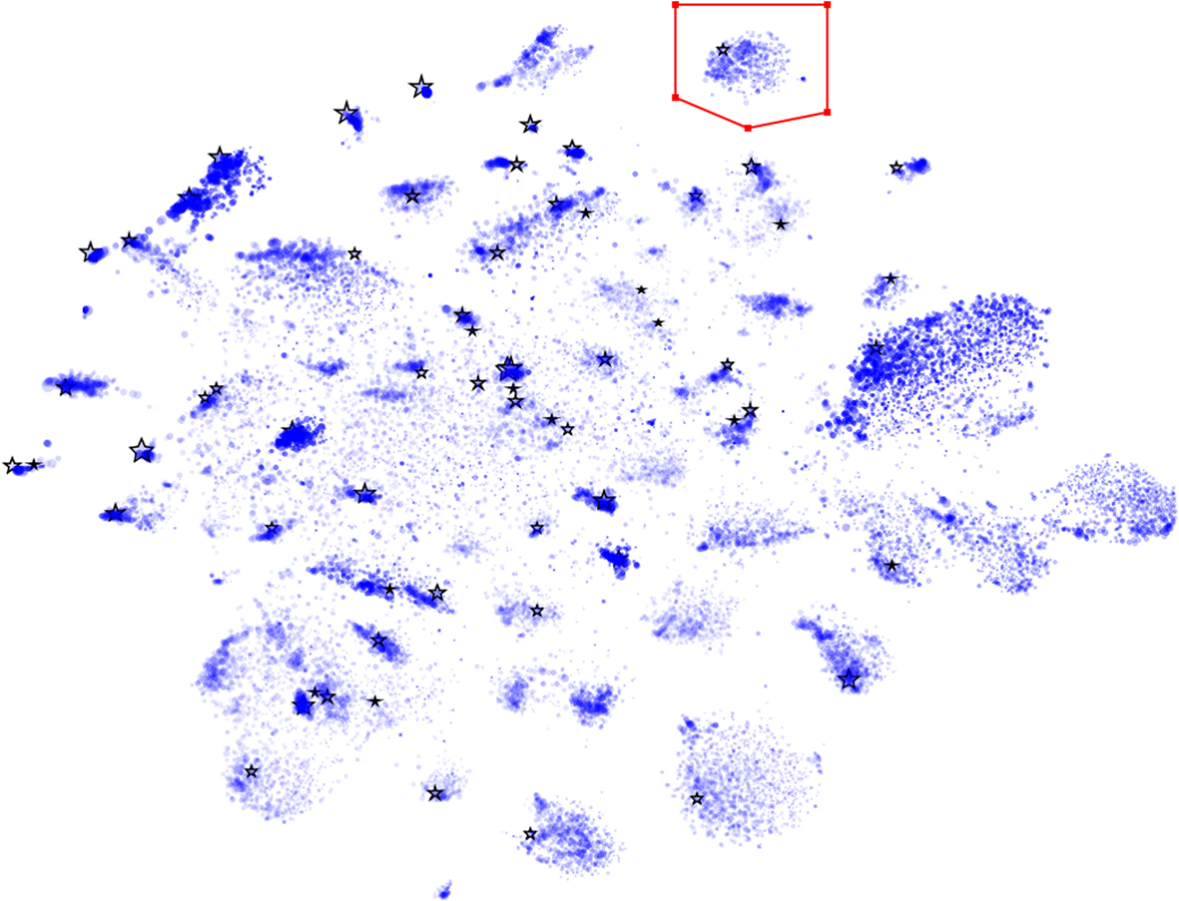
**Visualization and polygonal selection in VizBin.** Scatter plot visualization in VizBin of a groundwater-derived metagenomic dataset [20]. The manually placed red polygon highlights a selected cluster of interest. The corresponding sequences can be exported for further analysis. Minimal fragment length: 1,000 nt. Point size is proportional to the natural logarithm of sequence fragment length. Opaqueness is proportional to the natural logarithm of coverage (coverage values according to alignment of reads from [20] to the contigs). A star-like shape highlights contigs annotated to contain the GrpE gene.

A key feature of VizBin is that it allows the visualization and subsequent binning of metagenomic fragments, e.g., contigs or long reads, for users without any bioinformatic background and exploits the superior pattern recognition capabilities of the human eye-brain system for cluster identification and delineation. The VizBin application presents the user with a simple dialogue to specify the input FASTA-file containing the metagenomic fragments of interest. The user can then choose a minimum input sequence length before the calculation of the genomic signatures. By default, the program will only consider fragments of at least 1,000 nt in length, but lower thresholds can also be defined. However, depending on the dataset, clusters are expected to overlap when the lengths approach 600 nt [14]. Due to VizBin’s fast processing speed, different length thresholds can be applied and tailored thresholds can be identified iteratively. Additional per-sequence information can be provided by the user in the form of an annotation file. This consists of a comma-separated file including information on sequence length, coverage, %GC, a label (e.g., taxonomic ID), and whether a sequence contains a marker gene of interest or not. Following the definition of parameters and genomic signature calculation, BH-SNE [15] is performed on the centered log ratio-transformed signatures, resulting in a two-dimensional scatter plot. If provided by the user, sequence length can be flexibly represented as the size of a point, coverage or %GC as opaqueness, and label as an individual color and shape of the point and a sequence which contains a marker gene of interest can be represented by a star shape to highlight the corresponding point in the scatter plot (Figure 1). The user can navigate (pan and zoom) to inspect the plot as well as use the polygonal selection tool for the definition of clusters of interest (Figure 1). The corresponding sequences can subsequently be exported in FASTA format for further downstream analyses [14]. More detailed documentation, including tutorials on saving/loading two-dimensional embeddings obtained by VizBin, example files, etc., can be found at the project’s wiki (https://github.com/claczny/VizBin/wiki).

## Results and discussion

In this section, we compare the performance of our approach to a commonly used method for visualizing metagenomic data, i.e., ESOM-based visualization, as well as to a state-of-the-art fully automated binning method (MaxBin; [16]). The performances of VizBin and MaxBin were quantitatively assessed by inferring the homogeneity and completeness of bins using a collection of 107 single-copy marker genes. These genes, referred to in the following as ‘essential genes’, are conserved in 95% of all sequenced bacteria [17] and have been previously used to assess the performance of different binning methods [18]. When using this essential gene set, increased homogeneity (lower amount of multiple essential gene copies) and increased completeness (higher fraction of essential genes recovered) indicate better binning performance.

### Comparison against the ESOM-based approach

**Figure 2:**
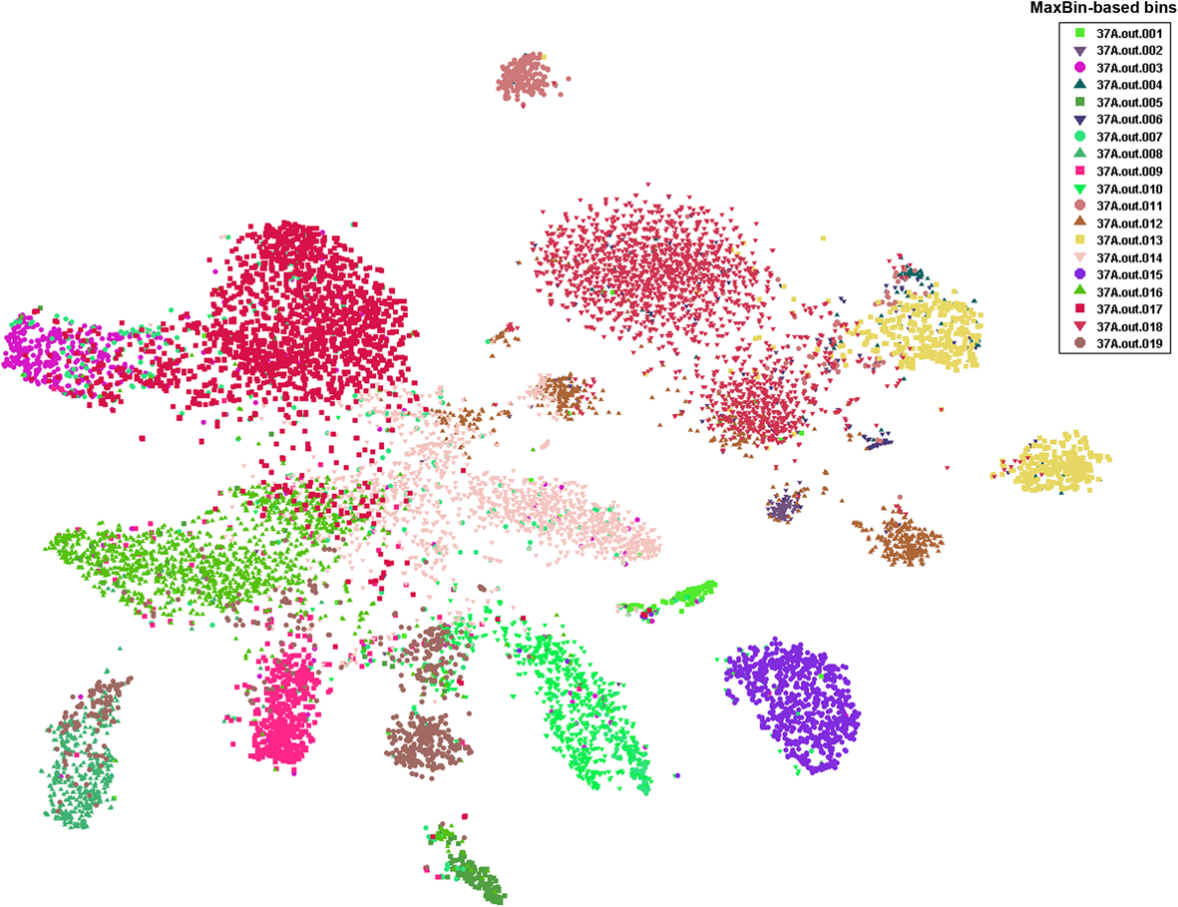
**Comparison of sequence clusters apparent in VizBin to bins previously defined using MaxBin [** 16 **].** Scatter plot visualization of a cellulolytic microbial community metagenomic dataset (37A) [16]. Minimal fragment length: 1,000 nt. Points coloured according to original MaxBin-based bins.

To demonstrate the effectiveness of VizBin, we ran it on a previously described cellulolytic microbial community dataset (37A) which was recently used to asses the performance of the MaxBin approach [16]. Individual clusters are apparent in VizBin which are largely in accordance with the original MaxBin-based assignments (Figure 2). The ESOM-based results (Additional file 1: Figure S1A), however, only show a small number of apparent clusters. This visual appearance is supported by the results of applying the flood-fill algorithm with the lowest threshold (0.1; Additional file 1: Figure S1B). The ESOM-based clusters only become clearly visible when layering the clustering of contigs using MaxBin on top of the topological map (Additional file 1: Figure S1C). These results demonstrate the superior performance of VizBin compared to the ESOM-based approach for the *de novo* reference-independent visualization and subsequent human-augmented binning of metagenomic data. Despite a general accordance between VizBin and MaxBin, clusters are visible that share the same color but clearly are separate in the VizBin plot (Figure 2).

### Comparison against MaxBin

MaxBin [16] is a state-of-the-art fully automated reference-independent binning approach that uses coverage information in addition to oligonucleotide frequencies. Coverage information is obtained by mapping the sequencing reads back onto the assembled contigs. Clusters are identified automatically via expectation maximization.

We used VizBin to inspect the original MaxBin-based bins of two cellulolytic microbial communities (37A and 37B) [16] and one human-borne microbial consortium (SRS013705; tongue dorsum) [19]. For individual bin visualizations, see Additional file 2. The MaxBin-based bins are often comprised of single clusters in the VizBin plots (Figure 2; Additional file 1: Figure S2). However, numerous exceptions exist where multiple subclusters are apparent for single MaxBin-based bins (Figure 2; Additional file 1: Figure S3). Given the previously demonstrated ability of our approach for resolving population-level genomic complements [14x], these MaxBin-based bins likely represent mixtures of sequences derived from originally distinct microbial populations, thus suggesting heterogeneity in the automatically generated bins. This suggestion is supported by the occurrence of essential genes in multiple copies in the original MaxBin-based bins (Additional file 1: Tables S1-S3).

**Figure 3:**
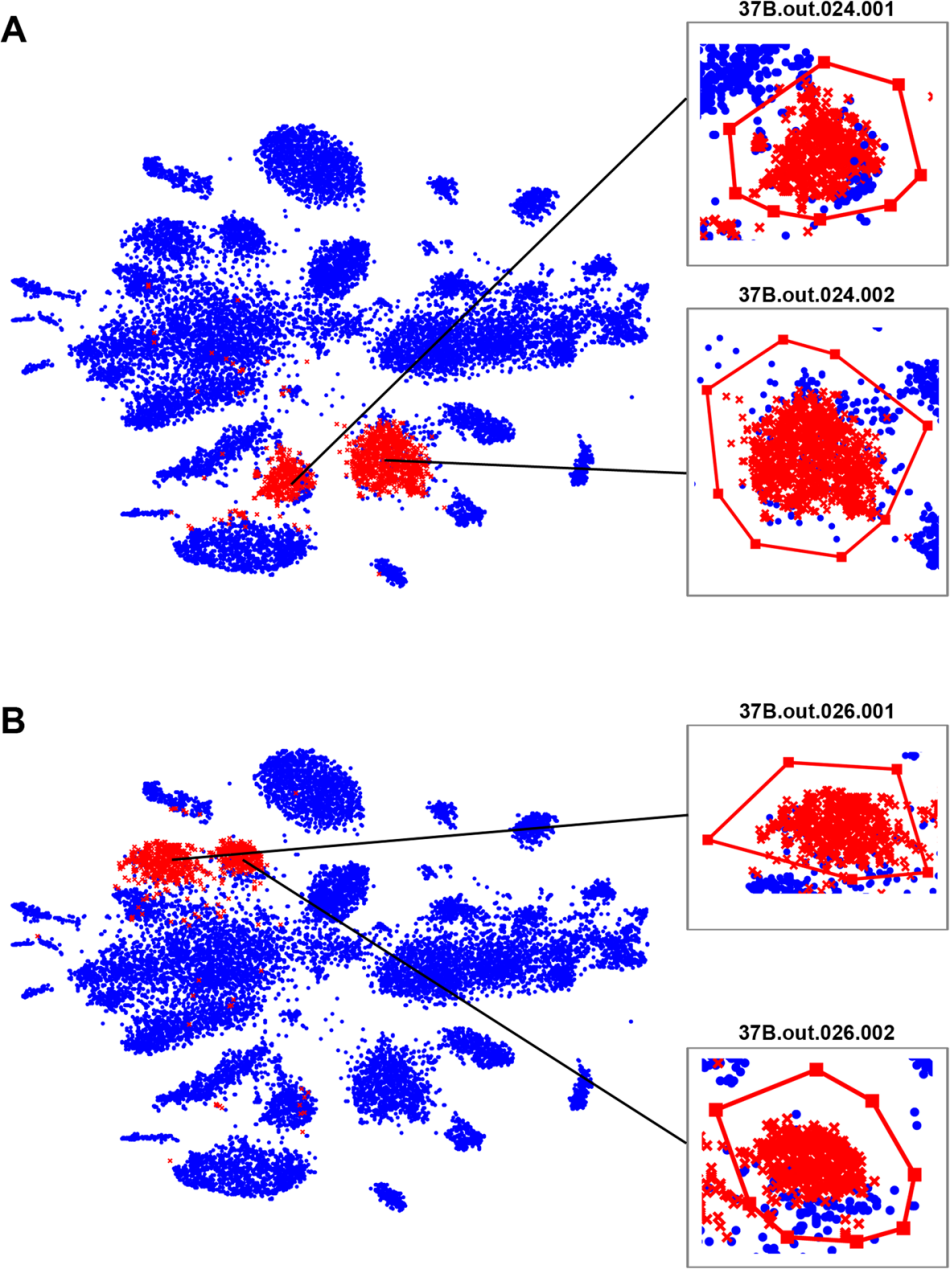
**Visualization and polygonal selection of clusters from a cellulolytic microbial consortium metagenomic dataset 37B [** 16 **].** Points highlighted in red according to contig assignment in MaxBin: **(A)** bin 37B.out.024 and **(B)** bin 37B.out.026, respectively. Individual subclusters (37B.out.024.001, 37B.out.024.002, 37B.out.026.001, and 37B.out.026.002) are highlighted with inserts showing closeups. Minimal fragment length: 1,000 nt.

Using the example of the 37B dataset, bins 37B.out.024 and 37B.out.026 exhibit each two pronounced and wellseparated subclusters (Additional file 1: Figure S3A,B). This suggests that these bins should each be subdivided. Additional prominent examples from the other datasets include 37A.out.014, 37A.out.018, SRS013705.out.004, SRS013705.out.026, and SRS013705.out.029 (Additional file 1: Figure S3C-G). Using all originally binned contigs from 37B (17,622 in total), we coloured the contigs originally assigned to bins 37B.out.24 and 37B.out.26, respectively (Figure 3). We then applied the polygonal selection tool in VizBin to delineate and export the sequences for each apparent subcluster (per individual MaxBin-based bin) for further inspection of their homogeneity and completeness. The resulting subclusters in 37B.out.024 (defined herein as 37B.out.024.001, 37B.out.024.002; Figure 3A) and 37B.out.026 (37B.out.026.001, 37B.out.026.002; Figure 3B) exhibit increased homogeneity as well as similar or increased completeness (Table 1). The increased homogeneity results from the separation of originally mixed metagenomic fragments. The increased completeness, in turn, is due to the recruitment of new metagenomic fragments (as compared to the original, automated binning) to the respective subclusters, which were likely incorrectly binned by MaxBin.

**Table 1:** **Statistics of subclusters identified using VizBin for MaxBin-based bins 37B.out.024, 37B.out.026, and SRS013705.out.029**

**Subcluster**	**Number of contigs**	**Mbp**	**Single copy**	**Multiple copies**
37B.out.024.001	518	0.75	37	0
37B.out.024.002	1116	1.96	41	0
37B.out.026.001	569	0.79	12	3
37B.out.026.002	419	0.58	22	2
SRS013705.out.029.001	675	1.52	41	2
SRS013705.out.029.002	292	0.47	31	2
SRS013705.out.029.003	485	0.81	22	0
SRS013705.out.029.004	483	0.80	9	0
SRS013705.out.029.005	370	0.58	33	0

Copy numbers according to annotation of 107 single-copy marker genes.

Due to the pronounced heterogeneity observed for bin SRS013705.out.029, we also re-analyzed it using VizBin. The original SRS013705.out.029 bin separates into five distinct subclusters (Additional file 1: Figure S4) and the number of essential genes in multiple copies per subcluster is markedly reduced when separating these (Table 1; Additional file 1: Table S3). In particular, all but one subcluster are homogeneous, with SRS013705.out.029.001 being almost completely homogeneous.

Overall, the presented results demonstrate the potential of VizBin for the *post hoc* inspection and refinement of automatically generated bins.

### Runtimes

**Figure 4:**
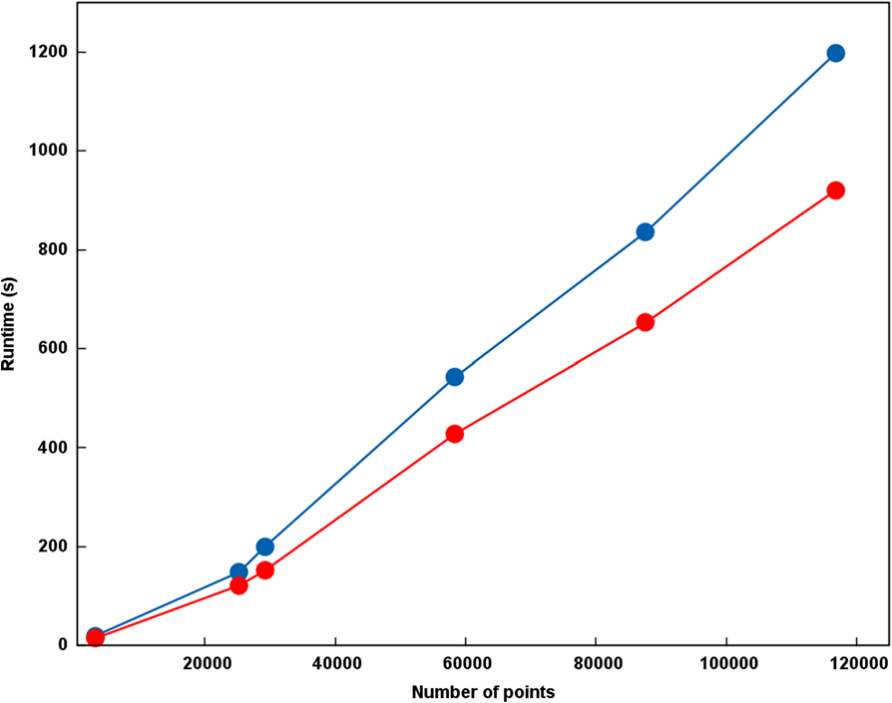
**VizBin runtimes.** Average (*n*=3) serial (one thread; blue color) and parallel (four threads; red colour) runtimes of VizBin on datasets of different size.

The implementation of BH-SNE which is incorporated into VizBin integrates the OpenMP®; API and therefore is able to utilize the multiple cores present in current desktop computers. Figure 4 illustrates the runtimes of VizBin using a single thread or four threads for datasets of different sizes. The runtime of VizBin for the groundwater metagenomic dataset (25,278 metagenomic fragments ≥ 1,000 nt; [20]) is reduced from around 150 s to around 120 s using four threads (MacBook Pro (Late 2011) with a 2.8GHz Intel Core i7, 8 GB 1333 MHz DDR3 memory, and Mac OS X Lion 10.7.5 (11G63b)). For a dataset comprising around 116,000 sequences, the visualization is obtained in 20 min, with time savings of ≥20% (15.3 min) when using four threads. This highlights the scalability of the herein presented tool for the efficient visualization and possible subsequent binning of ever-growing metagenomic datasets both in terms of number and size.

## Conclusions

Here, we present VizBin, an easy-to-use, stand-alone software application for the visualization, inspection, and human-augmented binning of metagenomic datasets from single samples. The presented results demonstrate that the VizBin software can be applied to metagenomic data from various environments and leverages the superior pattern recognition capabilities of the human eye-brain system for cluster identification and delineation. In addition to its use for *de novo* human-augmented binning of metagenomic data [14], VizBin holds great potential for the *post hoc* inspection and subsequent refinement of automatically generated bins. This is illustrated by the increased homogeneity and increased completeness in the case of metagenomic data derived from cellulolytic or human-borne microbial consortia. Furthermore, the herein-presented software application improves on the computational efficiency of the approach previously described in [14] by the integration of parallelization. Two-dimensional embeddings are obtained in less than 3 min for datasets of ≈30,000 fragments on a common, multi-core desktop computer. Moreover, our results demonstrate that despite recent advances in automated unsupervised binning of individual samples, as represented by MaxBin [16], improved results can be obtained through efficient visualization of the entire community and/or of automatically generated bins. MetaWatt [21] and GroopM [22] are two recent approaches which involve human input for the definition or refinement of metagenomic bins. However, MetaWatt has been demonstrated for microbial communities with relatively small numbers of binnable populations, and GroopM is a representative of a set of recent approaches for automated unsupervised binning which rely on abundance information across several samples [23,24]. Abundance-based approaches may not be generally applicable for metagenomic analysis of microbial consortia due to various reasons, such as limited sample quantities or prohibitive costs of analysing the numbers of samples required (e.g., a suggested minimum of 18 samples in [23]). While a minimum number of three related samples is suggested for GroopM [22], VizBin allows for the characterization of single samples. We are currently exploring ways to integrate coverage information (from single samples) into the dimension reduction step as it is expected to provide another important and likely informative feature for the visualization and subsequent binning of metagenomic data. At the present time, as described above, sequence coverage from the metagenomic assembly of a single sample as well as other information may optionally be provided to VizBin to enhance scatter plot visualization.

## Availability and requirements

**Project name:** VizBin**Project home page:**https://claczny.github.io/VizBin**Operating system(s):** Platform independent**Programming language:** Java™ version 7 or greater**Other requirements:** Java™ Standard Edition Runtime Environment (JRE); local installation of BLAS/LAPACK for maximum performance (detailed information is provided in the project’s wiki)**License:** BSD License (4-clause). Detailed licensing information is available at https://github.com/claczny/VizBin.**Restrictions:** None

## Additional files

Additional file 1 **VizBin supplementary materials.**

Additional file 2 **ZIP archive containing VizBin visualization screenshots of the individual bins for the three datasets (37A, 37B, and SRS013705) originally reported in [** 16 **].**

## Abbreviations

SOM: Self-organizing map
ESOM: Emergent-SOM
BH-SNE: Barnes-Hut stochastic neighbor embedding
GUI: Graphical user interface
API: Application programming interface
JRE: Java™ standard edition runtime environment
