# Supplementary material for: VizBin - an application for reference-independent visualization and human-augmented binning of metagenomic data
Source: Microbiome. 2015 Jan 20;3:1. doi: 10.1186/s40168-014-0066-1 (PMC4305225; doi:10.1186/s40168-014-0066-1)
Supplement: Supplementary file 1 — VizBin supplementary materials. [file 40168_2014_66_MOESM1_ESM.pdf]

VizBin - An application for reference-independent visualization & human-augmented binning of metagenomic data

Cedric C. Laczny[1], Tomasz Sternal[2], Valentin Plugaru[3], Piotr Gawron[1], Arash Atashpendar[3], Houry Hera Margossian[3], Sergio Coronado[1], Laurens van der Maaten[4], Nikos Vlassis[5], Paul Wilmes[1,*]
1 Luxembourg Centre for Systems Biomedicine, University of Luxembourg, Esch-sur-Alzette, Luxembourg
2 Institute of Computing Science, Poznan University of Technology, Poznan, Poland
3 Computer Science and Communications Research Unit, University of Luxembourg, Luxembourg, Luxembourg
4 Pattern Recognition and Bioinformatics Group, Delft University of Technology, CD Delft, Netherlands
5 Adobe Research, Adobe, San Jose, United States of America
∗ E-mail: Correspondence to be addressed to paul.wilmes@uni.lu

Contents

List of Supplementary Figures

S1 ESOM-based visualization of metagenomic dataset 37A - Cellulolytic microbial community 3
S2 VizBin-based visualizations of homogeneous MaxBin-based bins 4
S3 VizBin-based visualizations of heterogeneous MaxBin-based bins 5
S4 VizBin-based visualizations of metagenomic dataset SRS013705 - Human tongue dorsum-derived 6

List of Supplementary Tables

S1 Essential gene analysis results of the original 37A bins 7
S2 Essential gene analysis results of the original 37B bins 7
S3 Essential gene analysis results of the original SRS013705 bins 8

Supplementary Figures

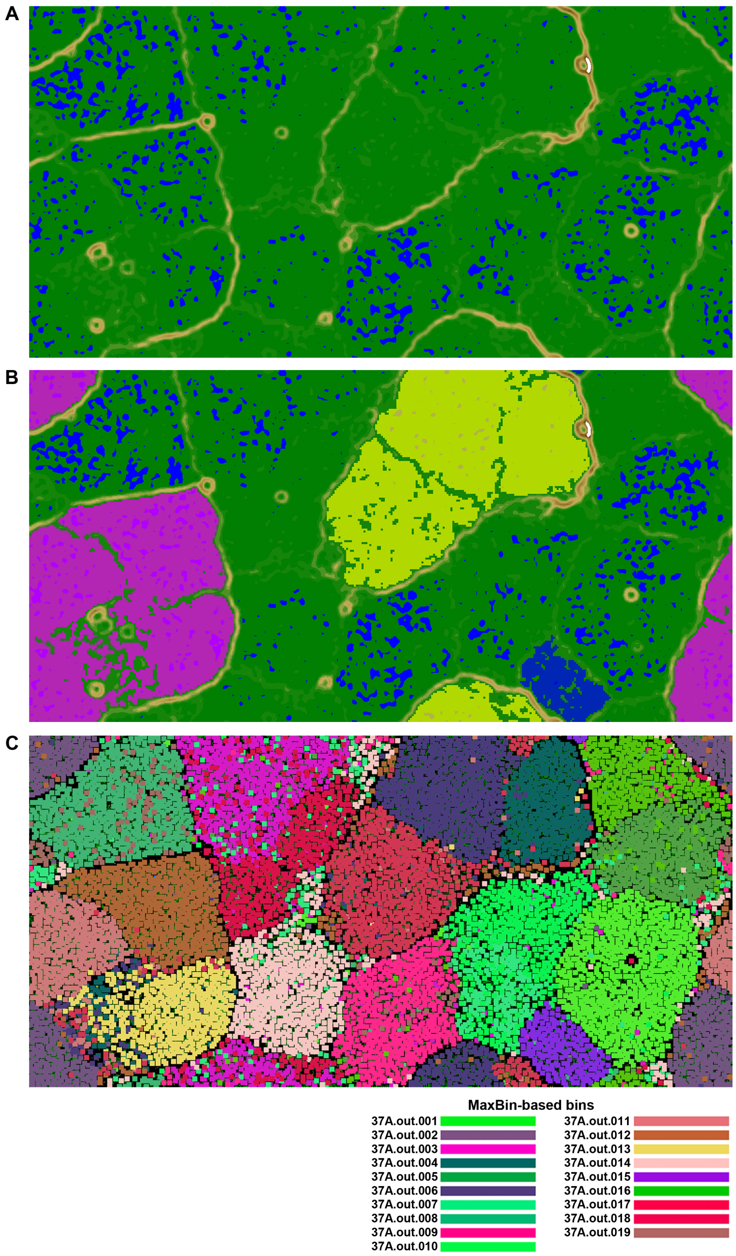

Figure S1: ESOM-based visualization of a cellulolytic microbial community metagenomic dataset (37A) [1]. (A) Topological map. Parameters for the ESOM computation according to [2], specifically: k-Batch algorithm (k = 0.15), number of rows = 182, number of columns = 364. All other parameters were left default. Minimal fragment length (in bases): 2,000, window size 5,000. Visualized maps were generated using software available at https://github.com/tetramerFreqs/Binning and the Databionics ESOM Suite (http://databionic-esom.sourceforge.net/). Runtime: 3,114 seconds. (B) Selection using the flood-fill algorithm at a threshold of 0.1. (C) Colouring based on the original MaxBin-based assignment [1]. Colours match those in Figure 2.

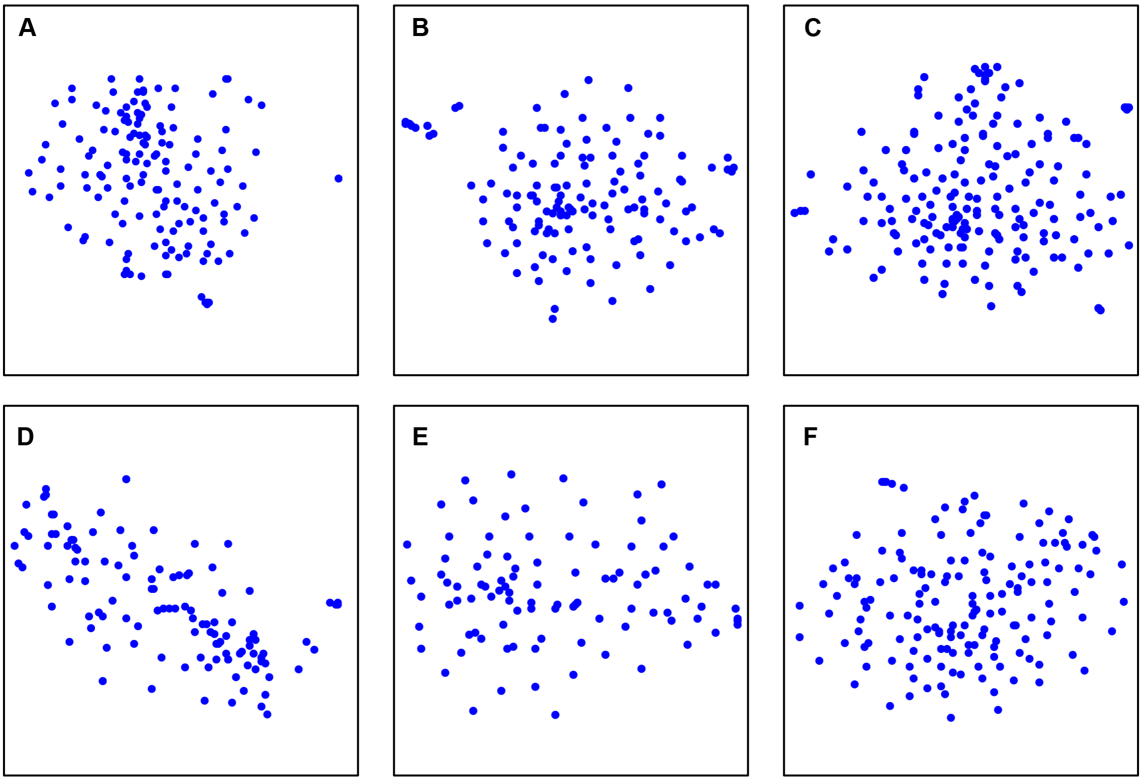

Figure S2: VizBin-based visualizations of homogeneous MaxBin-based bins from different metagenomic datasets. (A) 37A.out.001. (B) 37A.out.002. (C) 37B.out.001. (D) 37B.out.002. (E) 37B.out.003. (F) SRS013705.out.005.

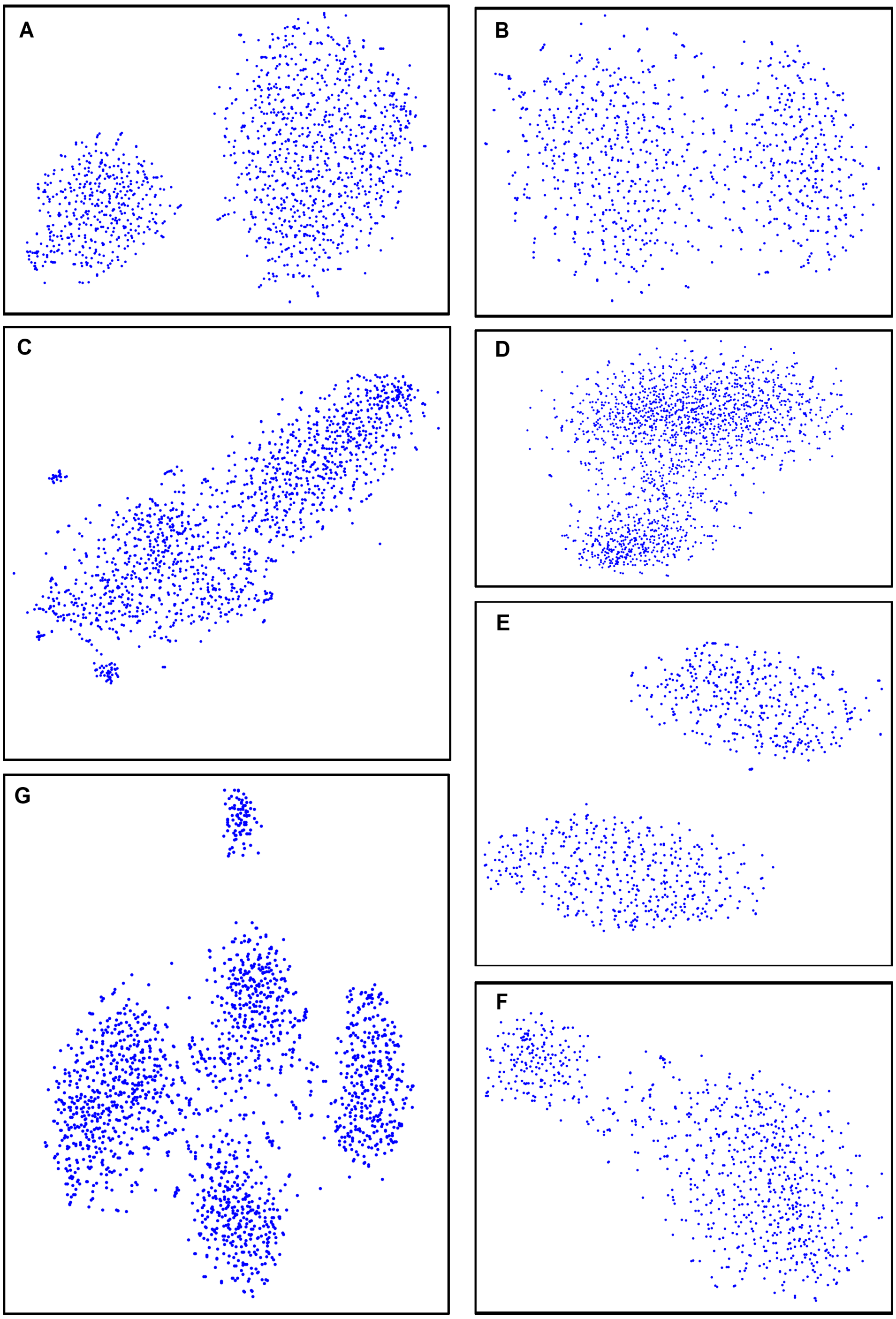

Figure S3: VizBin-based visualizations of heterogeneous MaxBin-based bins from different metagenomic datasets. Visualizations have been zoomed out twice for display purposes. (A) 37B.out.024. (B) 37B.out.026. (C) 37A.out.014. (D) 37A.out.018. (E) SRS013705.out.004. (F) SRS013705.out.026. (G) SRS013705.out.029 (rotated 90° clockwise for display purposes).

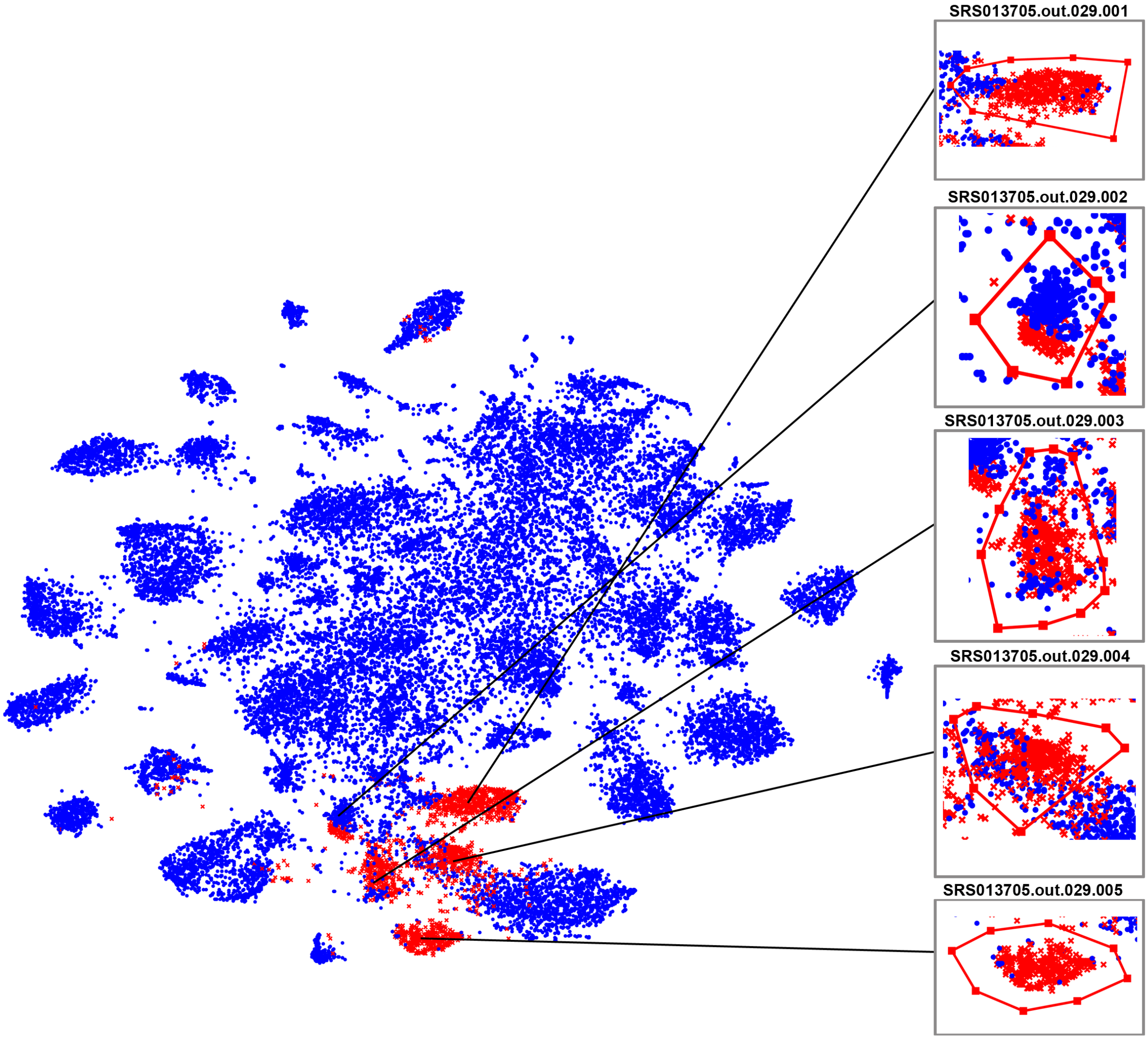

Figure S4: VizBin-based visualizations of metagenomic dataset SRS013705, i.e., human tongue dorsum-derived [3]. Points highlighted in red represent contigs originally assigned to MaxBin bin SRS013705.out.029. Inserts are closeups of polygonal selections in VizBin and the resulting five subclusters: SRS013705.out.029.001, SRS013705.out.029.002, SRS013705.out.029.003, SRS013705.out.029.004, and SRS013705.out.029.005.

Supplementary Tables

Table S1: Essential gene analysis results of the original 37A bins as reported in [1].

**Bin**	**Single copy**	**Multiple copies**
37A.out.001	97	7
37A.out.002	101	4
37A.out.003	82	12
37A.out.004	88	17
37A.out.005	87	8
37A.out.006	96	8
37A.out.007	61	5
37A.out.008	89	3
37A.out.009	66	6
37A.out.010	50	46
37A.out.011	97	3
37A.out.012	90	11
37A.out.013	98	3
37A.out.014	67	22
37A.out.015	50	2
37A.out.016	45	9
37A.out.017	39	6
37A.out.018	62	21
37A.out.019	23	1

Table S2: Essential gene analysis results of the original 37B bins as reported in [1].

**Bin**	**Single copy**	**Multiple copies**
37B.out.001	103	1
37B.out.002	101	4
37B.out.003	103	0
37B.out.004	90	1
37B.out.005	85	12
37B.out.006	98	7
37B.out.007	52	0
37B.out.008	58	43
37B.out.009	97	6
37B.out.010	98	4
37B.out.011	99	7
37B.out.012	100	5
37B.out.013	97	4
37B.out.014	98	4
37B.out.015	51	3
37B.out.016	50	1
37B.out.017	99	3
37B.out.018	37	23
37B.out.019	22	10
37B.out.020	53	12
37B.out.021	20	6
37B.out.022	34	33
37B.out.023	33	26
37B.out.024	45	28
37B.out.025	63	3
37B.out.026	12	5

Table S3: Essential gene analysis results of the original SRS013705 bins as reported in [1].

Bin	Single copy	Multiple copies
SRS013705.out.001	30	20
SRS013705.out.002	41	17
SRS013705.out.003	50	7
SRS013705.out.004	49	18
SRS013705.out.005	82	1
SRS013705.out.006	31	9
SRS013705.out.007	58	31
SRS013705.out.008	44	33
SRS013705.out.009	36	36
SRS013705.out.010	75	24
SRS013705.out.011	20	3
SRS013705.out.012	22	19
SRS013705.out.013	89	15
SRS013705.out.014	86	7
SRS013705.out.015	27	2
SRS013705.out.016	7	5
SRS013705.out.017	70	4
SRS013705.out.018	66	7
SRS013705.out.019	41	29
SRS013705.out.020	43	22
SRS013705.out.021	30	6
SRS013705.out.022	68	5
SRS013705.out.023	54	29
SRS013705.out.024	43	34
SRS013705.out.025	33	30
SRS013705.out.026	83	17
SRS013705.out.027	28	13
SRS013705.out.028	24	13
SRS013705.out.029	36	33
SRS013705.out.030	47	3
SRS013705.out.031	39	19

References

[1] Wu, Y.-W., Tang, Y.-H., Tringe, S. G., Simmons, B. A. & Singer, S. W. MaxBin: an automated binning method to recover individual genomes from metagenomes using an expectation-maximization algorithm. *Microbiome* **2**, 26 (2014).

[2] Dick, G. J. *et al.* Community-wide analysis of microbial genome sequence signatures. *Genome Biol* **10**, R85 (2009).

[3] The Human Microbiome Project Consortium. Structure, function and diversity of the healthy human microbiome. *Nature* **486**, 207–14 (2012).
