# Supplementary figures and images for: VizBin - an application for reference-independent visualization and human-augmented binning of metagenomic data
Source: Microbiome. 2015 Jan 20;3:1. doi: 10.1186/s40168-014-0066-1 (PMC4305225; doi:10.1186/s40168-014-0066-1)

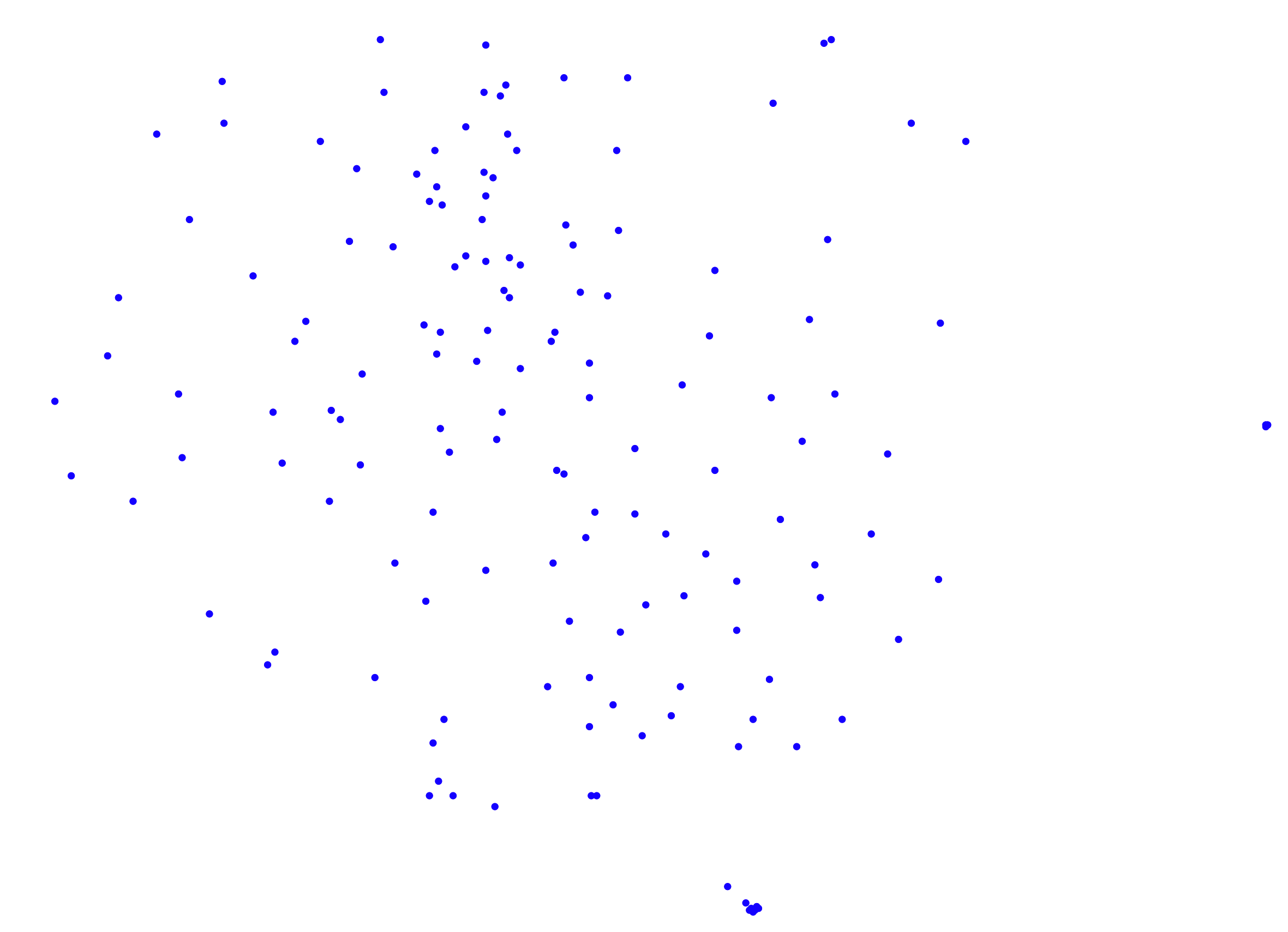

Supplement: Supplementary file 2 — ZIP archive containing VizBin visualization screenshots of the individual bins for the three datasets (37A, 37B, and SRS013705) originally reported in [ 16 ]. [file 40168_2014_66_MOESM2_ESM.zip › 37A_37B_SRS013705/37A/37A.out.001.png]

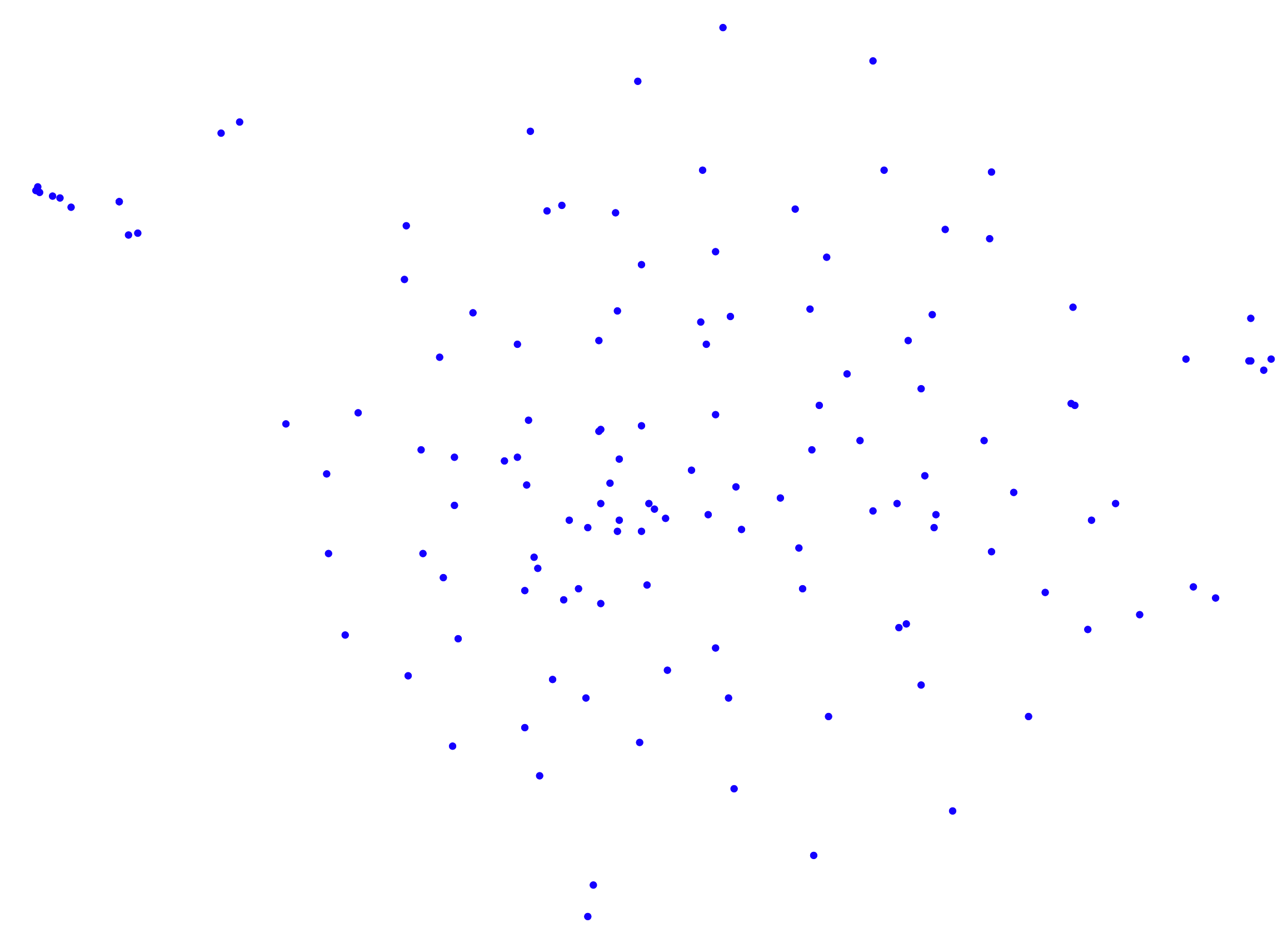

Supplement: Supplementary file 2 — ZIP archive containing VizBin visualization screenshots of the individual bins for the three datasets (37A, 37B, and SRS013705) originally reported in [ 16 ]. [file 40168_2014_66_MOESM2_ESM.zip › 37A_37B_SRS013705/37A/37A.out.002.png]

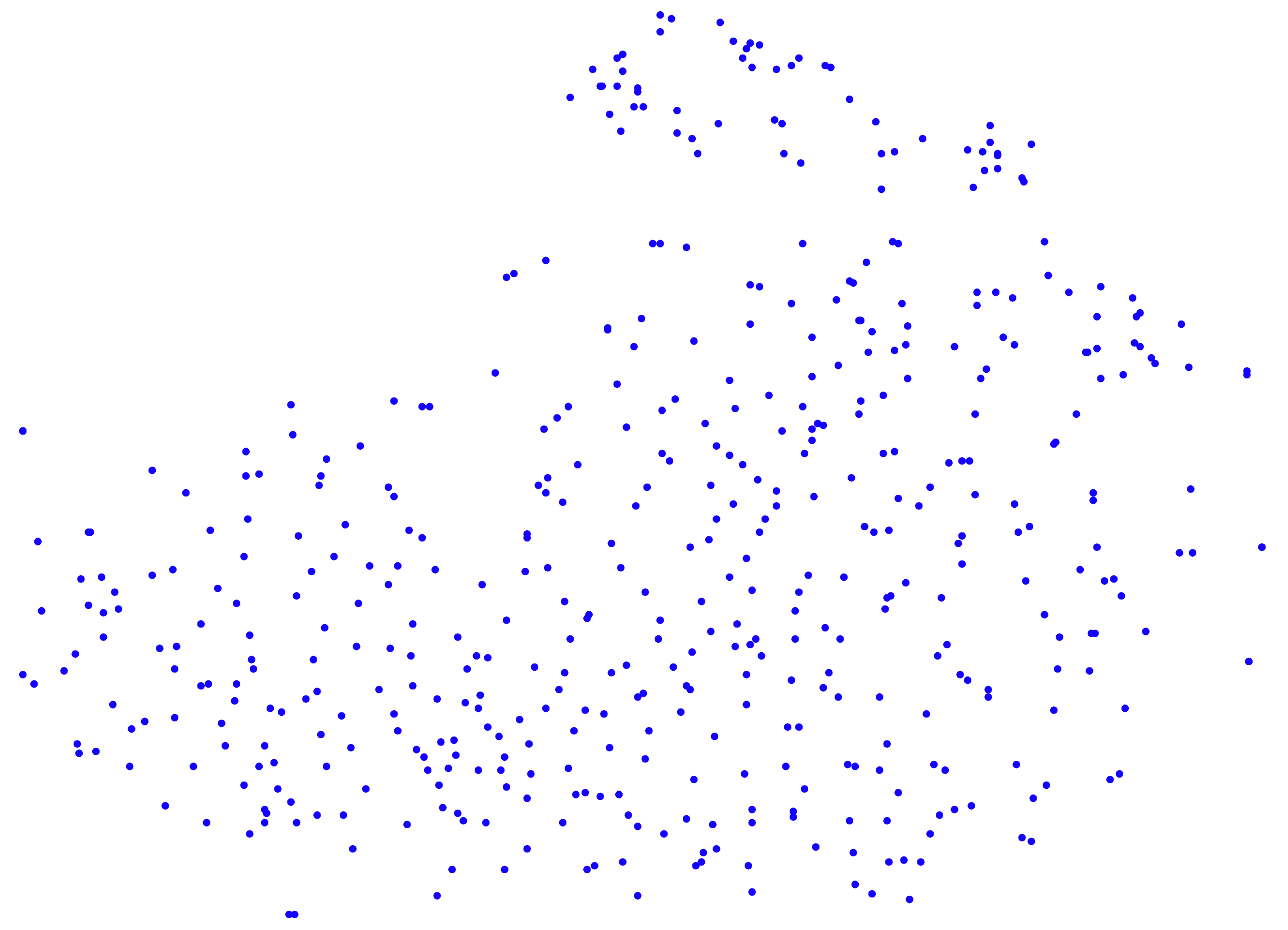

Supplement: Supplementary file 2 — ZIP archive containing VizBin visualization screenshots of the individual bins for the three datasets (37A, 37B, and SRS013705) originally reported in [ 16 ]. [file 40168_2014_66_MOESM2_ESM.zip › 37A_37B_SRS013705/37A/37A.out.003.png]

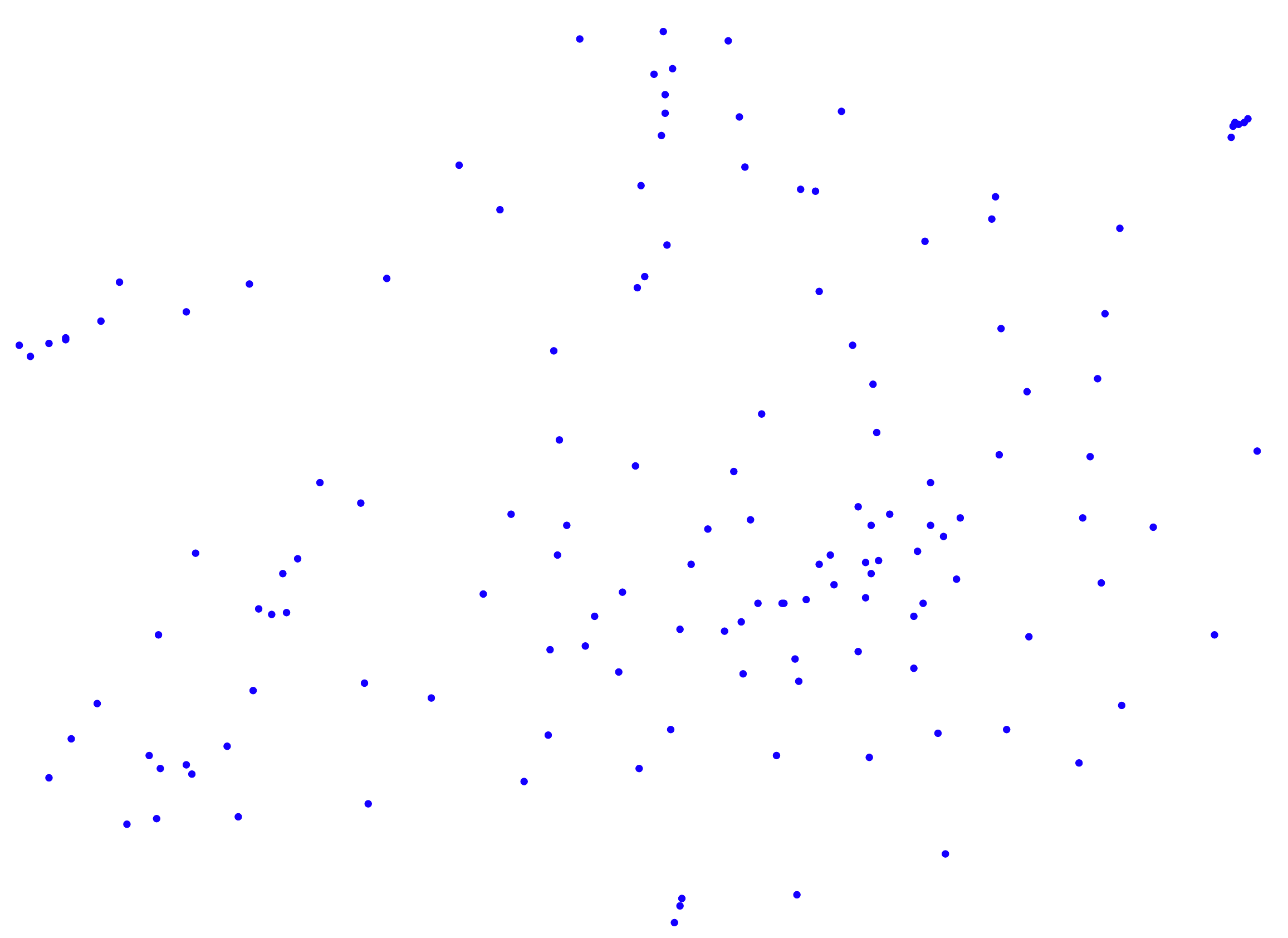

Supplement: Supplementary file 2 — ZIP archive containing VizBin visualization screenshots of the individual bins for the three datasets (37A, 37B, and SRS013705) originally reported in [ 16 ]. [file 40168_2014_66_MOESM2_ESM.zip › 37A_37B_SRS013705/37A/37A.out.004.png]

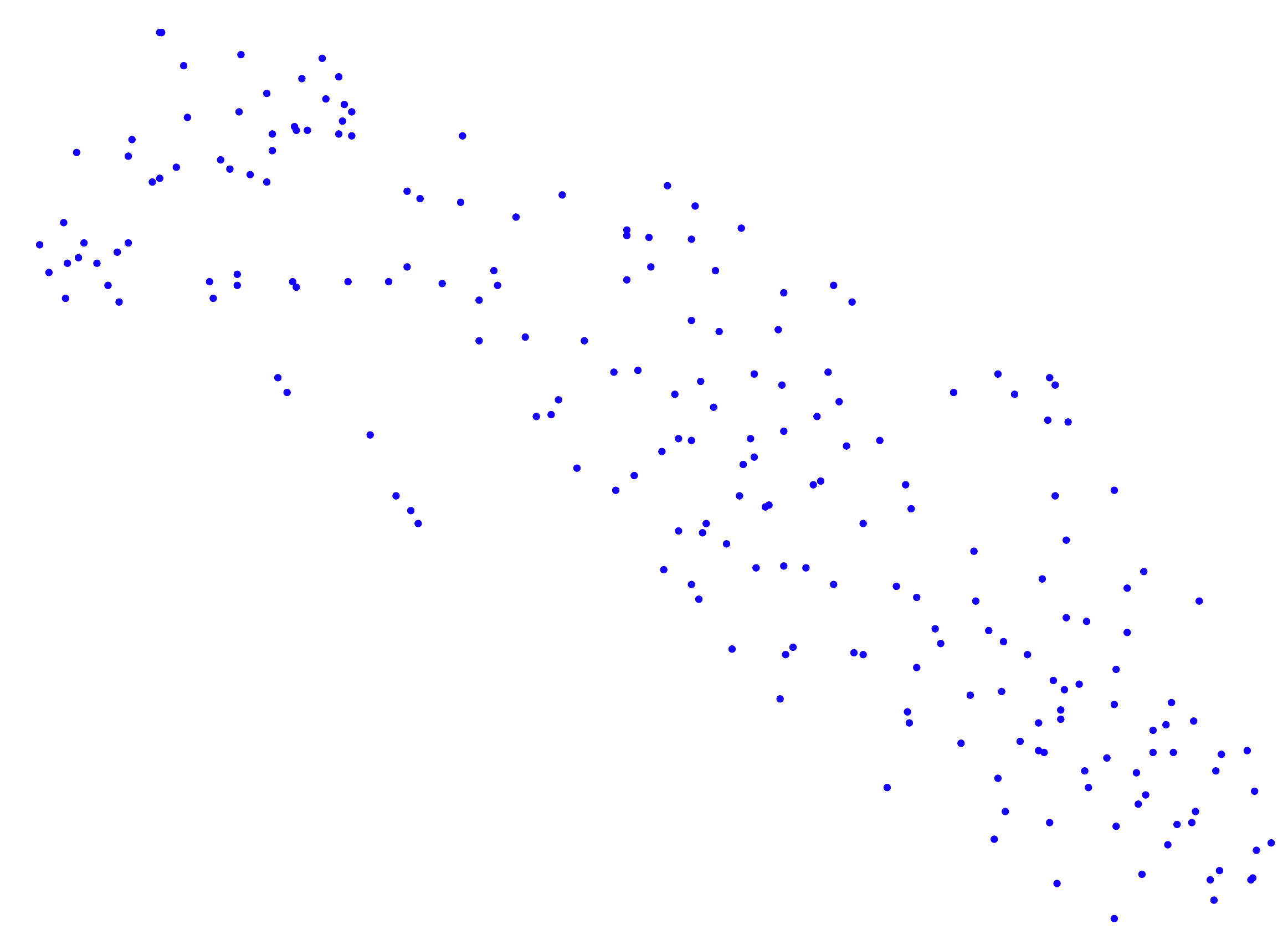

Supplement: Supplementary file 2 — ZIP archive containing VizBin visualization screenshots of the individual bins for the three datasets (37A, 37B, and SRS013705) originally reported in [ 16 ]. [file 40168_2014_66_MOESM2_ESM.zip › 37A_37B_SRS013705/37A/37A.out.005.png]

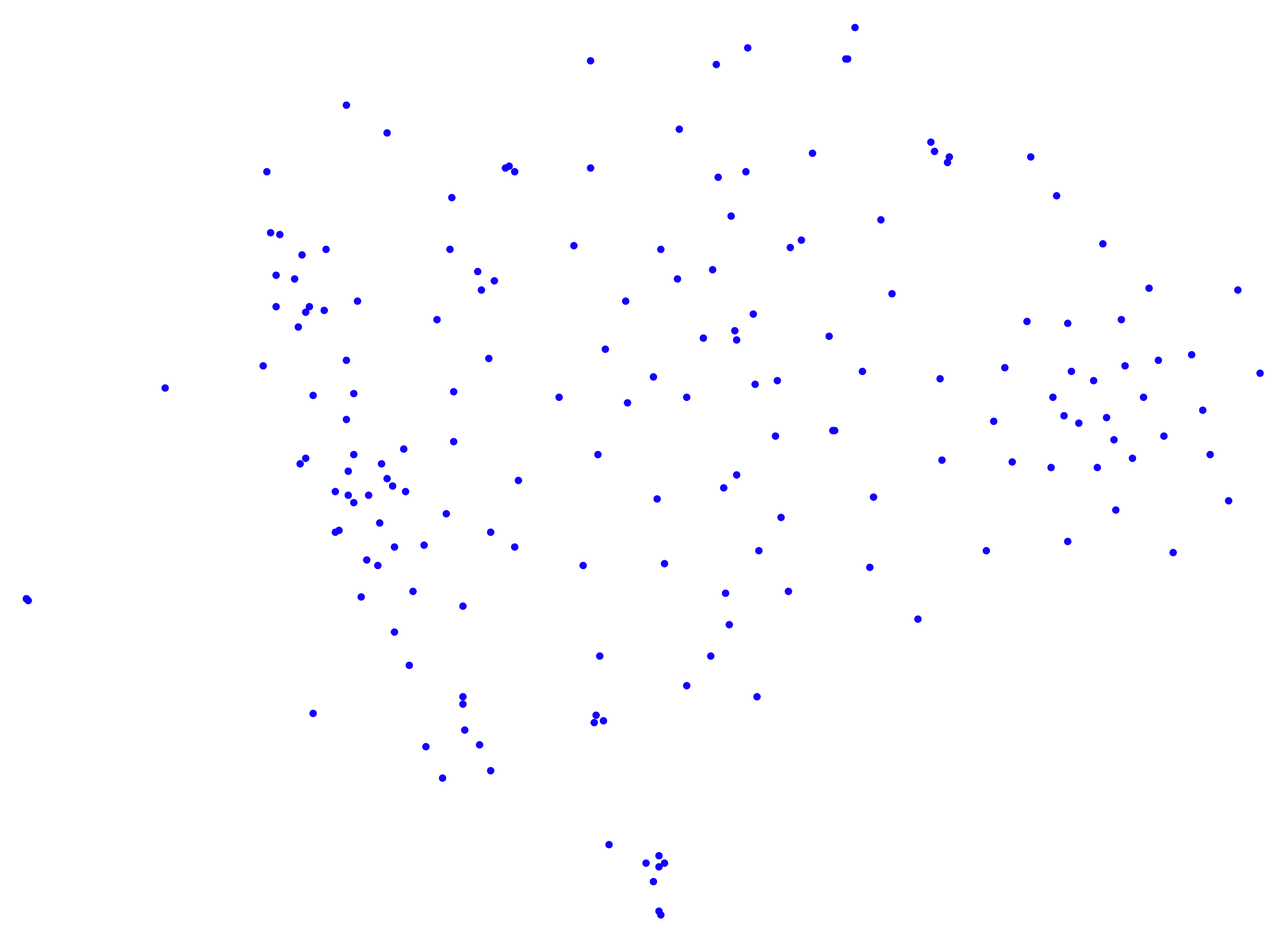

Supplement: Supplementary file 2 — ZIP archive containing VizBin visualization screenshots of the individual bins for the three datasets (37A, 37B, and SRS013705) originally reported in [ 16 ]. [file 40168_2014_66_MOESM2_ESM.zip › 37A_37B_SRS013705/37A/37A.out.006.png]

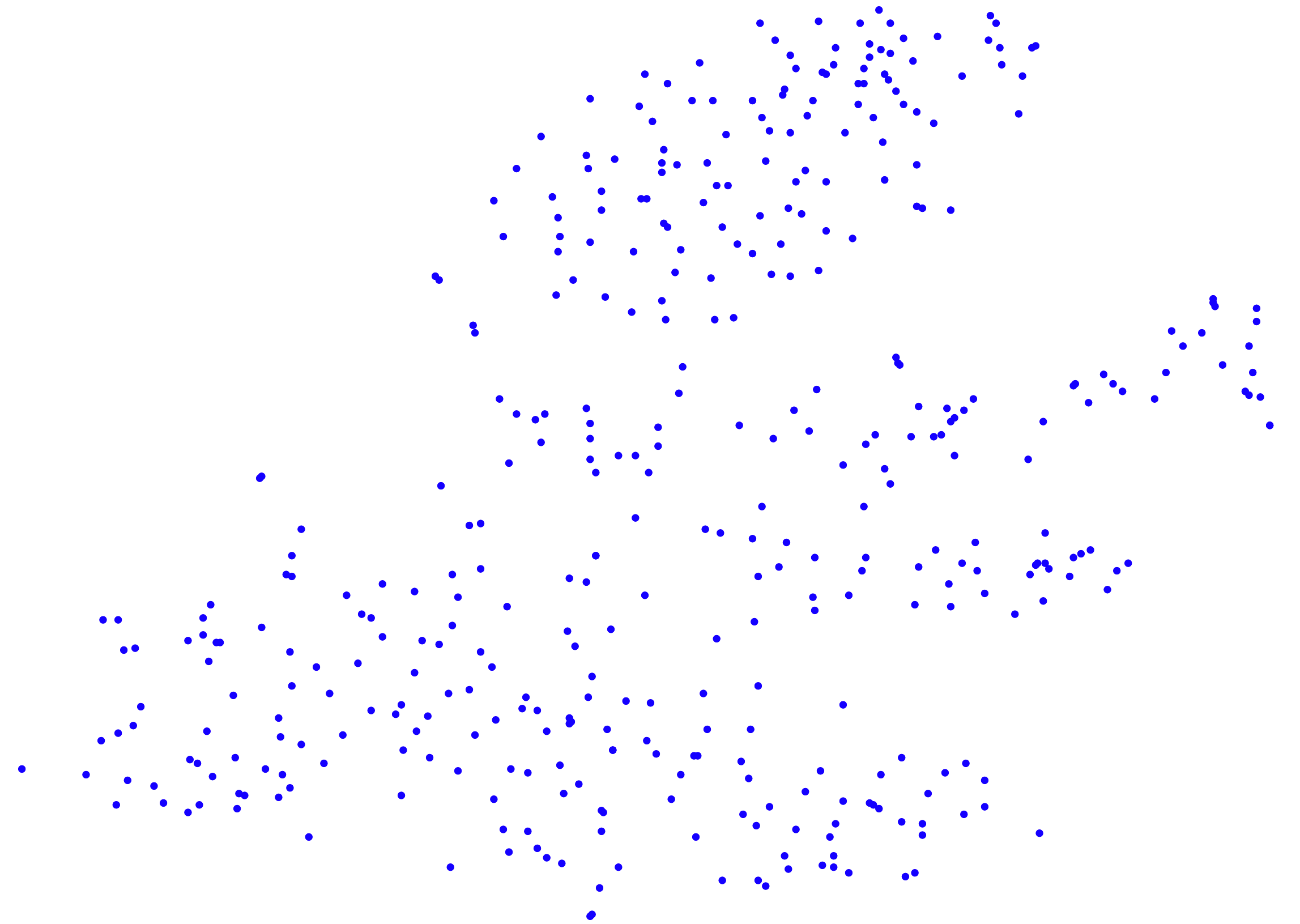

Supplement: Supplementary file 2 — ZIP archive containing VizBin visualization screenshots of the individual bins for the three datasets (37A, 37B, and SRS013705) originally reported in [ 16 ]. [file 40168_2014_66_MOESM2_ESM.zip › 37A_37B_SRS013705/37A/37A.out.007.png]

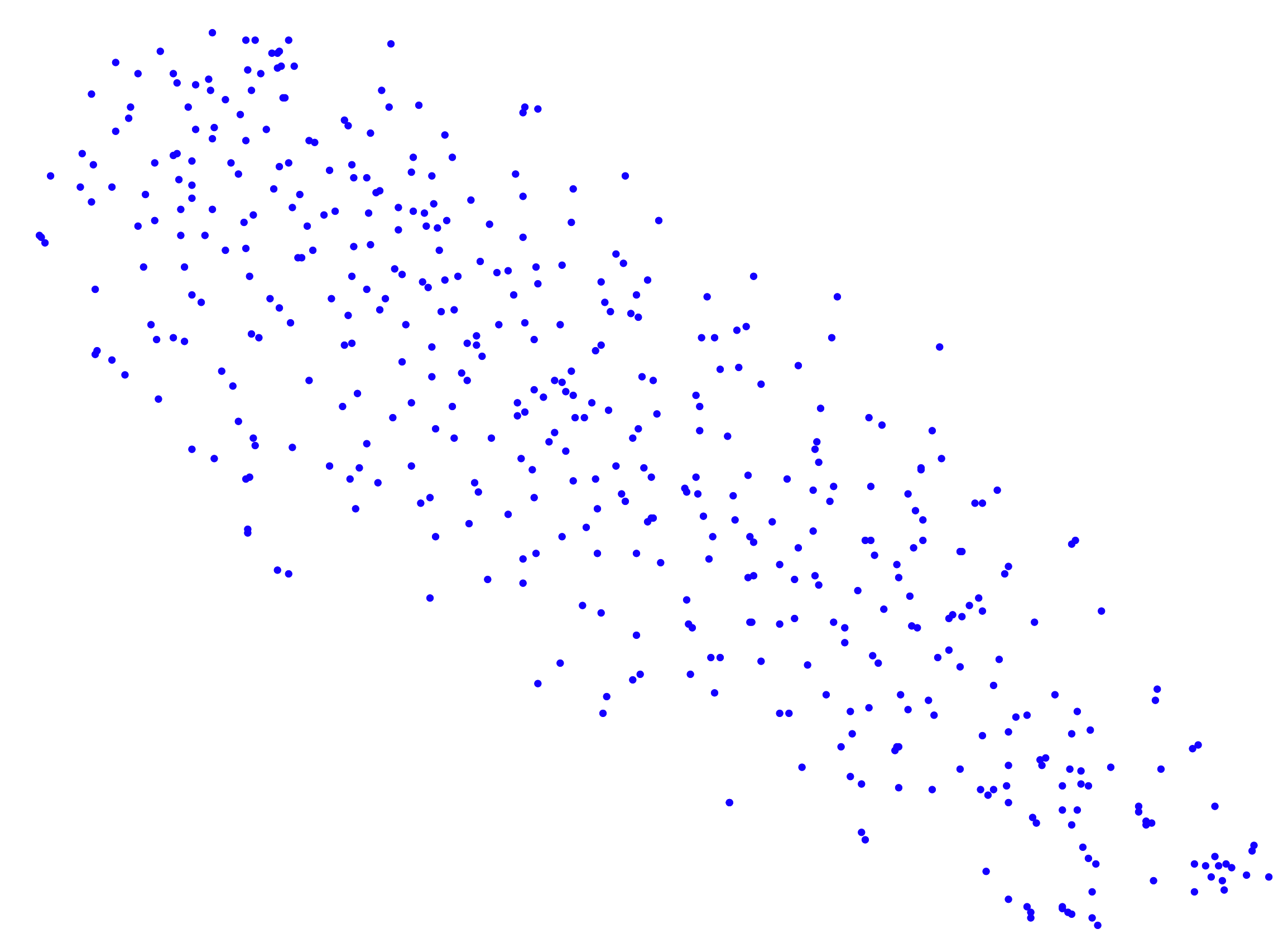

Supplement: Supplementary file 2 — ZIP archive containing VizBin visualization screenshots of the individual bins for the three datasets (37A, 37B, and SRS013705) originally reported in [ 16 ]. [file 40168_2014_66_MOESM2_ESM.zip › 37A_37B_SRS013705/37A/37A.out.008.png]

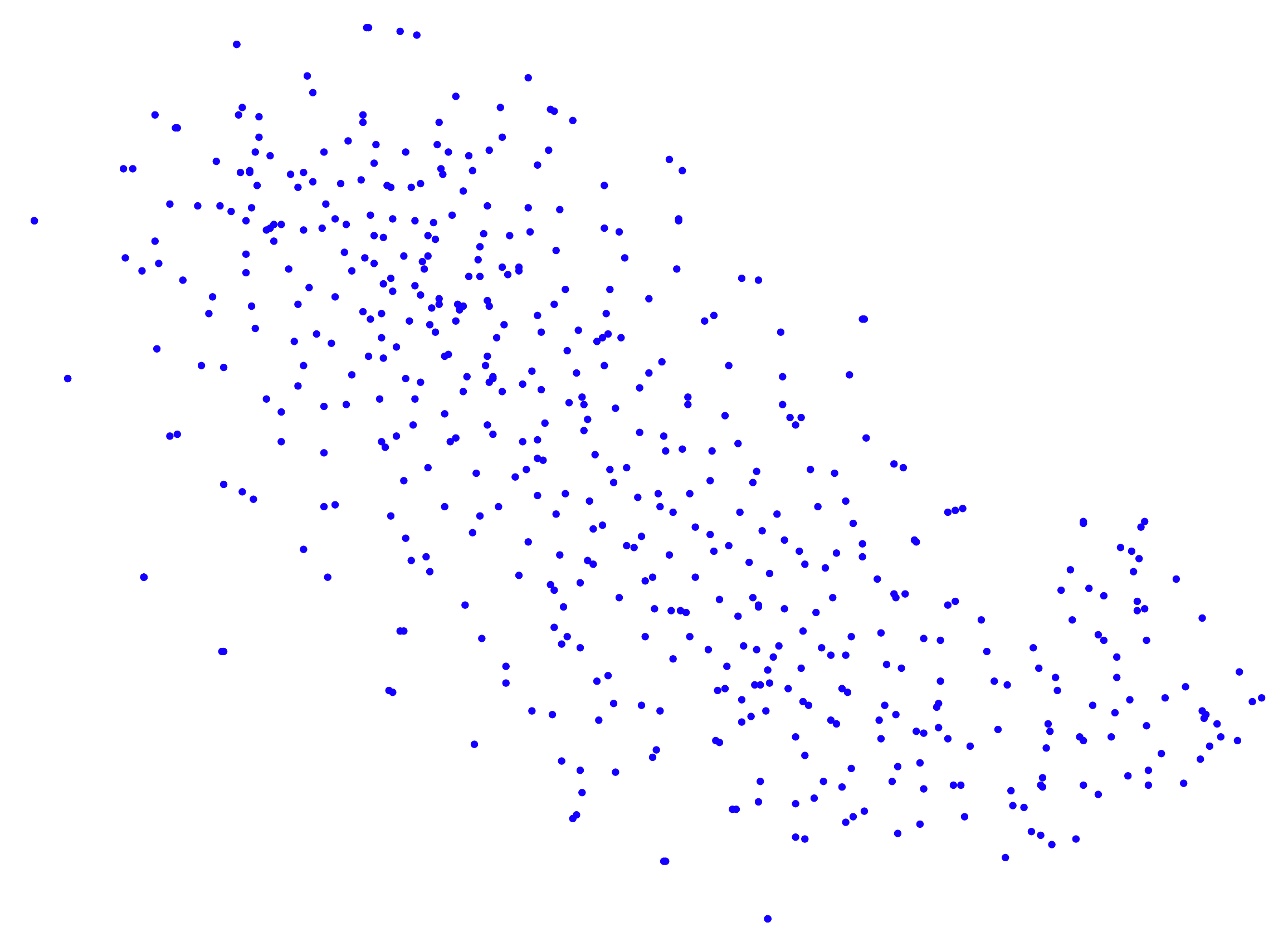

Supplement: Supplementary file 2 — ZIP archive containing VizBin visualization screenshots of the individual bins for the three datasets (37A, 37B, and SRS013705) originally reported in [ 16 ]. [file 40168_2014_66_MOESM2_ESM.zip › 37A_37B_SRS013705/37A/37A.out.009.png]

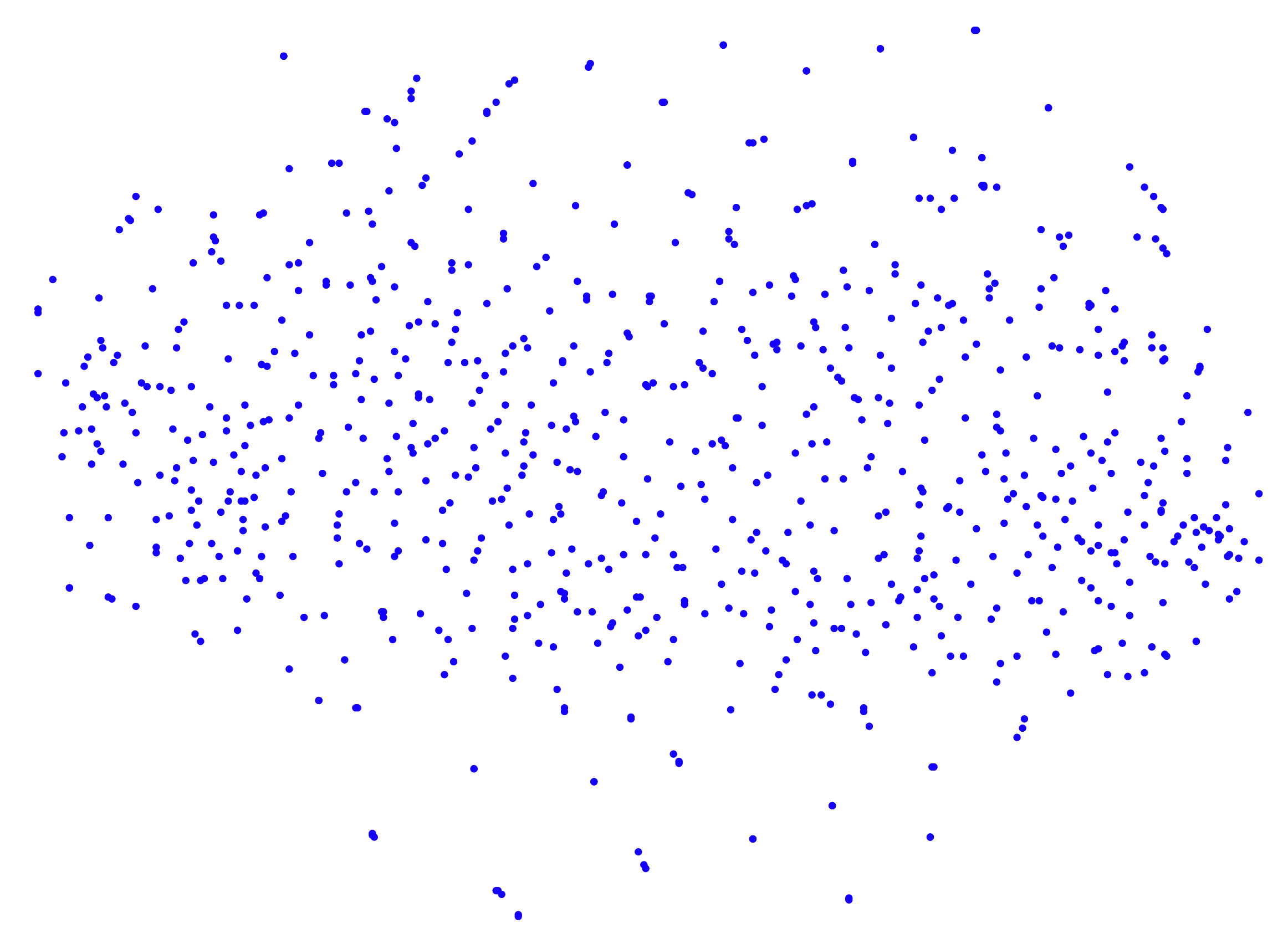

Supplement: Supplementary file 2 — ZIP archive containing VizBin visualization screenshots of the individual bins for the three datasets (37A, 37B, and SRS013705) originally reported in [ 16 ]. [file 40168_2014_66_MOESM2_ESM.zip › 37A_37B_SRS013705/37A/37A.out.010.png]

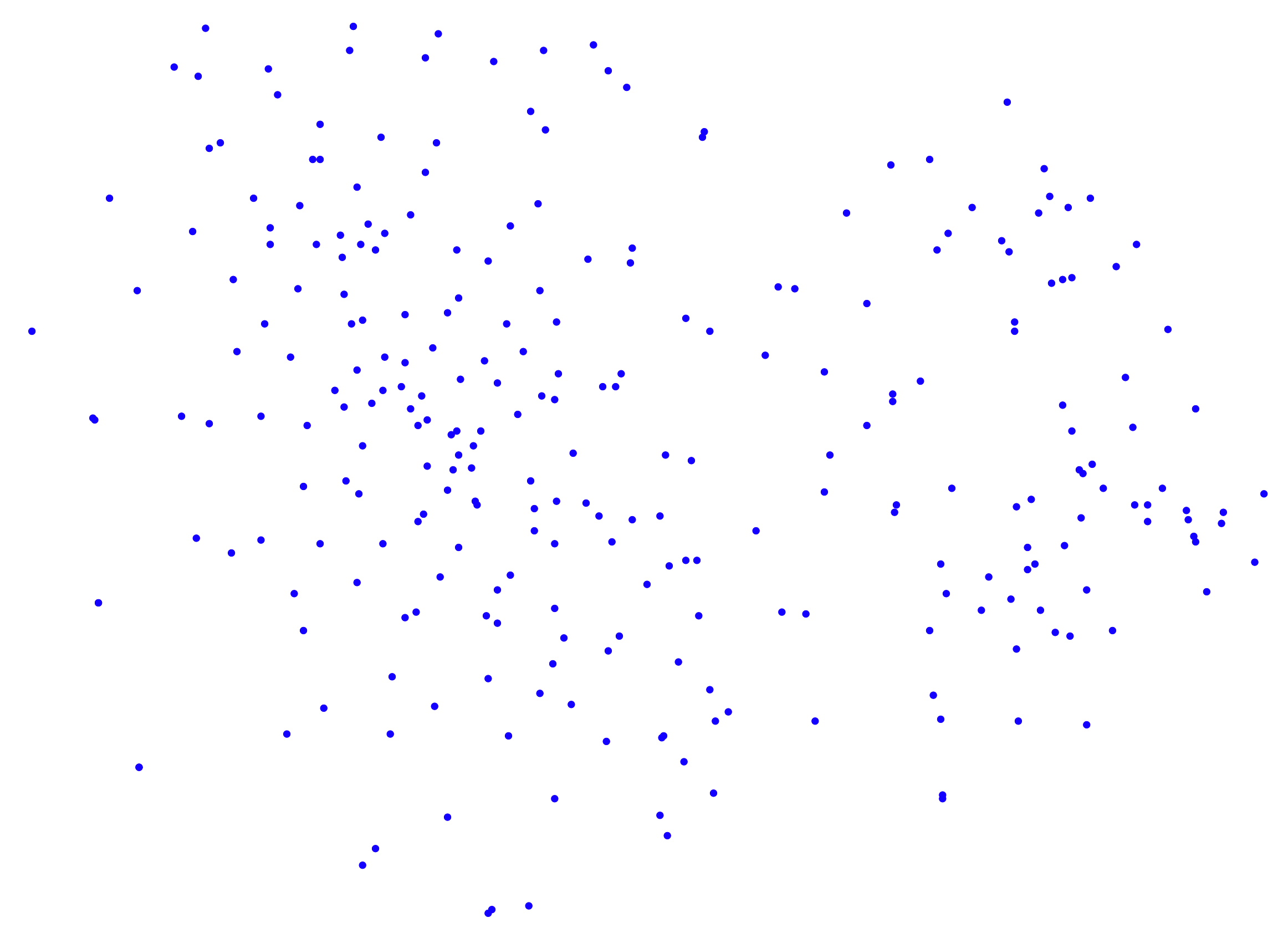

Supplement: Supplementary file 2 — ZIP archive containing VizBin visualization screenshots of the individual bins for the three datasets (37A, 37B, and SRS013705) originally reported in [ 16 ]. [file 40168_2014_66_MOESM2_ESM.zip › 37A_37B_SRS013705/37A/37A.out.011.png]

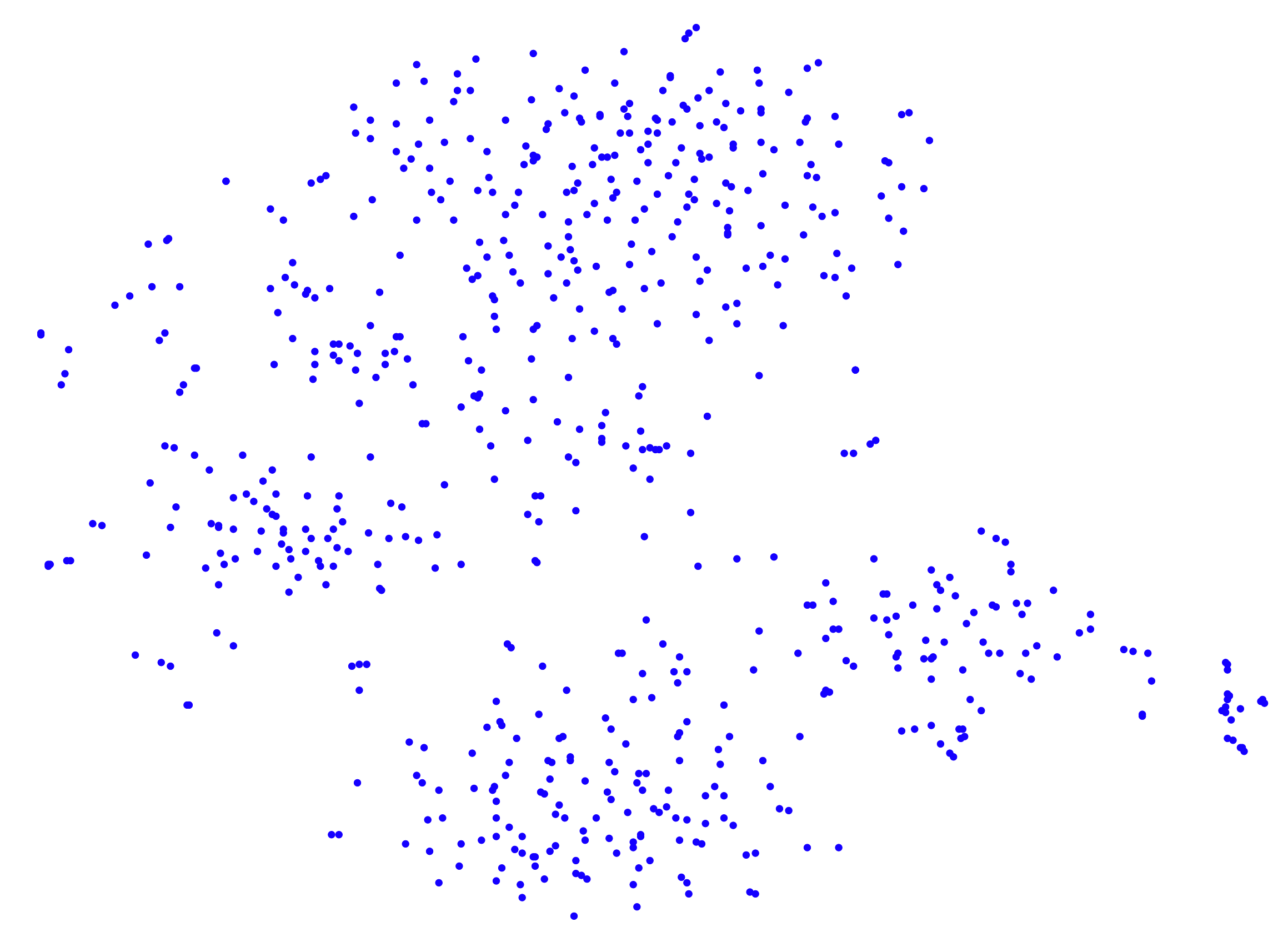

Supplement: Supplementary file 2 — ZIP archive containing VizBin visualization screenshots of the individual bins for the three datasets (37A, 37B, and SRS013705) originally reported in [ 16 ]. [file 40168_2014_66_MOESM2_ESM.zip › 37A_37B_SRS013705/37A/37A.out.012.png]

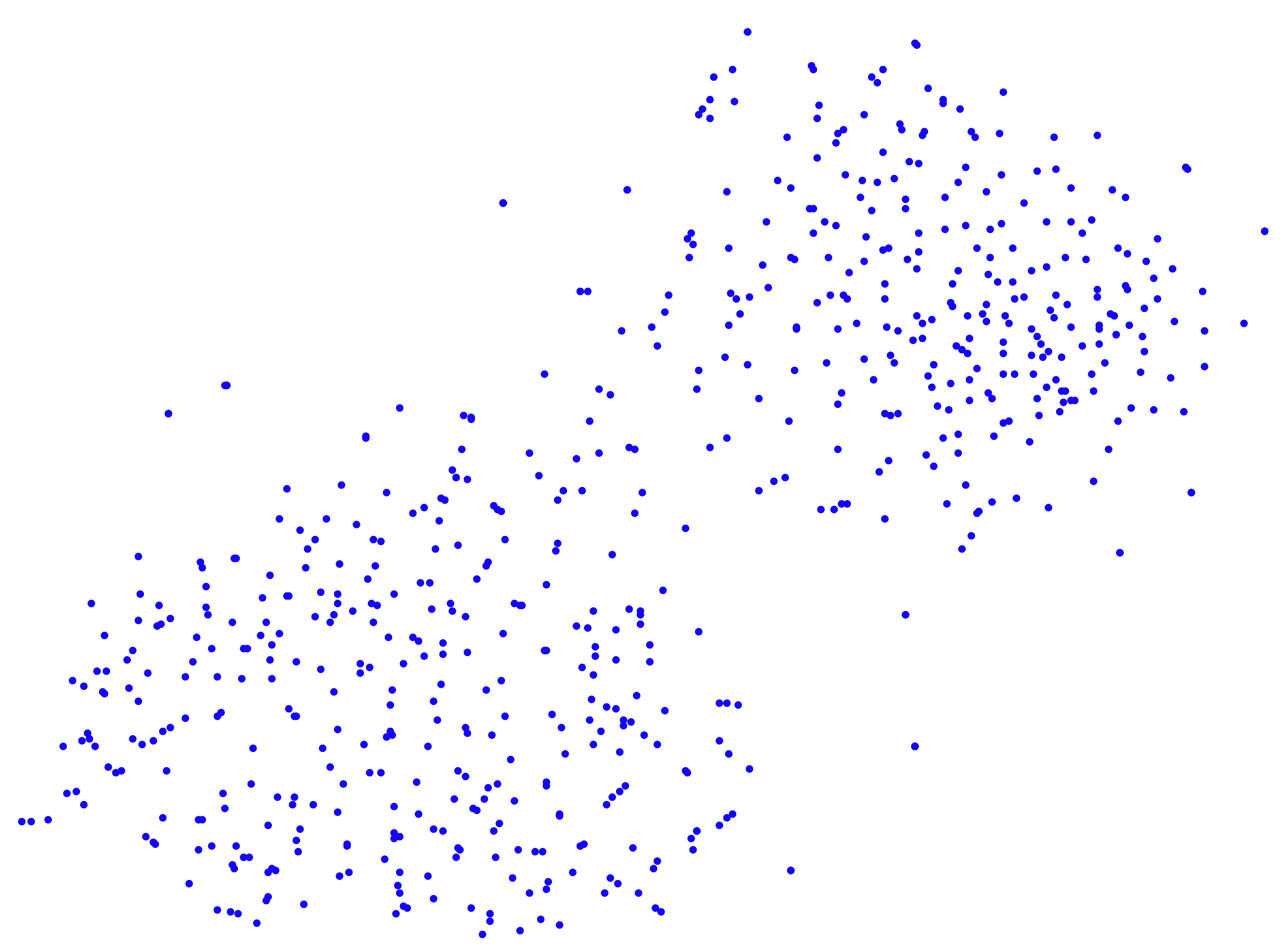

Supplement: Supplementary file 2 — ZIP archive containing VizBin visualization screenshots of the individual bins for the three datasets (37A, 37B, and SRS013705) originally reported in [ 16 ]. [file 40168_2014_66_MOESM2_ESM.zip › 37A_37B_SRS013705/37A/37A.out.013.png]

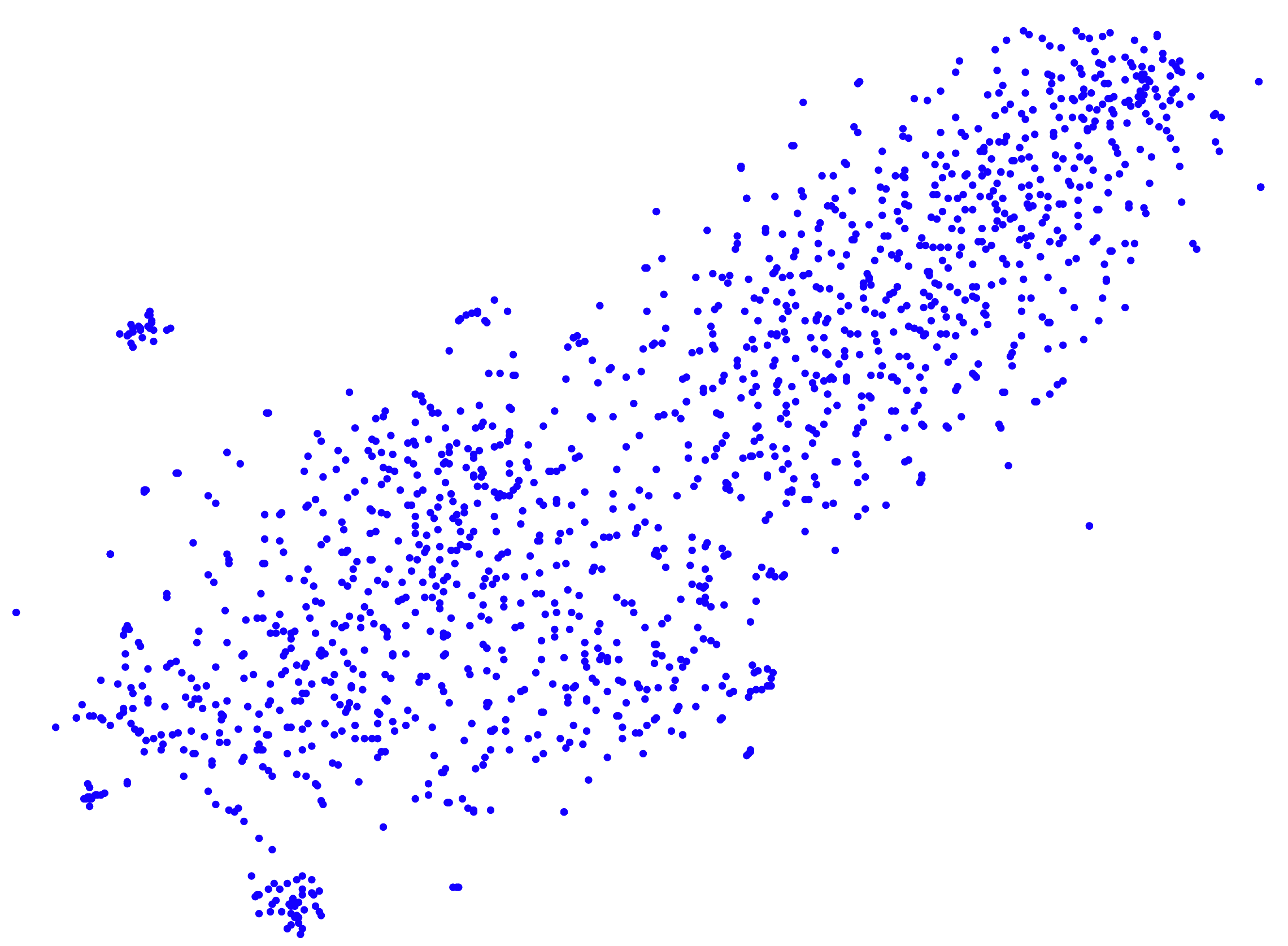

Supplement: Supplementary file 2 — ZIP archive containing VizBin visualization screenshots of the individual bins for the three datasets (37A, 37B, and SRS013705) originally reported in [ 16 ]. [file 40168_2014_66_MOESM2_ESM.zip › 37A_37B_SRS013705/37A/37A.out.014.png]

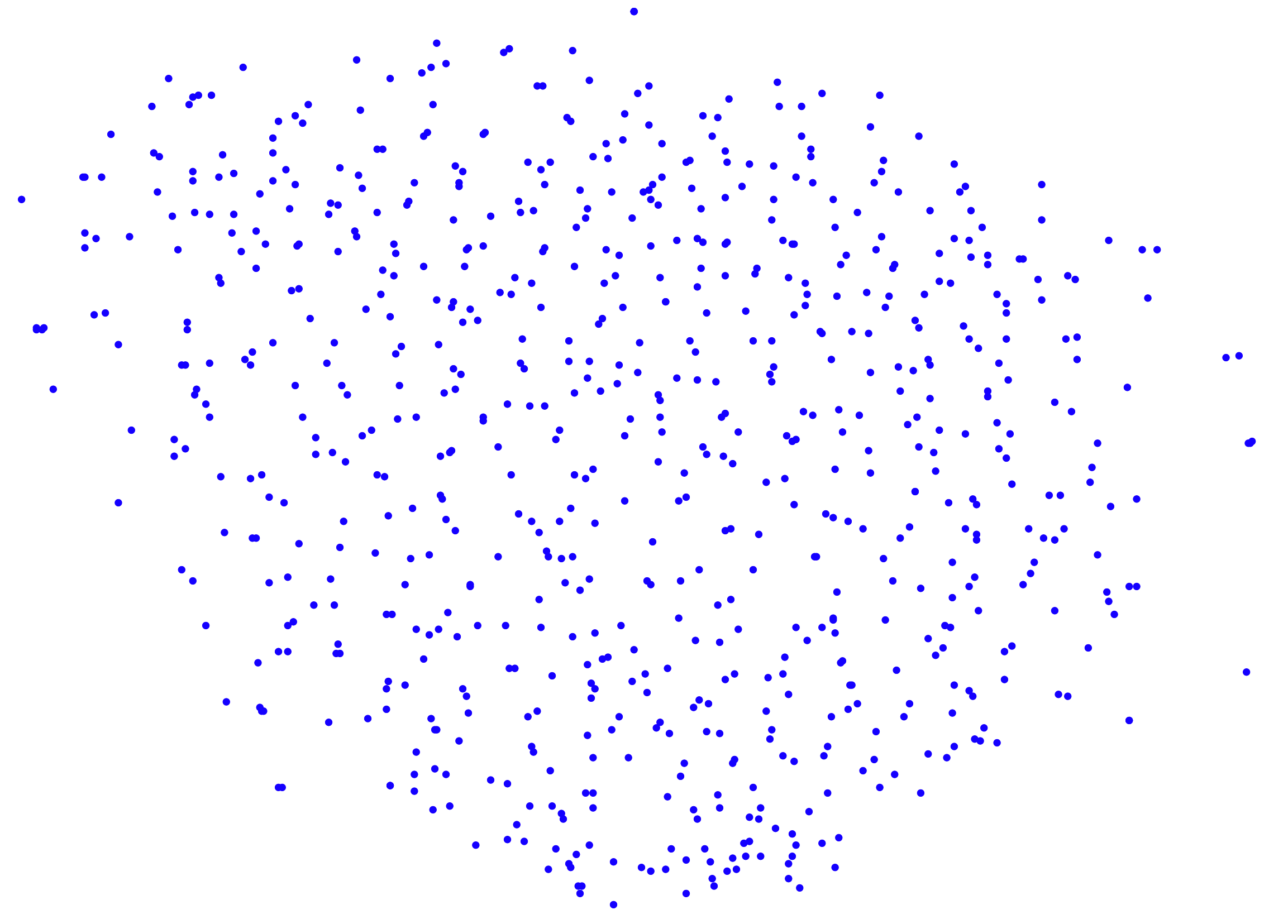

Supplement: Supplementary file 2 — ZIP archive containing VizBin visualization screenshots of the individual bins for the three datasets (37A, 37B, and SRS013705) originally reported in [ 16 ]. [file 40168_2014_66_MOESM2_ESM.zip › 37A_37B_SRS013705/37A/37A.out.015.png]

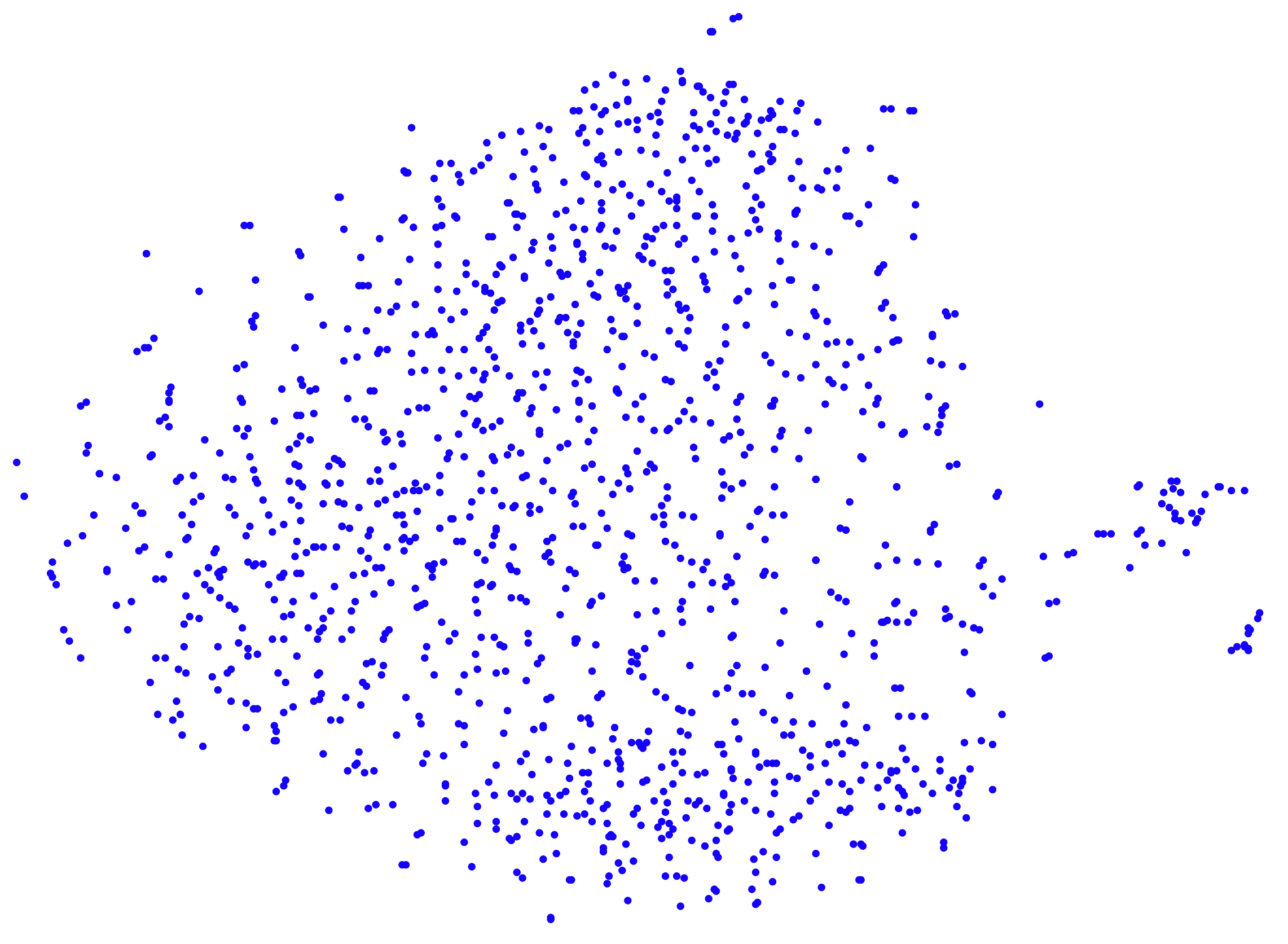

Supplement: Supplementary file 2 — ZIP archive containing VizBin visualization screenshots of the individual bins for the three datasets (37A, 37B, and SRS013705) originally reported in [ 16 ]. [file 40168_2014_66_MOESM2_ESM.zip › 37A_37B_SRS013705/37A/37A.out.016.png]

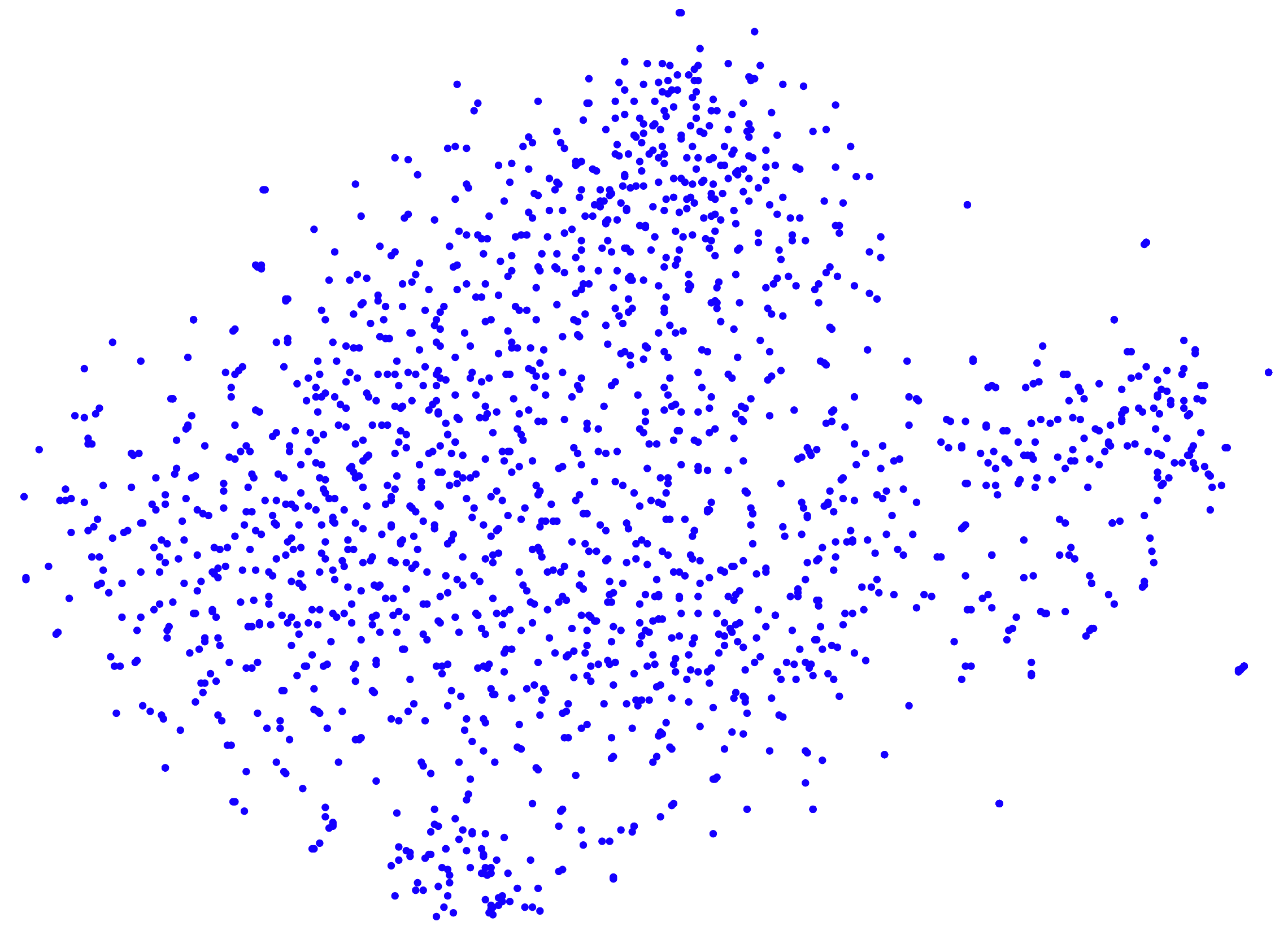

Supplement: Supplementary file 2 — ZIP archive containing VizBin visualization screenshots of the individual bins for the three datasets (37A, 37B, and SRS013705) originally reported in [ 16 ]. [file 40168_2014_66_MOESM2_ESM.zip › 37A_37B_SRS013705/37A/37A.out.017.png]

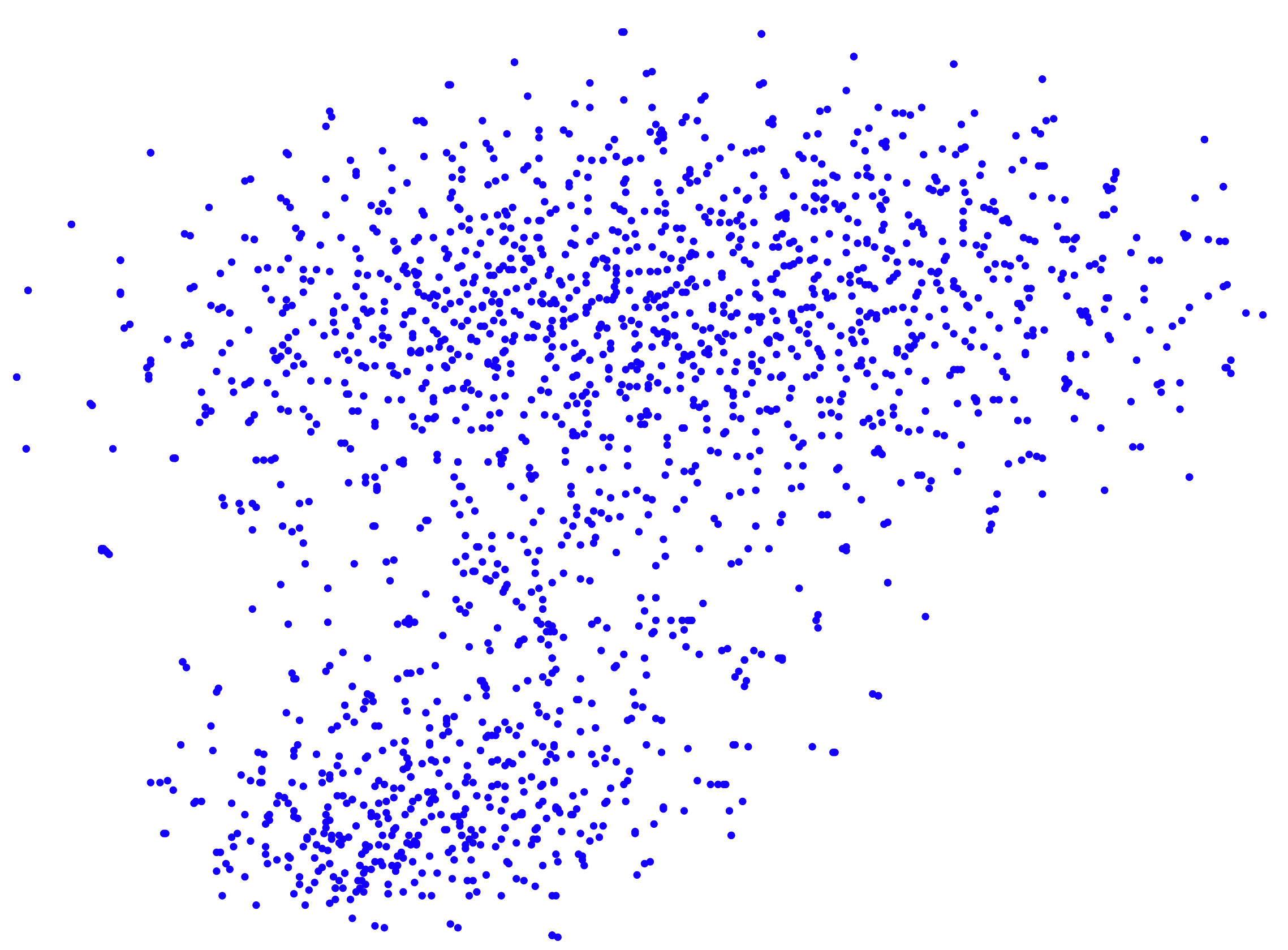

Supplement: Supplementary file 2 — ZIP archive containing VizBin visualization screenshots of the individual bins for the three datasets (37A, 37B, and SRS013705) originally reported in [ 16 ]. [file 40168_2014_66_MOESM2_ESM.zip › 37A_37B_SRS013705/37A/37A.out.018.png]

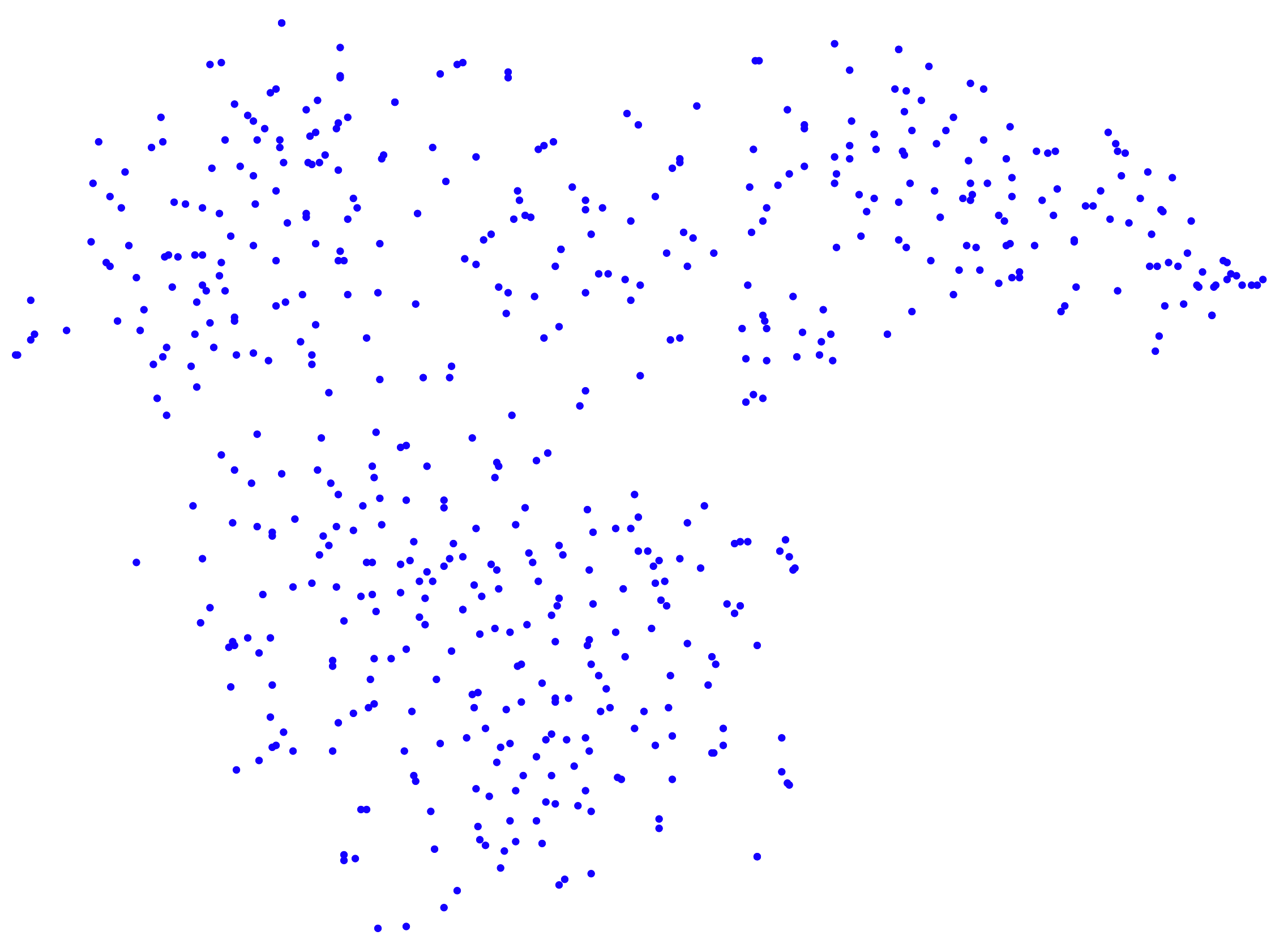

Supplement: Supplementary file 2 — ZIP archive containing VizBin visualization screenshots of the individual bins for the three datasets (37A, 37B, and SRS013705) originally reported in [ 16 ]. [file 40168_2014_66_MOESM2_ESM.zip › 37A_37B_SRS013705/37A/37A.out.019.png]

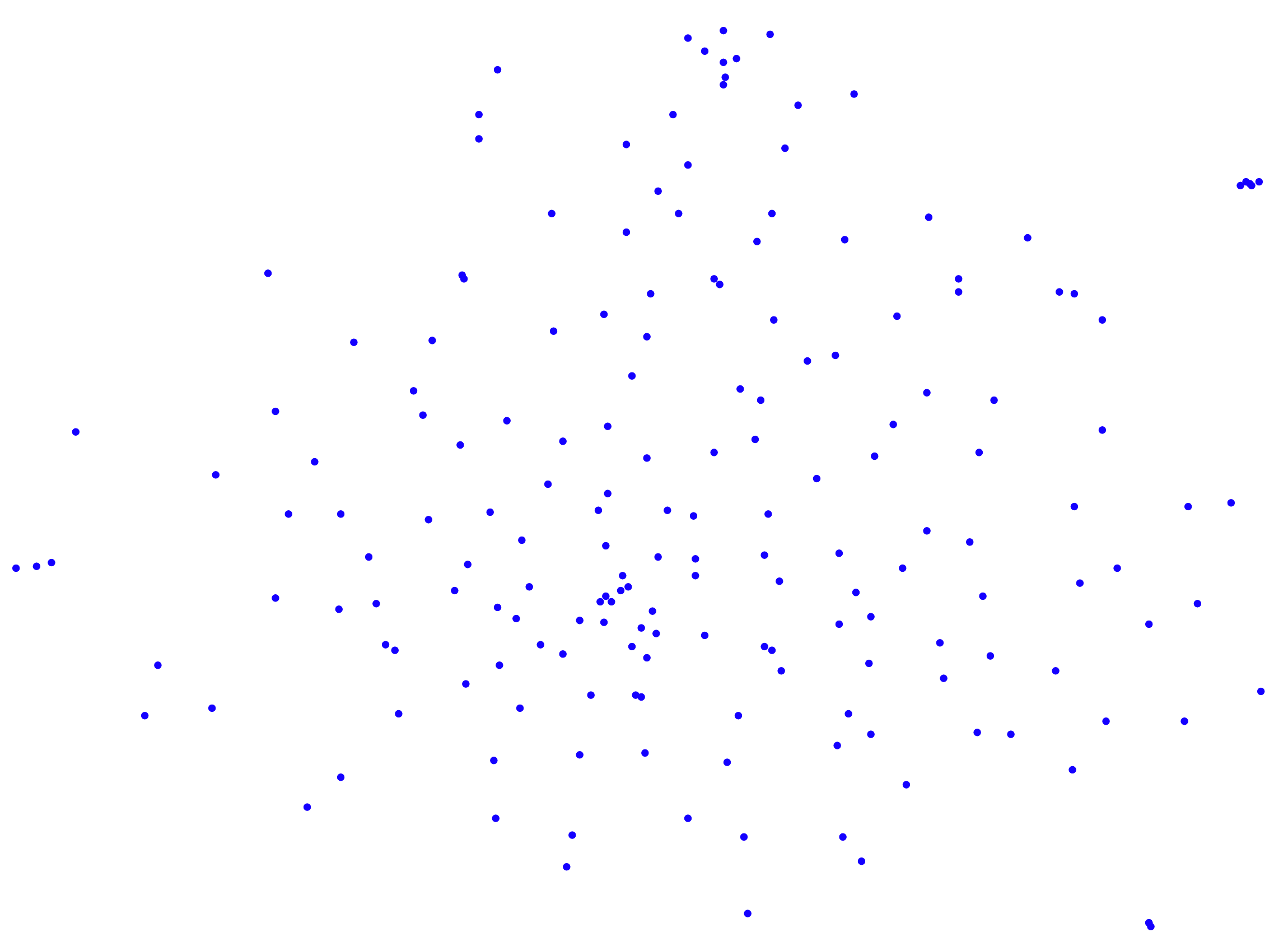

Supplement: Supplementary file 2 — ZIP archive containing VizBin visualization screenshots of the individual bins for the three datasets (37A, 37B, and SRS013705) originally reported in [ 16 ]. [file 40168_2014_66_MOESM2_ESM.zip › 37A_37B_SRS013705/37B/37B.out.001.png]

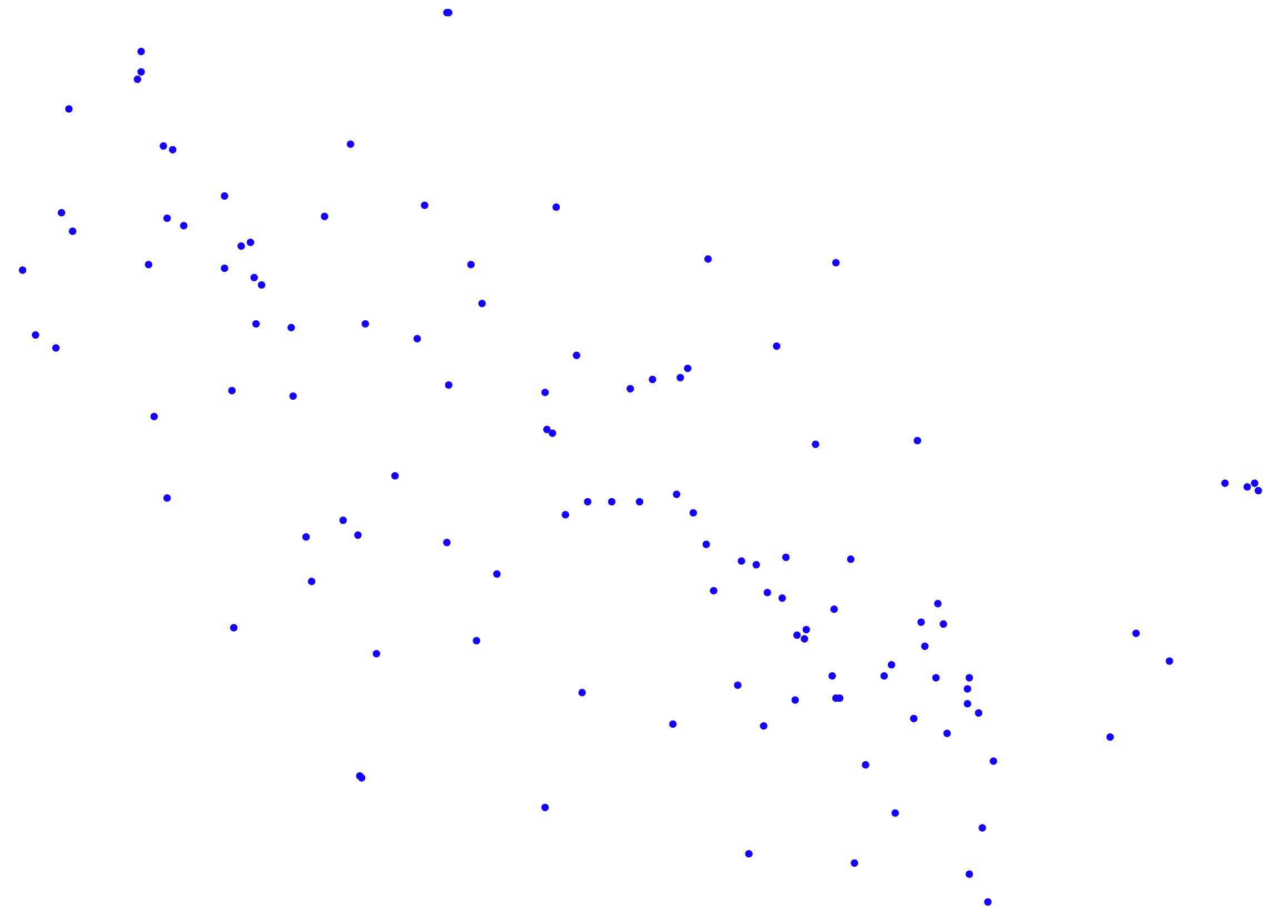

Supplement: Supplementary file 2 — ZIP archive containing VizBin visualization screenshots of the individual bins for the three datasets (37A, 37B, and SRS013705) originally reported in [ 16 ]. [file 40168_2014_66_MOESM2_ESM.zip › 37A_37B_SRS013705/37B/37B.out.002.png]

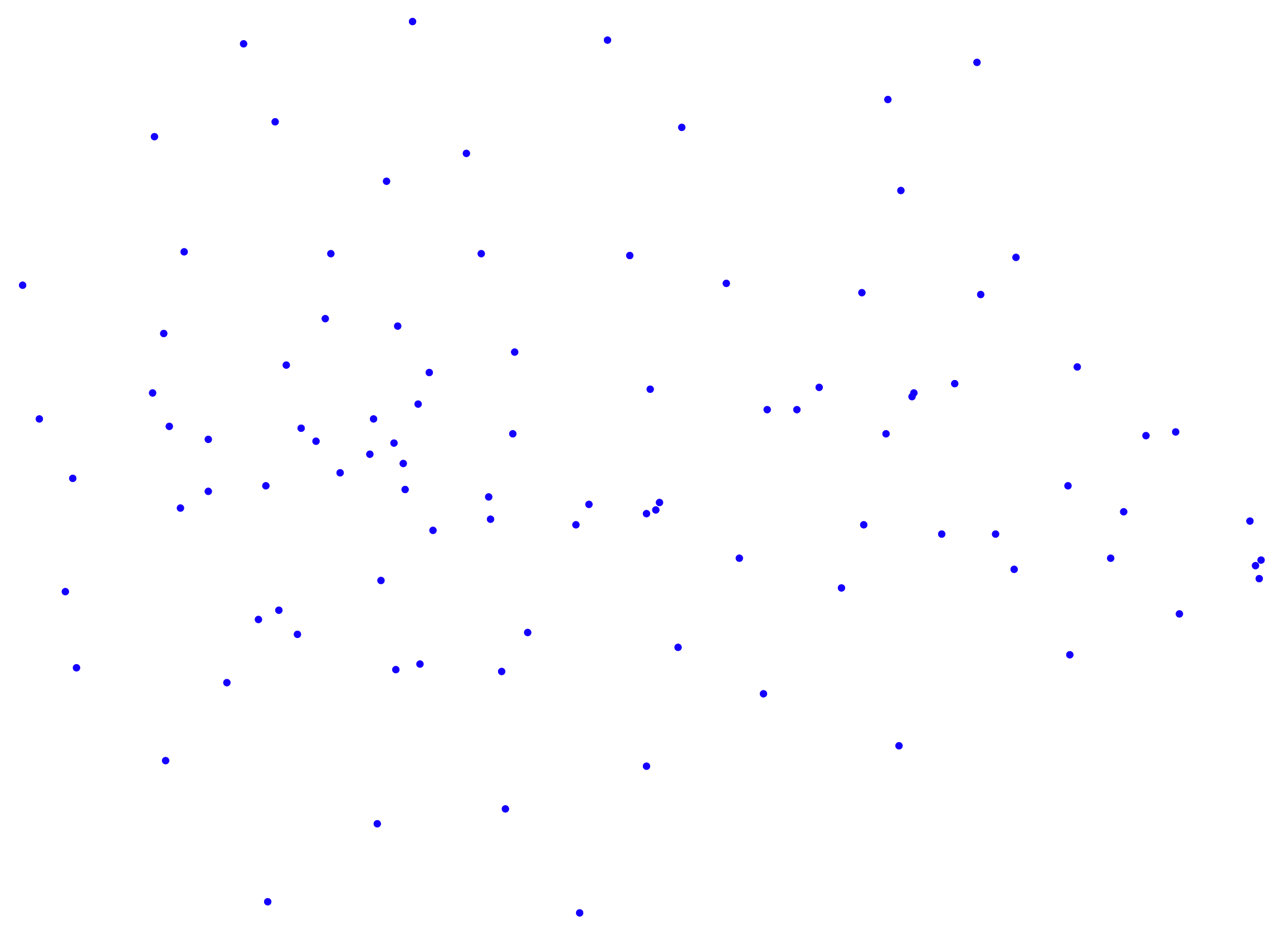

Supplement: Supplementary file 2 — ZIP archive containing VizBin visualization screenshots of the individual bins for the three datasets (37A, 37B, and SRS013705) originally reported in [ 16 ]. [file 40168_2014_66_MOESM2_ESM.zip › 37A_37B_SRS013705/37B/37B.out.003.png]

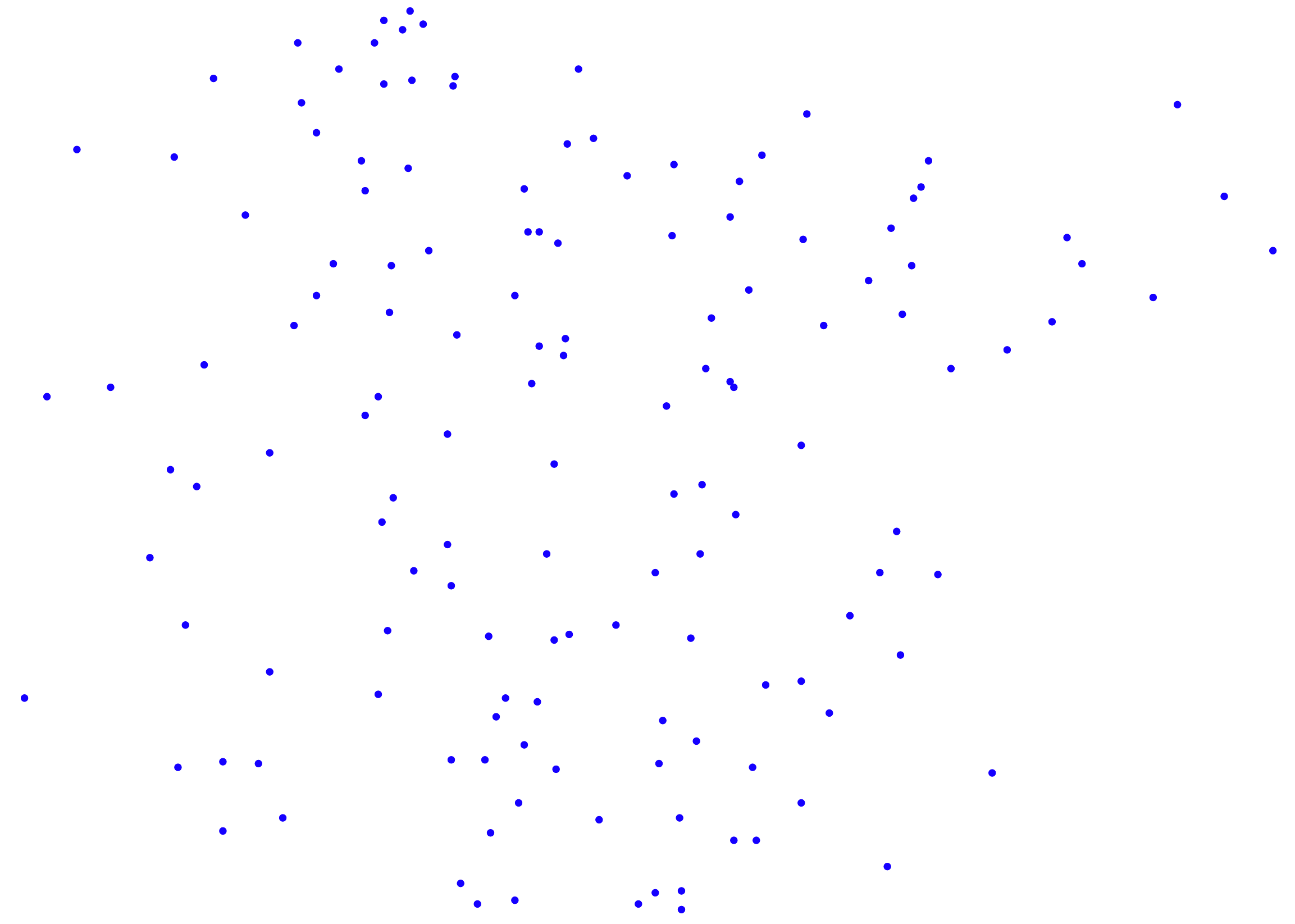

Supplement: Supplementary file 2 — ZIP archive containing VizBin visualization screenshots of the individual bins for the three datasets (37A, 37B, and SRS013705) originally reported in [ 16 ]. [file 40168_2014_66_MOESM2_ESM.zip › 37A_37B_SRS013705/37B/37B.out.004.png]

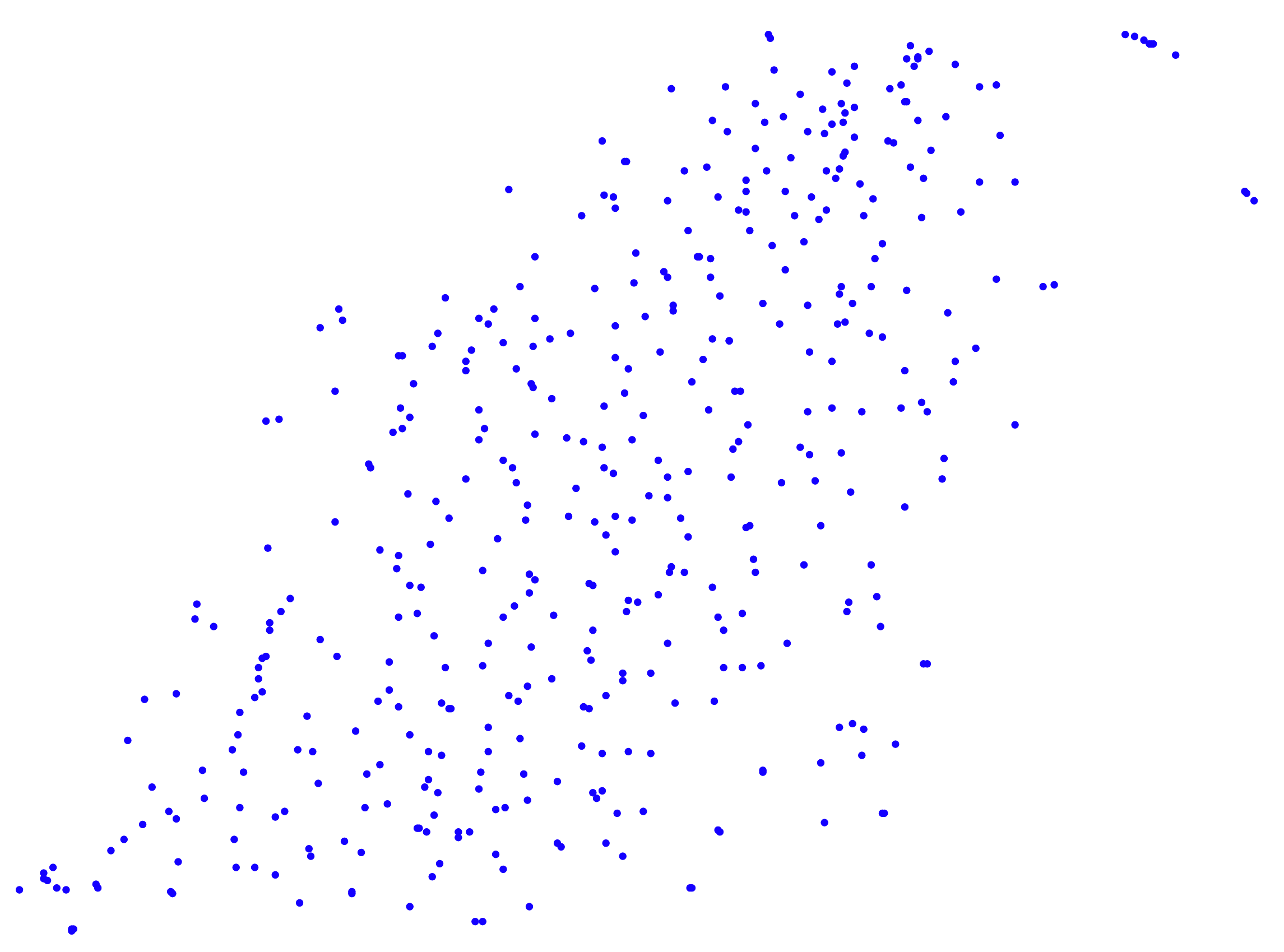

Supplement: Supplementary file 2 — ZIP archive containing VizBin visualization screenshots of the individual bins for the three datasets (37A, 37B, and SRS013705) originally reported in [ 16 ]. [file 40168_2014_66_MOESM2_ESM.zip › 37A_37B_SRS013705/37B/37B.out.005.png]

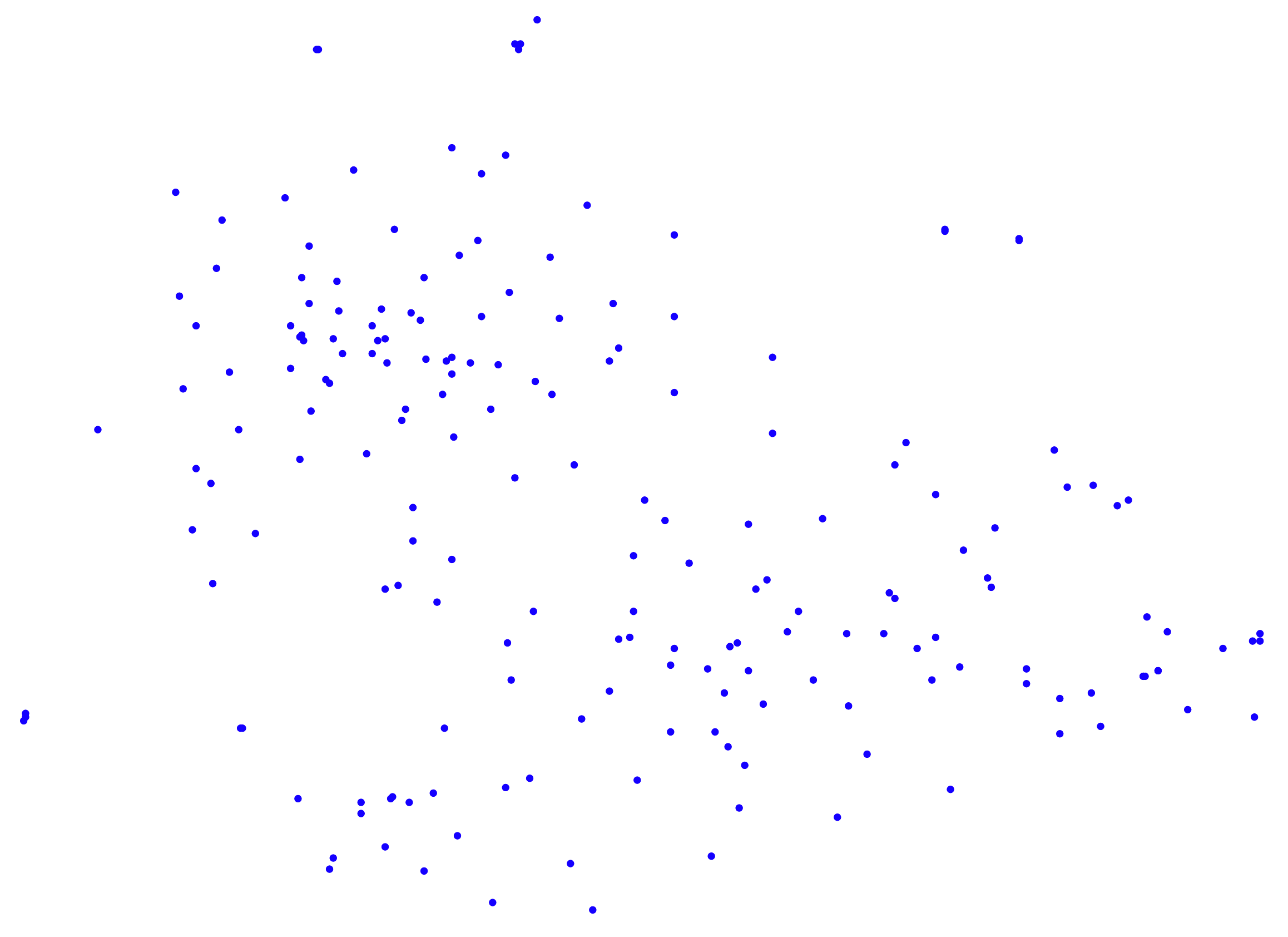

Supplement: Supplementary file 2 — ZIP archive containing VizBin visualization screenshots of the individual bins for the three datasets (37A, 37B, and SRS013705) originally reported in [ 16 ]. [file 40168_2014_66_MOESM2_ESM.zip › 37A_37B_SRS013705/37B/37B.out.006.png]

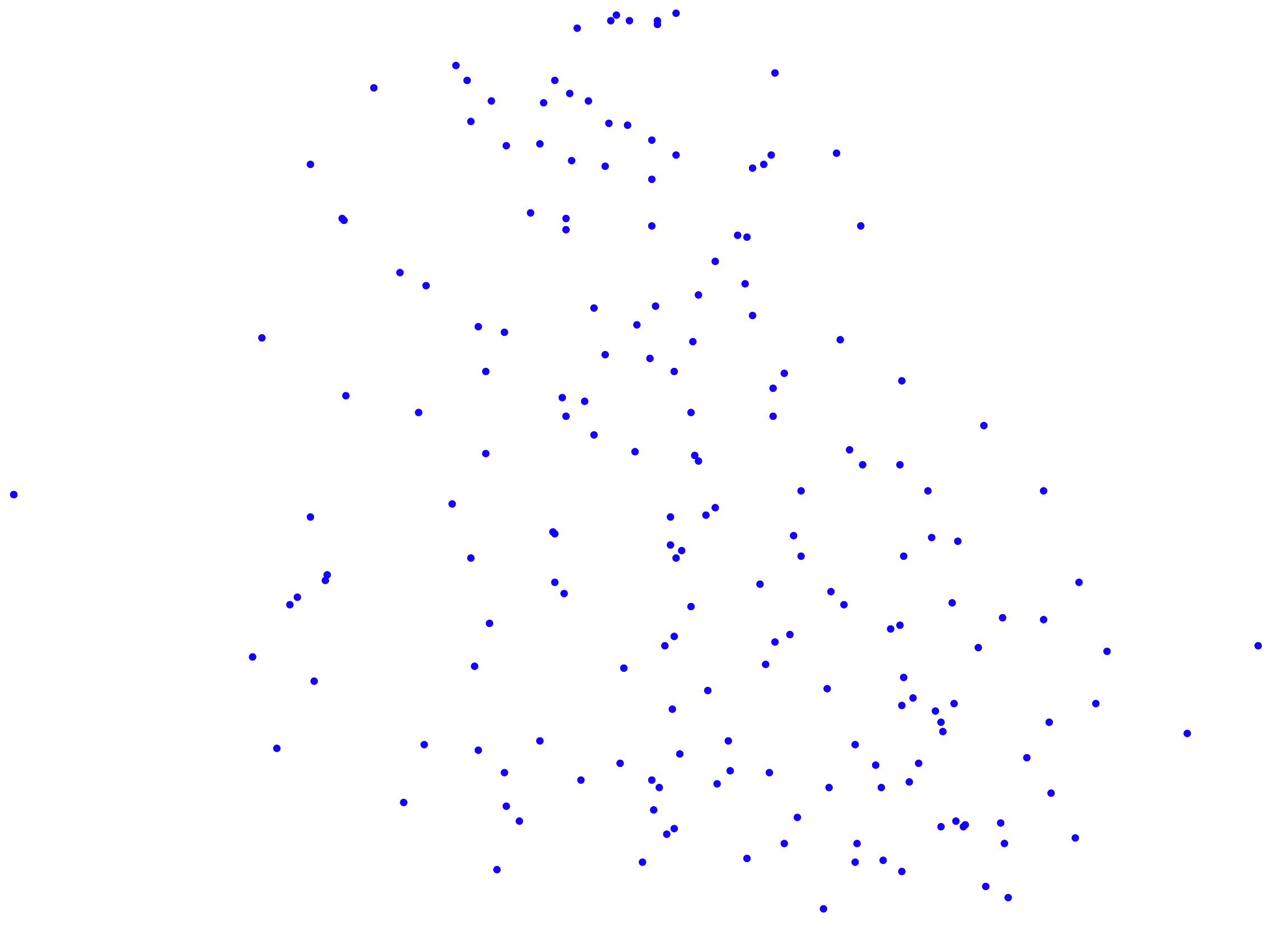

Supplement: Supplementary file 2 — ZIP archive containing VizBin visualization screenshots of the individual bins for the three datasets (37A, 37B, and SRS013705) originally reported in [ 16 ]. [file 40168_2014_66_MOESM2_ESM.zip › 37A_37B_SRS013705/37B/37B.out.007.png]

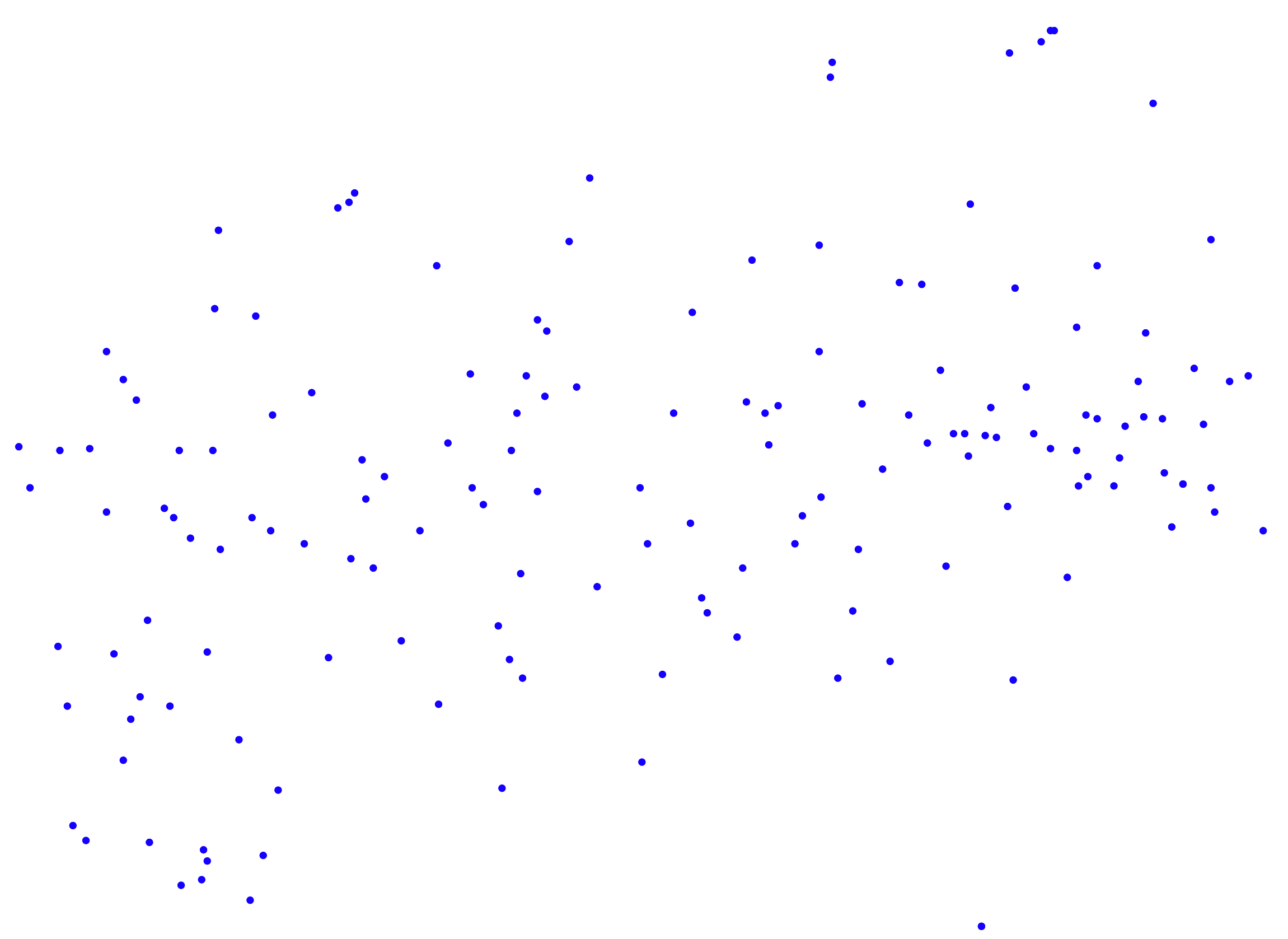

Supplement: Supplementary file 2 — ZIP archive containing VizBin visualization screenshots of the individual bins for the three datasets (37A, 37B, and SRS013705) originally reported in [ 16 ]. [file 40168_2014_66_MOESM2_ESM.zip › 37A_37B_SRS013705/37B/37B.out.008.png]

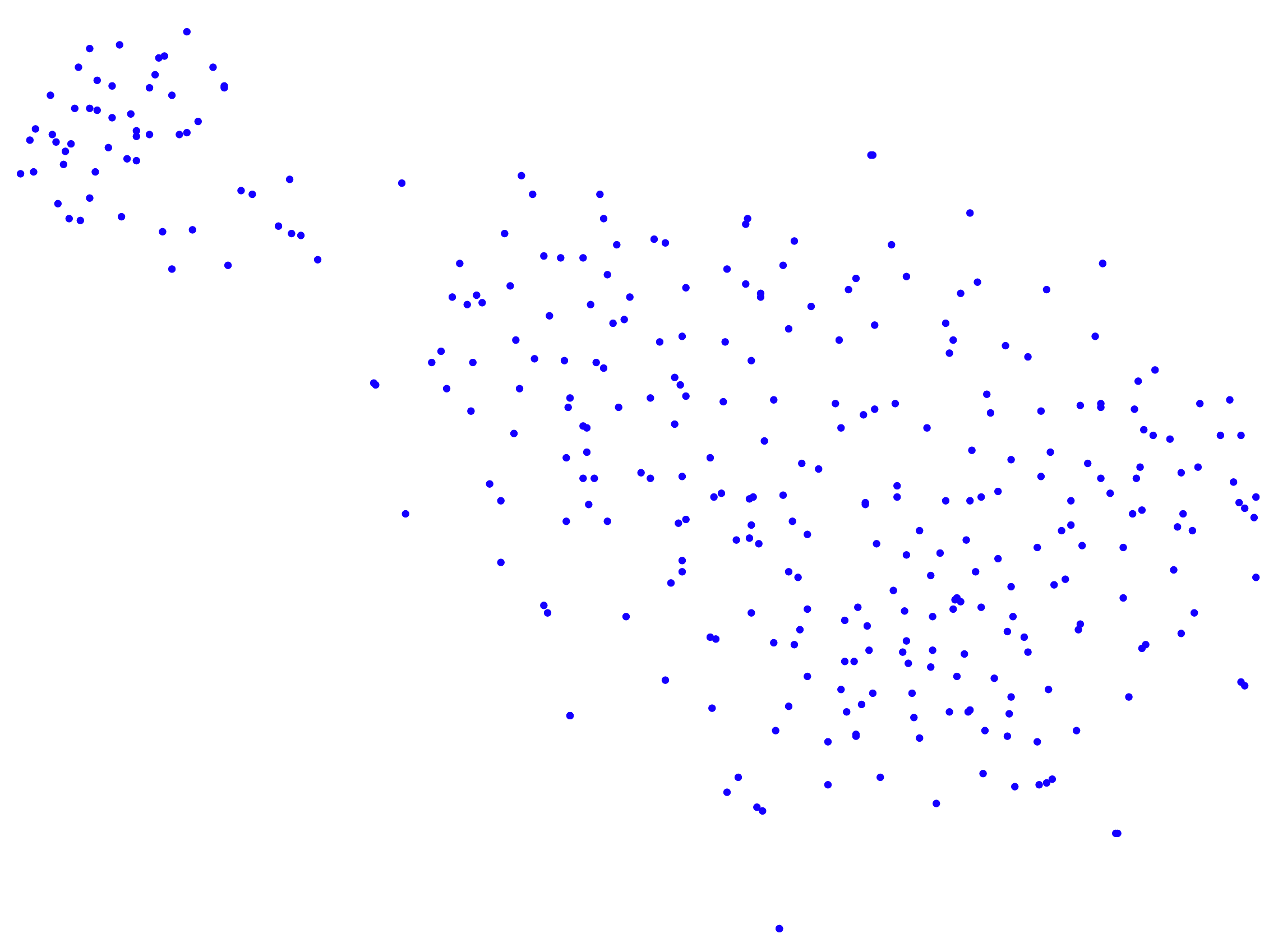

Supplement: Supplementary file 2 — ZIP archive containing VizBin visualization screenshots of the individual bins for the three datasets (37A, 37B, and SRS013705) originally reported in [ 16 ]. [file 40168_2014_66_MOESM2_ESM.zip › 37A_37B_SRS013705/37B/37B.out.009.png]

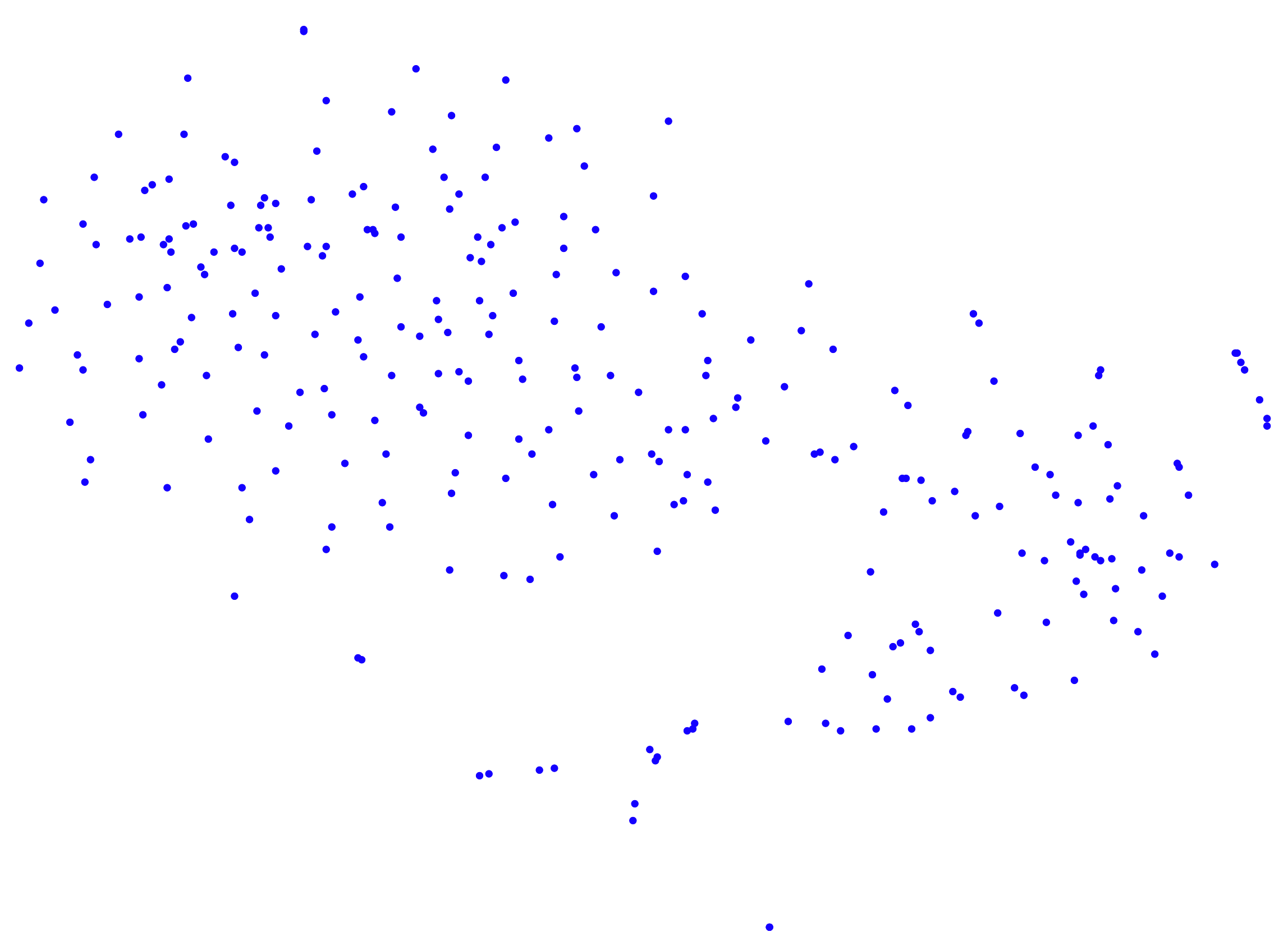

Supplement: Supplementary file 2 — ZIP archive containing VizBin visualization screenshots of the individual bins for the three datasets (37A, 37B, and SRS013705) originally reported in [ 16 ]. [file 40168_2014_66_MOESM2_ESM.zip › 37A_37B_SRS013705/37B/37B.out.010.png]

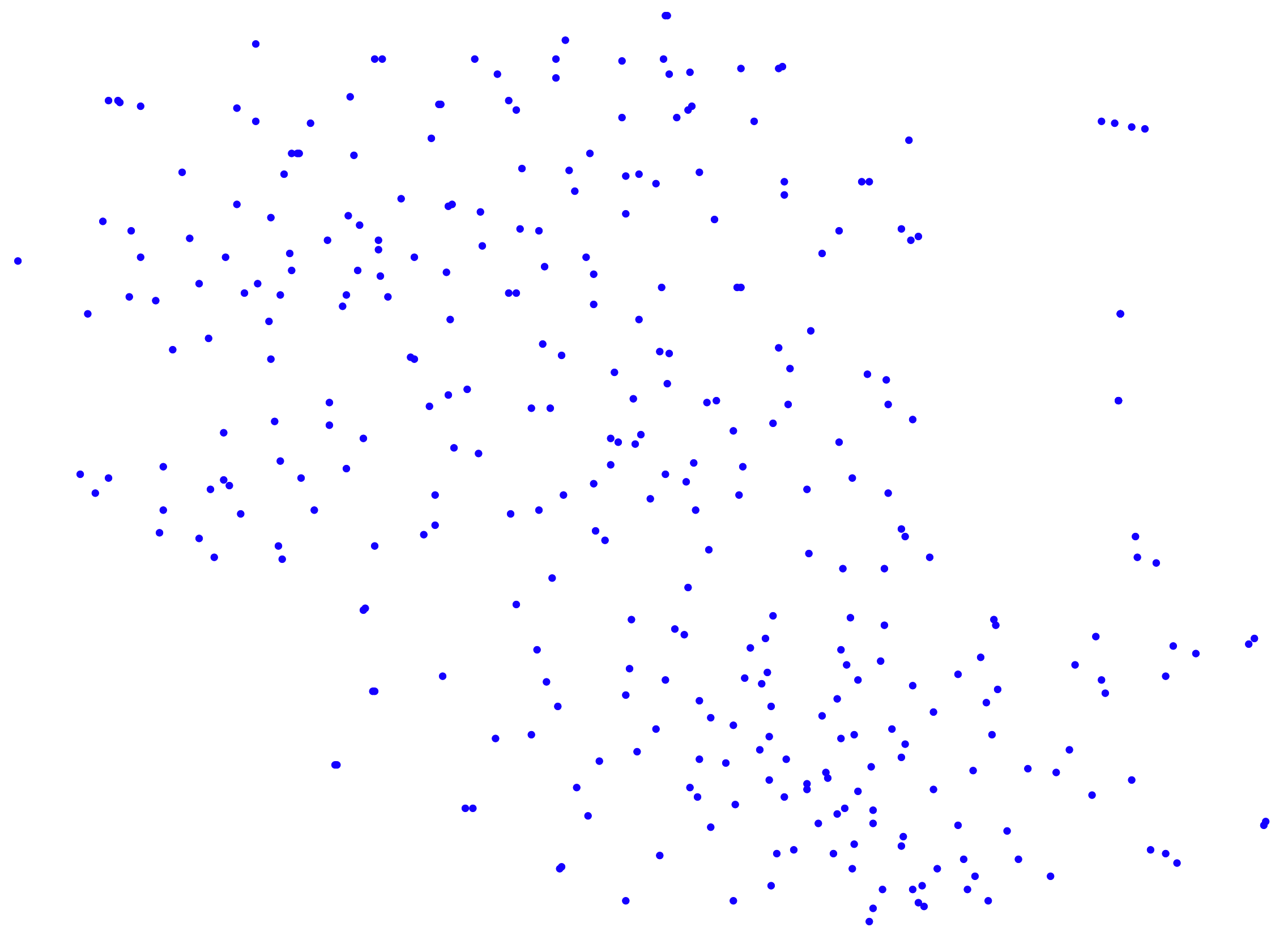

Supplement: Supplementary file 2 — ZIP archive containing VizBin visualization screenshots of the individual bins for the three datasets (37A, 37B, and SRS013705) originally reported in [ 16 ]. [file 40168_2014_66_MOESM2_ESM.zip › 37A_37B_SRS013705/37B/37B.out.011.png]

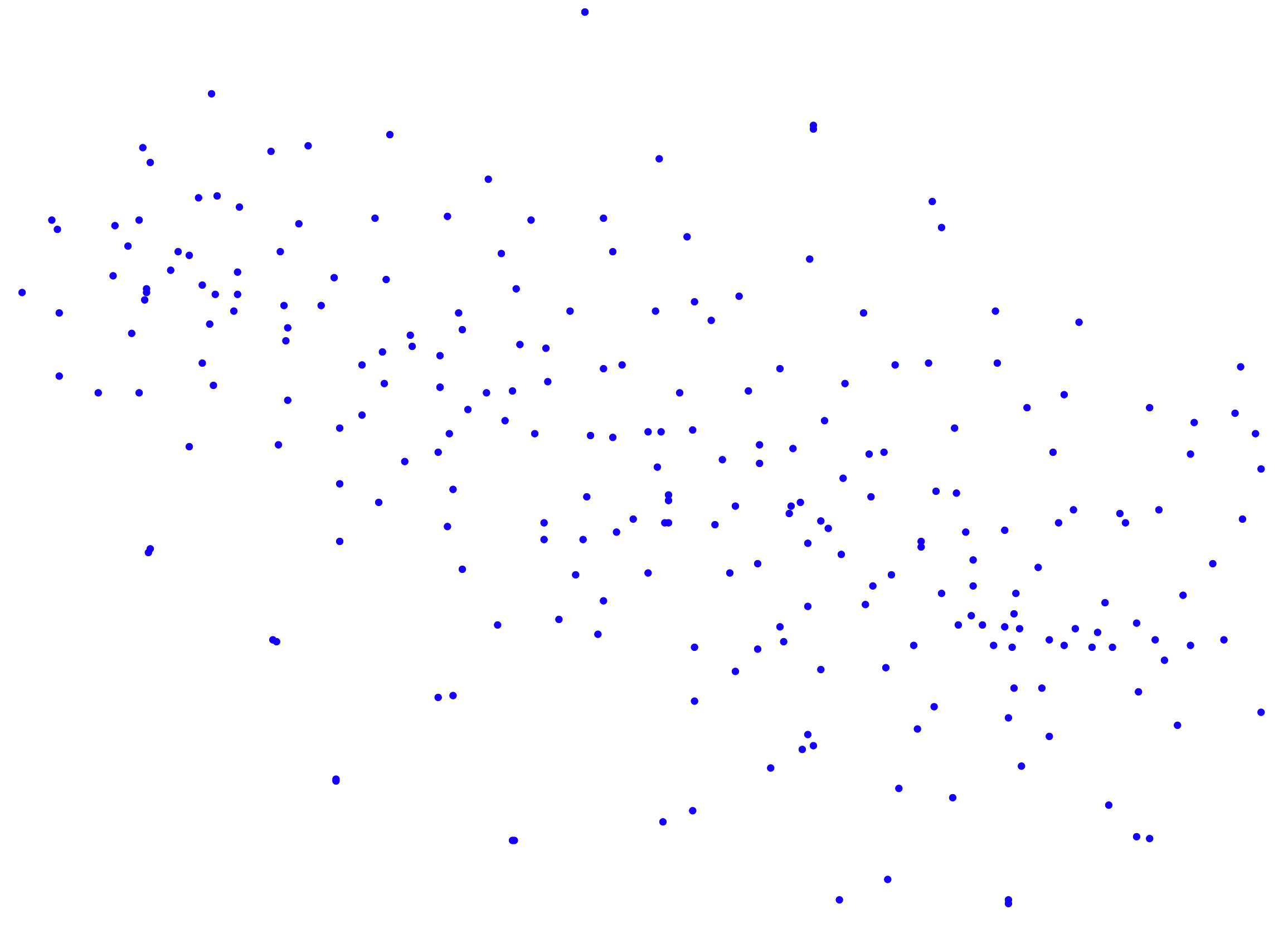

Supplement: Supplementary file 2 — ZIP archive containing VizBin visualization screenshots of the individual bins for the three datasets (37A, 37B, and SRS013705) originally reported in [ 16 ]. [file 40168_2014_66_MOESM2_ESM.zip › 37A_37B_SRS013705/37B/37B.out.012.png]

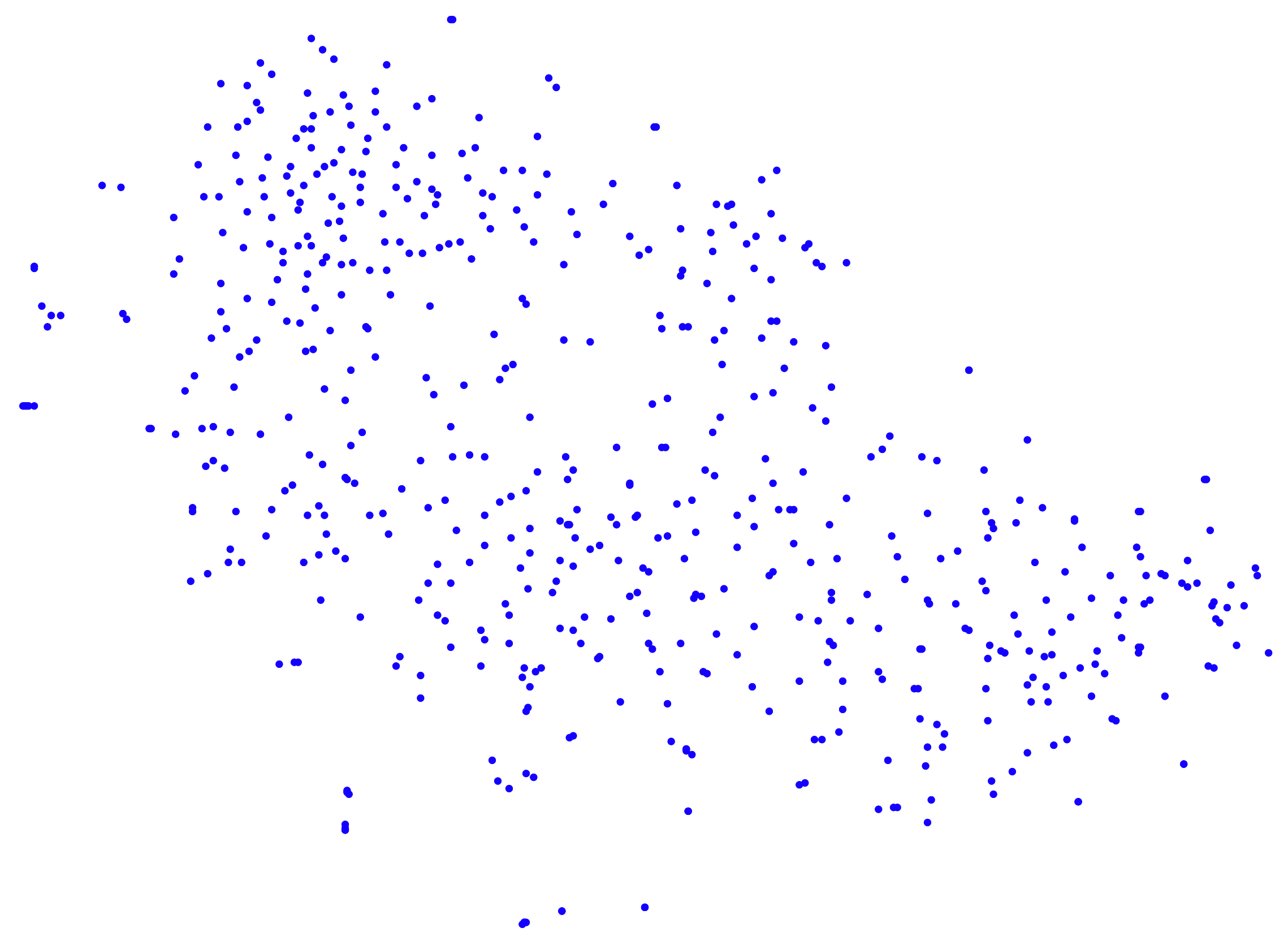

Supplement: Supplementary file 2 — ZIP archive containing VizBin visualization screenshots of the individual bins for the three datasets (37A, 37B, and SRS013705) originally reported in [ 16 ]. [file 40168_2014_66_MOESM2_ESM.zip › 37A_37B_SRS013705/37B/37B.out.013.png]

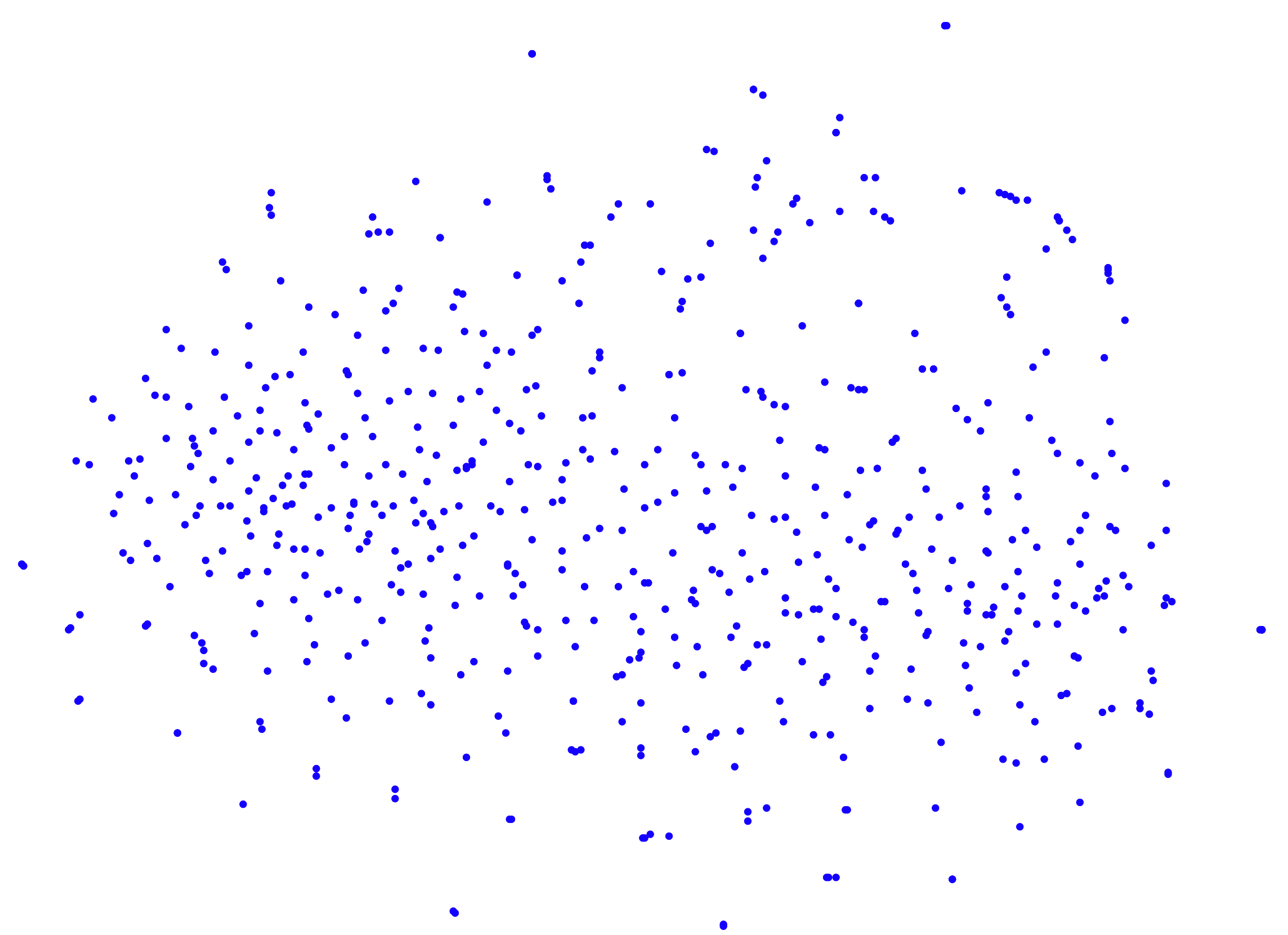

Supplement: Supplementary file 2 — ZIP archive containing VizBin visualization screenshots of the individual bins for the three datasets (37A, 37B, and SRS013705) originally reported in [ 16 ]. [file 40168_2014_66_MOESM2_ESM.zip › 37A_37B_SRS013705/37B/37B.out.014.png]

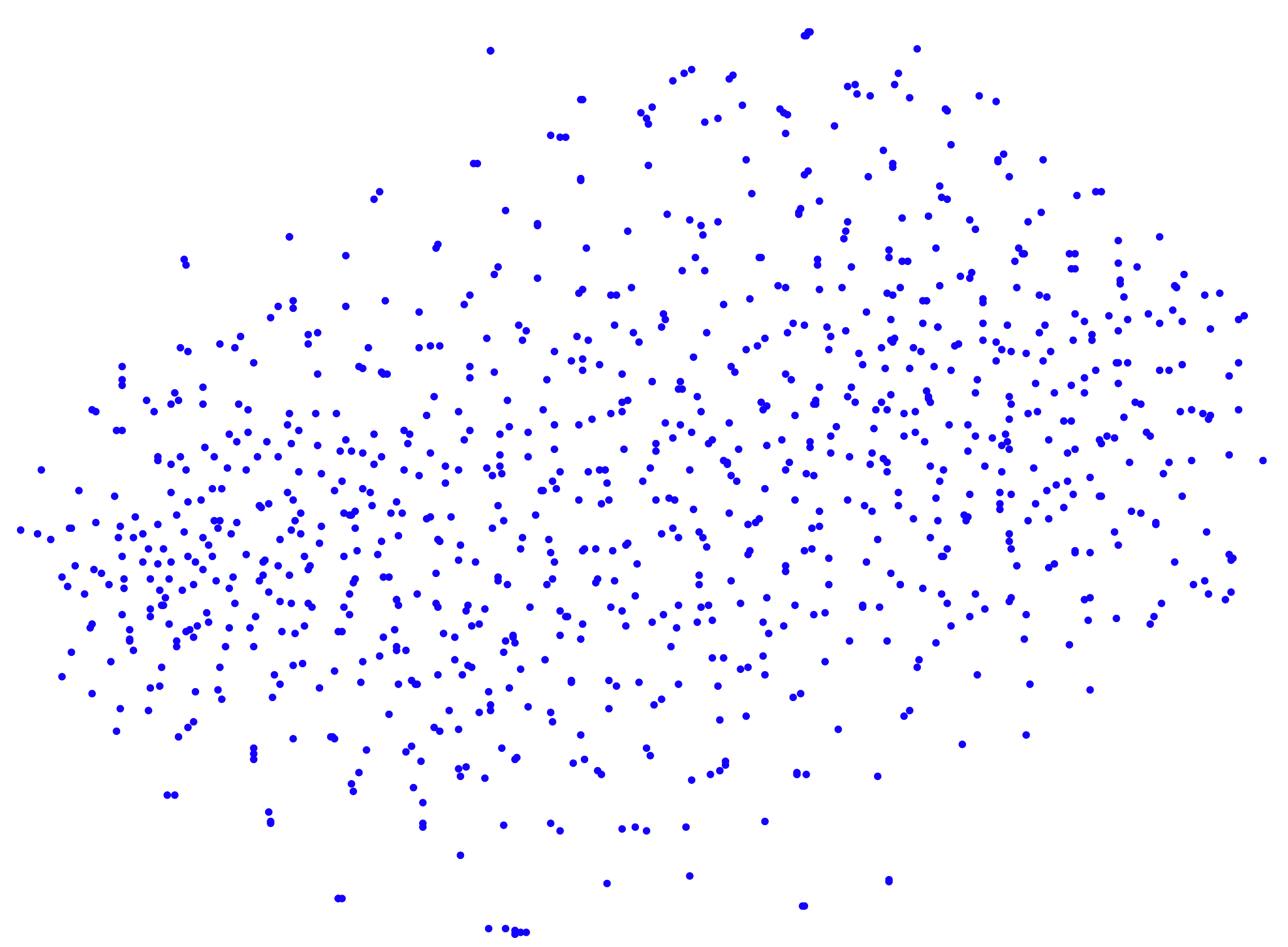

Supplement: Supplementary file 2 — ZIP archive containing VizBin visualization screenshots of the individual bins for the three datasets (37A, 37B, and SRS013705) originally reported in [ 16 ]. [file 40168_2014_66_MOESM2_ESM.zip › 37A_37B_SRS013705/37B/37B.out.015.png]

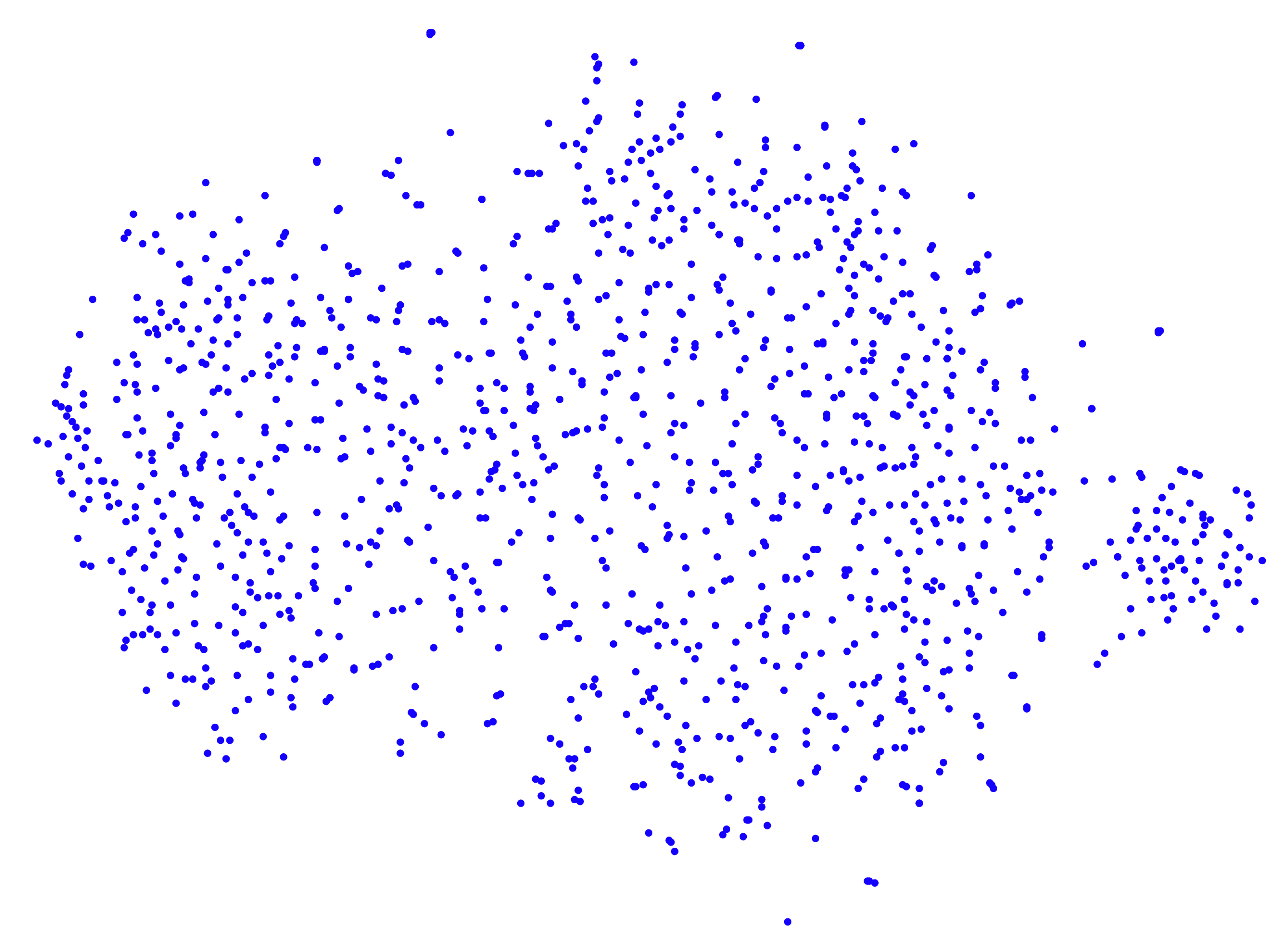

Supplement: Supplementary file 2 — ZIP archive containing VizBin visualization screenshots of the individual bins for the three datasets (37A, 37B, and SRS013705) originally reported in [ 16 ]. [file 40168_2014_66_MOESM2_ESM.zip › 37A_37B_SRS013705/37B/37B.out.016.png]

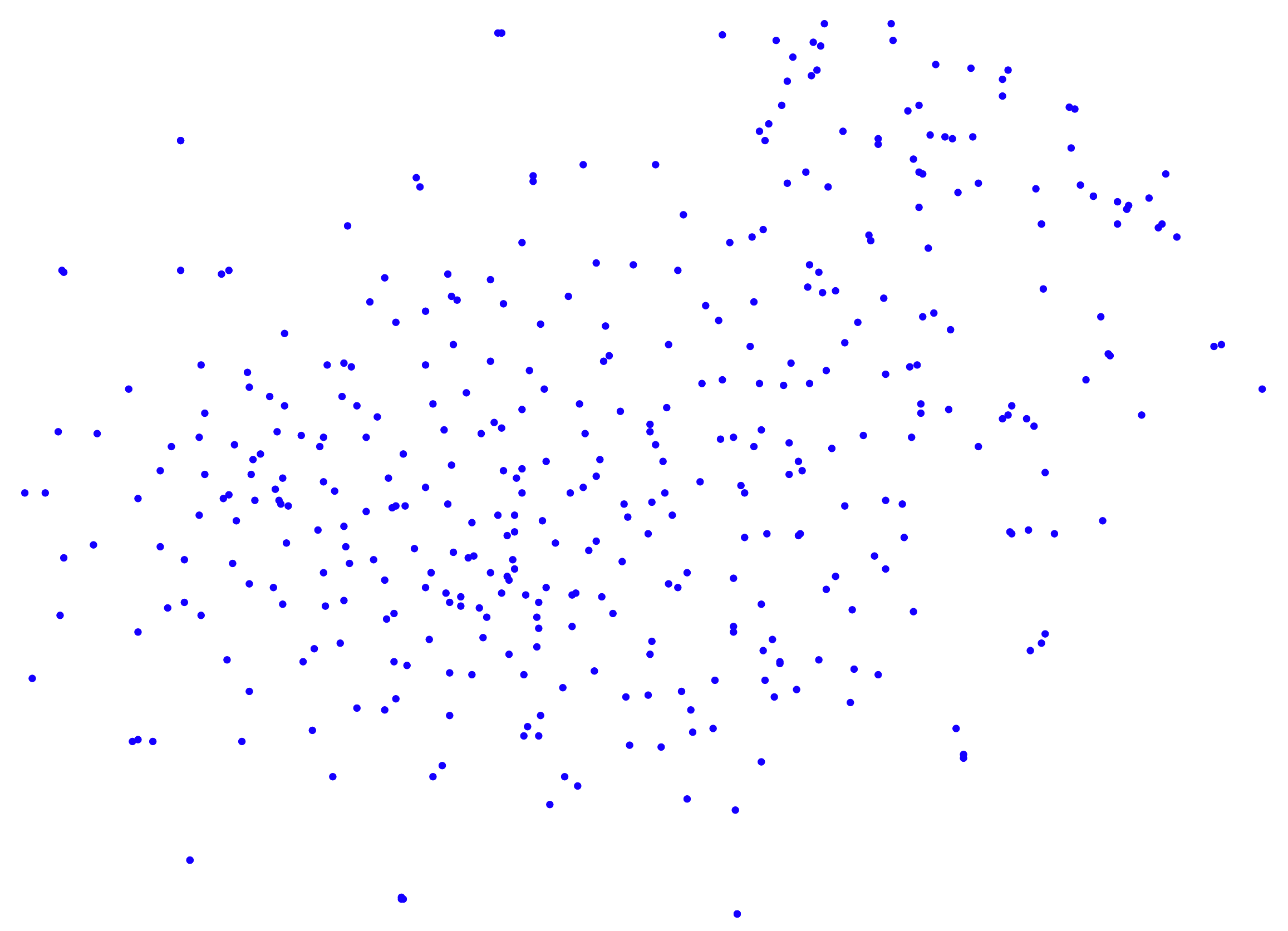

Supplement: Supplementary file 2 — ZIP archive containing VizBin visualization screenshots of the individual bins for the three datasets (37A, 37B, and SRS013705) originally reported in [ 16 ]. [file 40168_2014_66_MOESM2_ESM.zip › 37A_37B_SRS013705/37B/37B.out.017.png]

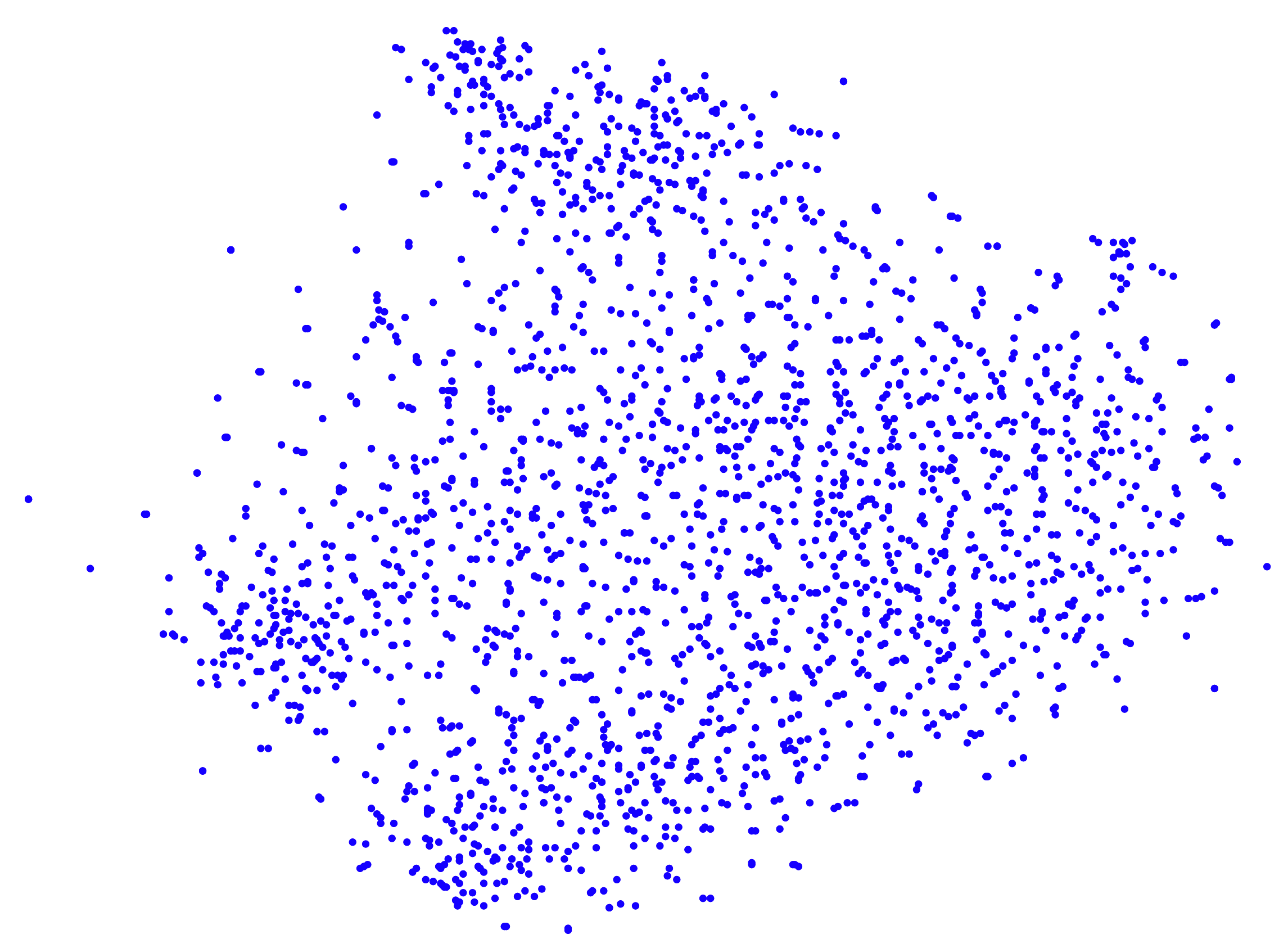

Supplement: Supplementary file 2 — ZIP archive containing VizBin visualization screenshots of the individual bins for the three datasets (37A, 37B, and SRS013705) originally reported in [ 16 ]. [file 40168_2014_66_MOESM2_ESM.zip › 37A_37B_SRS013705/37B/37B.out.018.png]

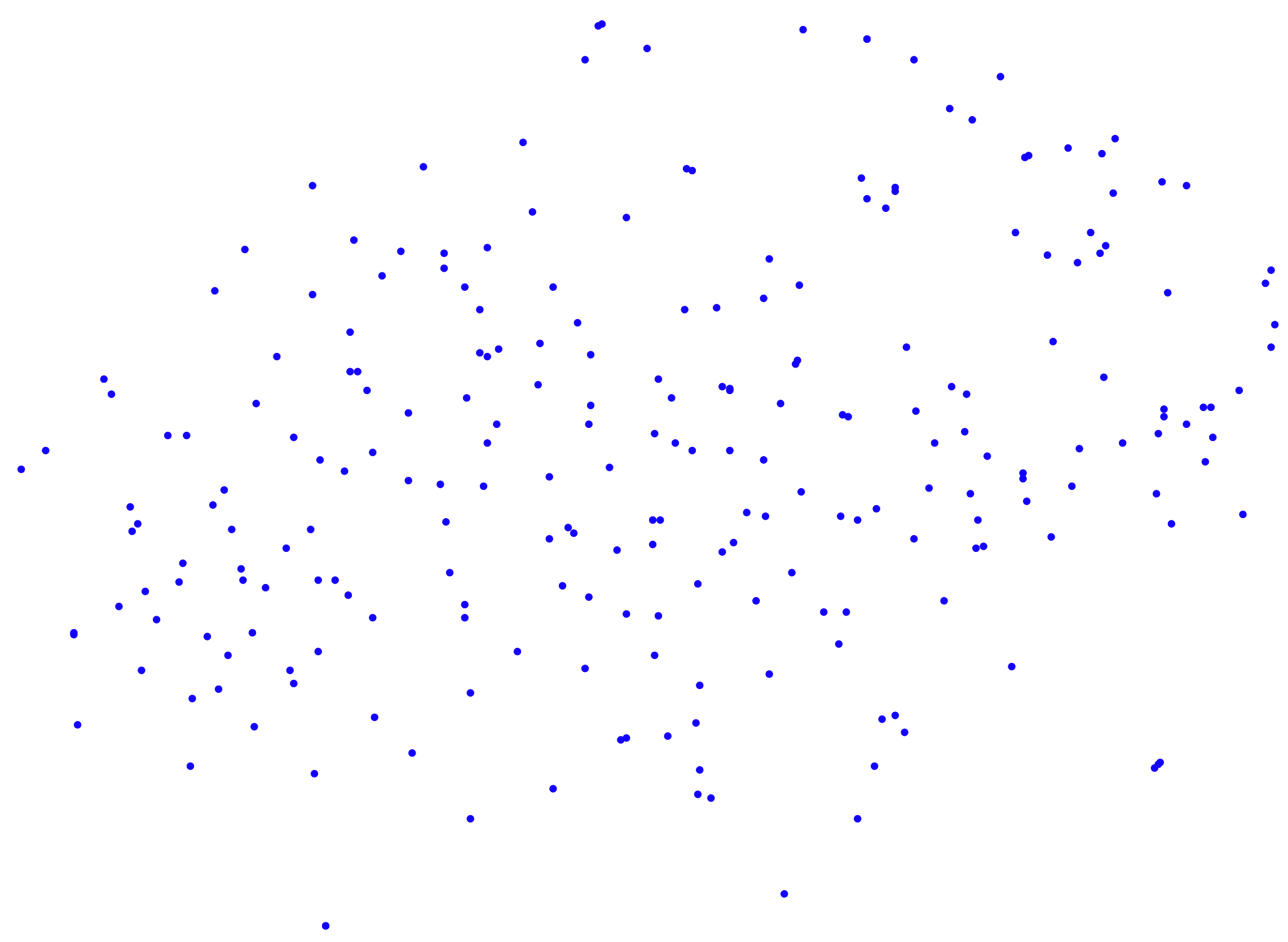

Supplement: Supplementary file 2 — ZIP archive containing VizBin visualization screenshots of the individual bins for the three datasets (37A, 37B, and SRS013705) originally reported in [ 16 ]. [file 40168_2014_66_MOESM2_ESM.zip › 37A_37B_SRS013705/37B/37B.out.019.png]

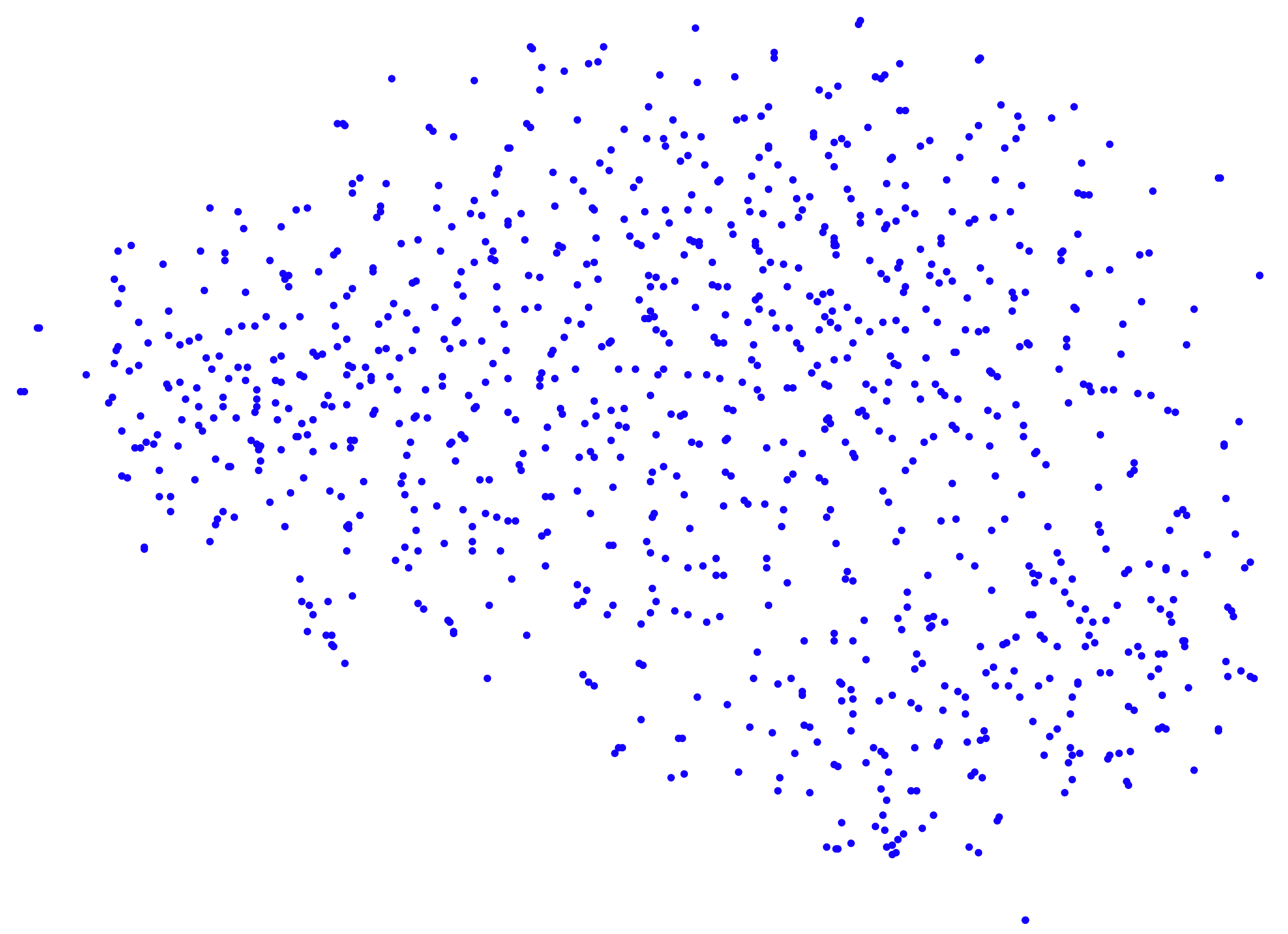

Supplement: Supplementary file 2 — ZIP archive containing VizBin visualization screenshots of the individual bins for the three datasets (37A, 37B, and SRS013705) originally reported in [ 16 ]. [file 40168_2014_66_MOESM2_ESM.zip › 37A_37B_SRS013705/37B/37B.out.020.png]

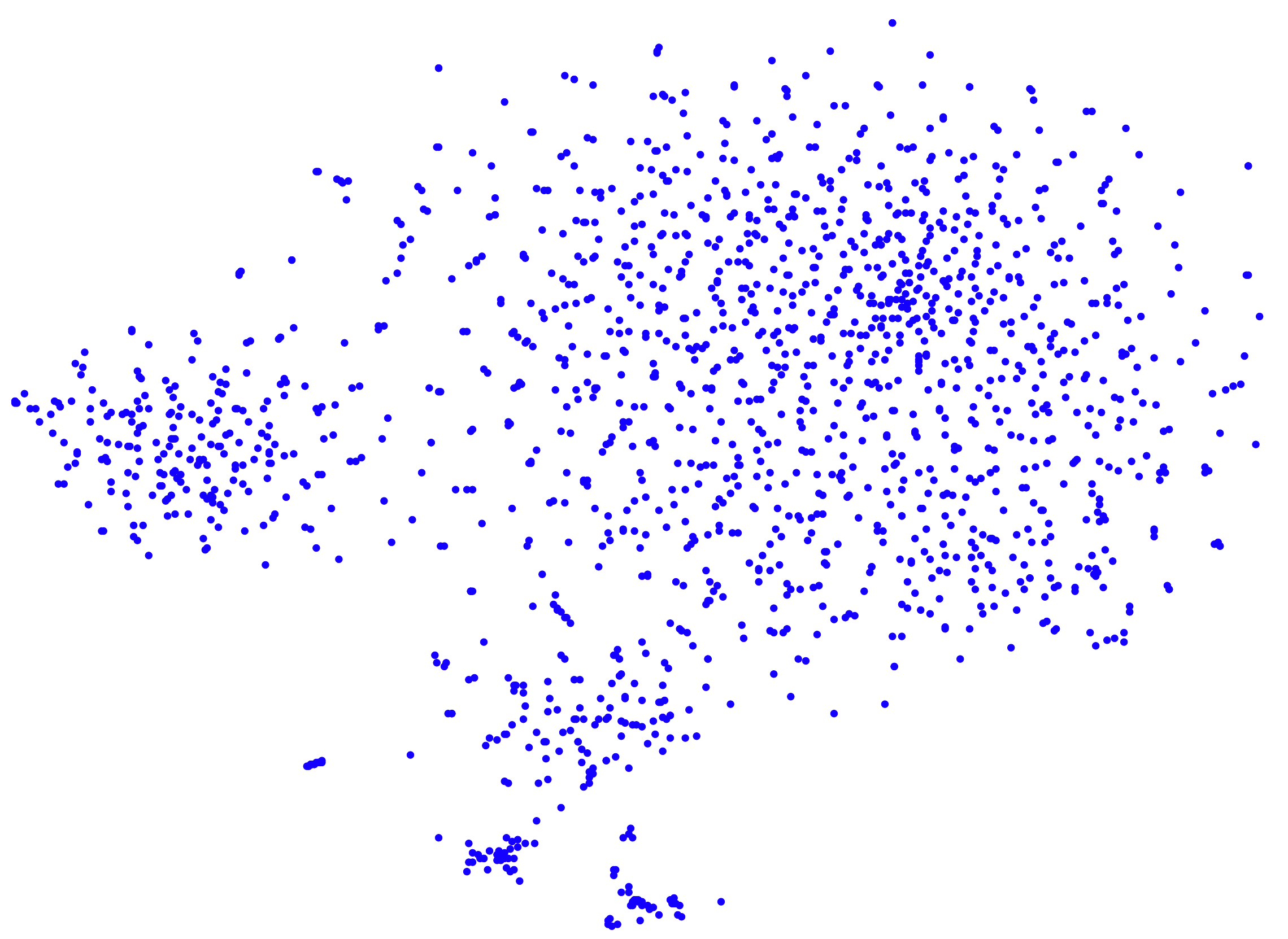

Supplement: Supplementary file 2 — ZIP archive containing VizBin visualization screenshots of the individual bins for the three datasets (37A, 37B, and SRS013705) originally reported in [ 16 ]. [file 40168_2014_66_MOESM2_ESM.zip › 37A_37B_SRS013705/37B/37B.out.021.png]

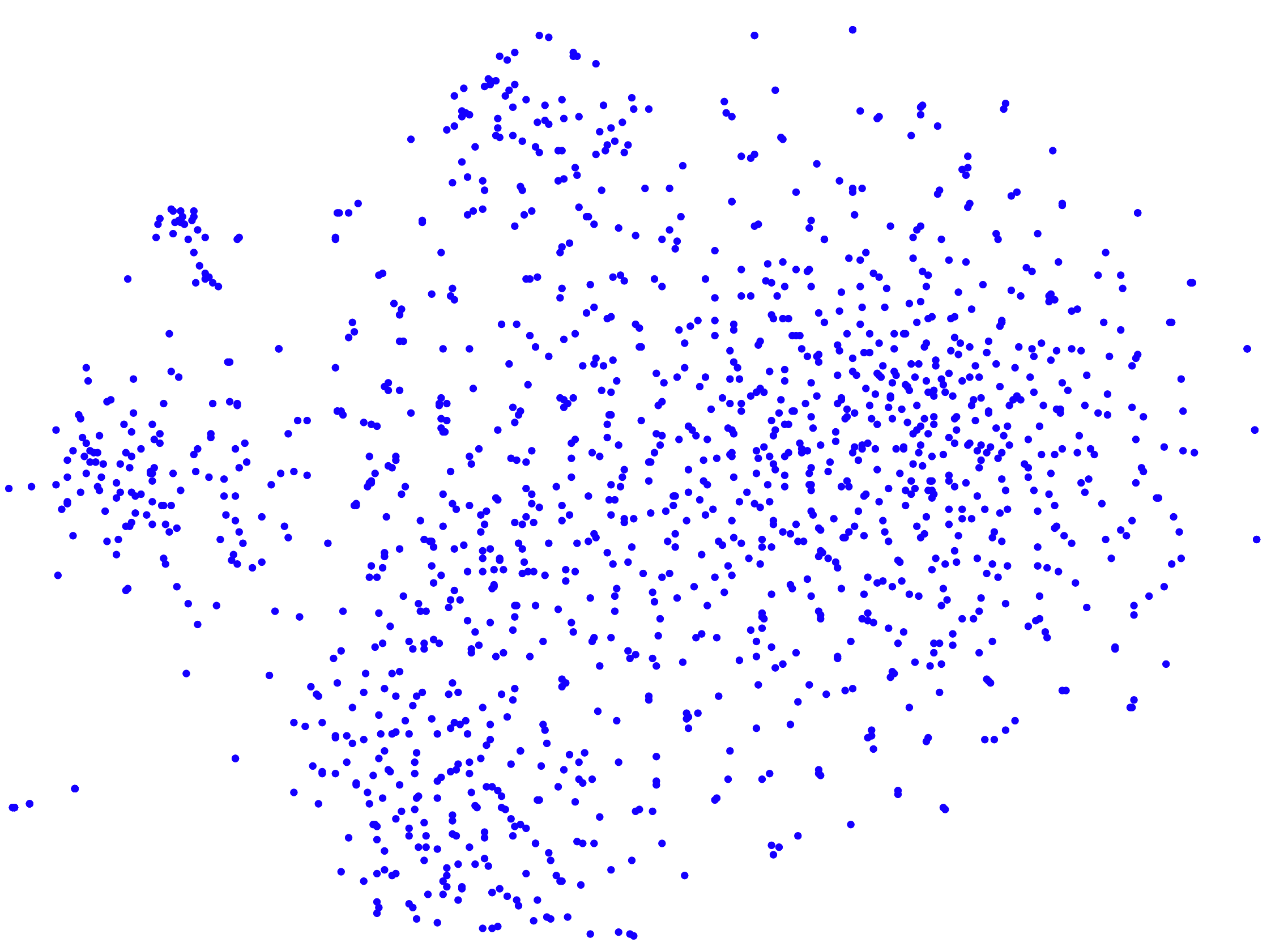

Supplement: Supplementary file 2 — ZIP archive containing VizBin visualization screenshots of the individual bins for the three datasets (37A, 37B, and SRS013705) originally reported in [ 16 ]. [file 40168_2014_66_MOESM2_ESM.zip › 37A_37B_SRS013705/37B/37B.out.022.png]

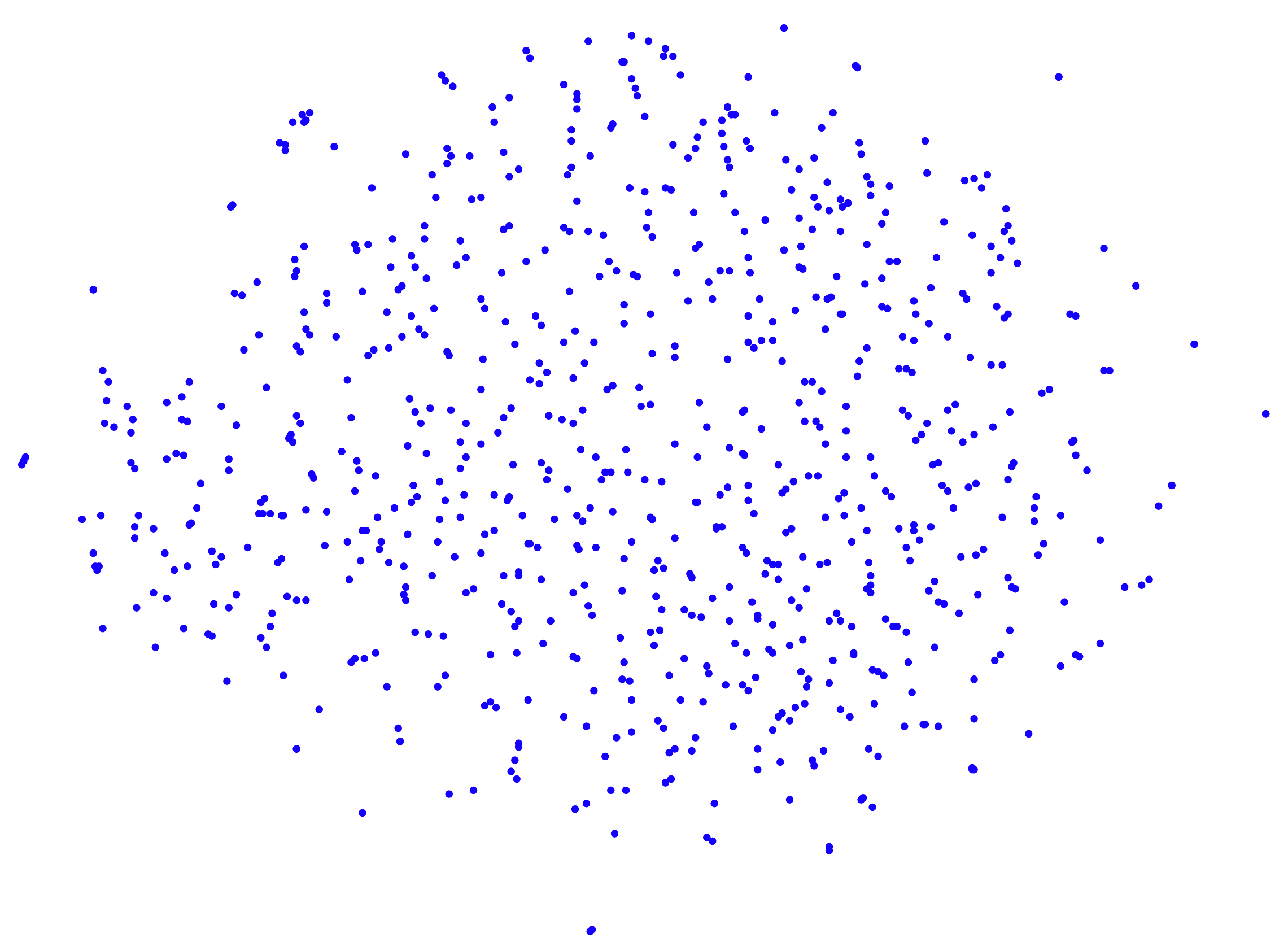

Supplement: Supplementary file 2 — ZIP archive containing VizBin visualization screenshots of the individual bins for the three datasets (37A, 37B, and SRS013705) originally reported in [ 16 ]. [file 40168_2014_66_MOESM2_ESM.zip › 37A_37B_SRS013705/37B/37B.out.023.png]

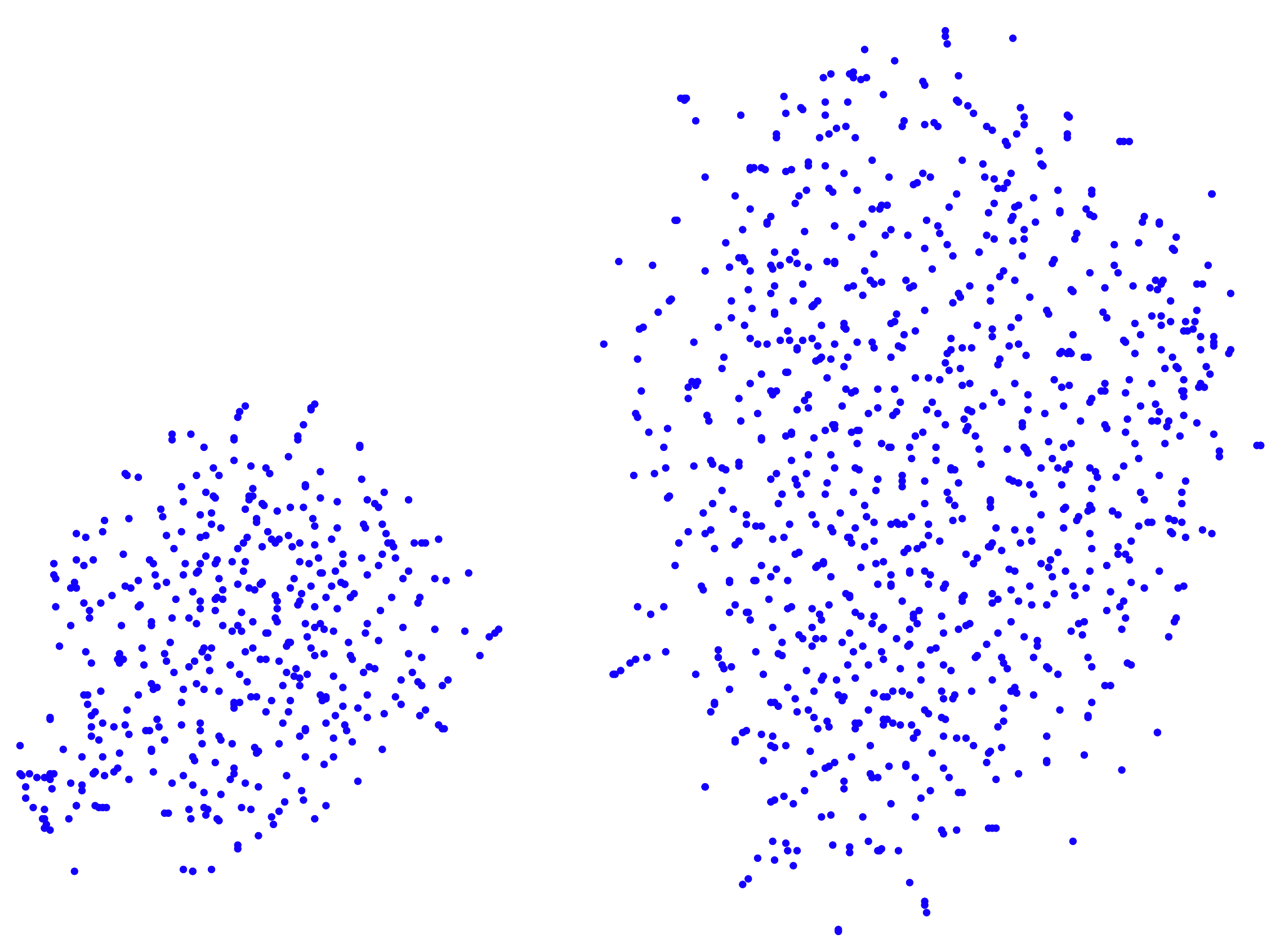

Supplement: Supplementary file 2 — ZIP archive containing VizBin visualization screenshots of the individual bins for the three datasets (37A, 37B, and SRS013705) originally reported in [ 16 ]. [file 40168_2014_66_MOESM2_ESM.zip › 37A_37B_SRS013705/37B/37B.out.024.png]

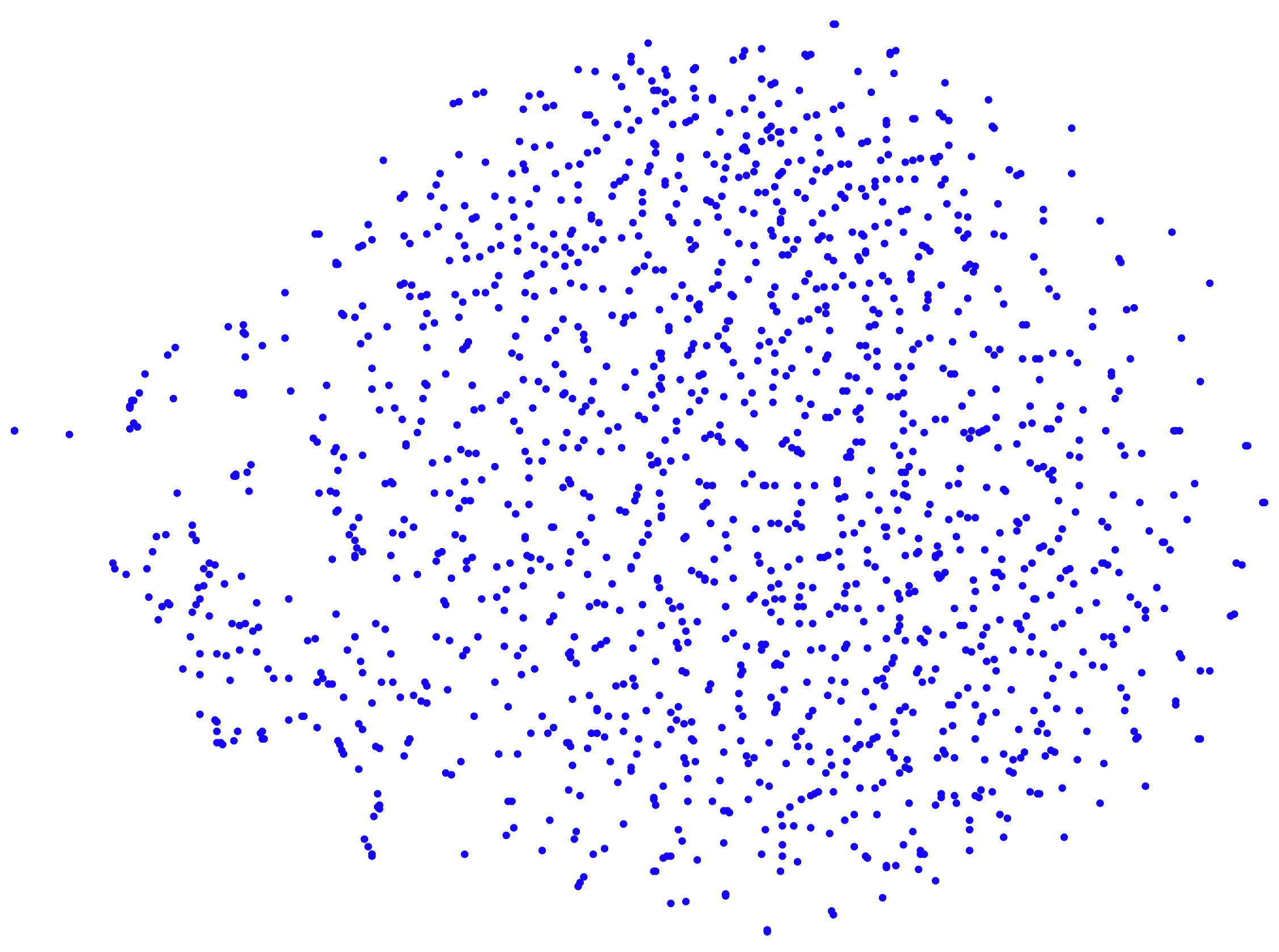

Supplement: Supplementary file 2 — ZIP archive containing VizBin visualization screenshots of the individual bins for the three datasets (37A, 37B, and SRS013705) originally reported in [ 16 ]. [file 40168_2014_66_MOESM2_ESM.zip › 37A_37B_SRS013705/37B/37B.out.025.png]

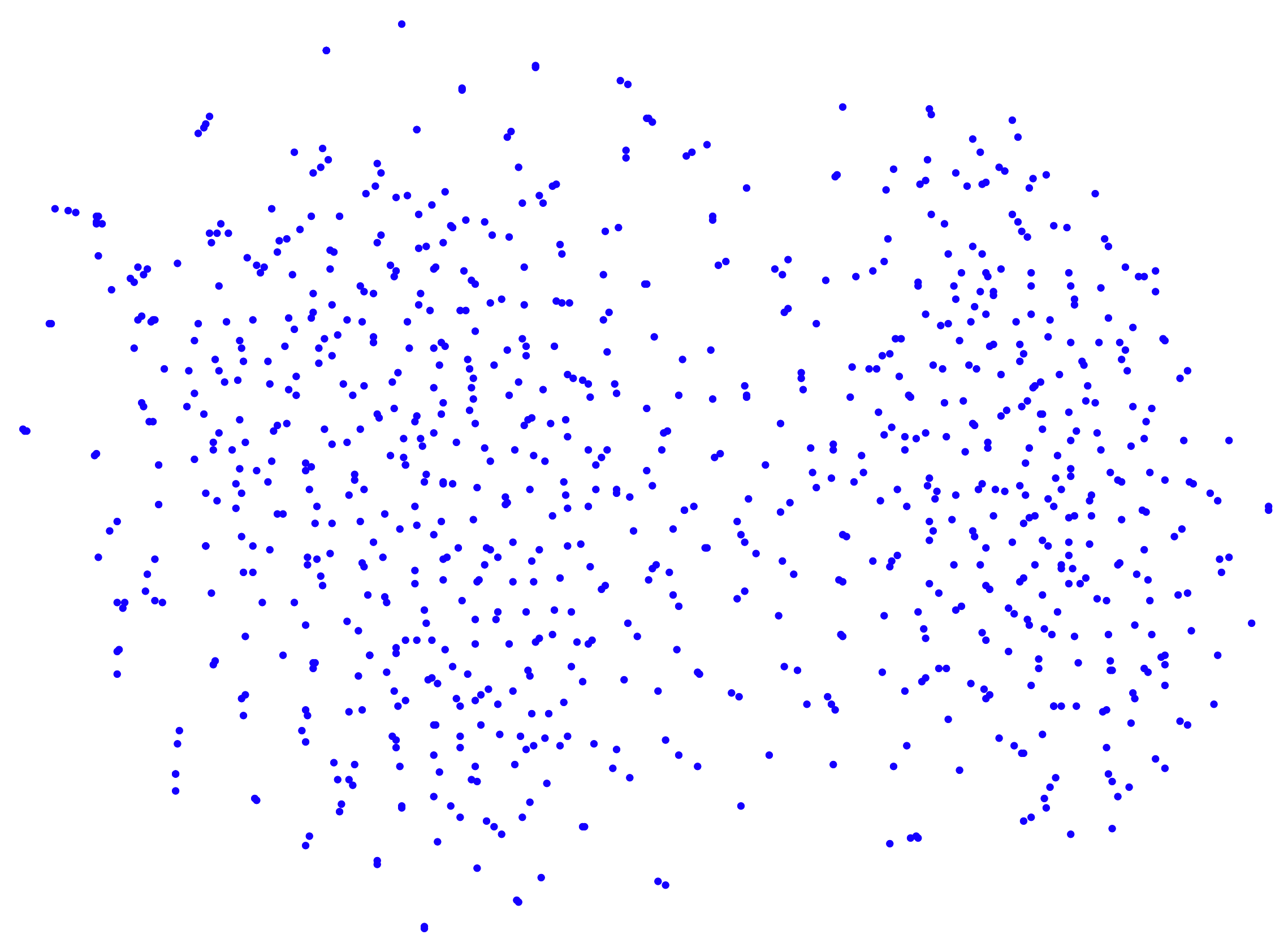

Supplement: Supplementary file 2 — ZIP archive containing VizBin visualization screenshots of the individual bins for the three datasets (37A, 37B, and SRS013705) originally reported in [ 16 ]. [file 40168_2014_66_MOESM2_ESM.zip › 37A_37B_SRS013705/37B/37B.out.026.png]

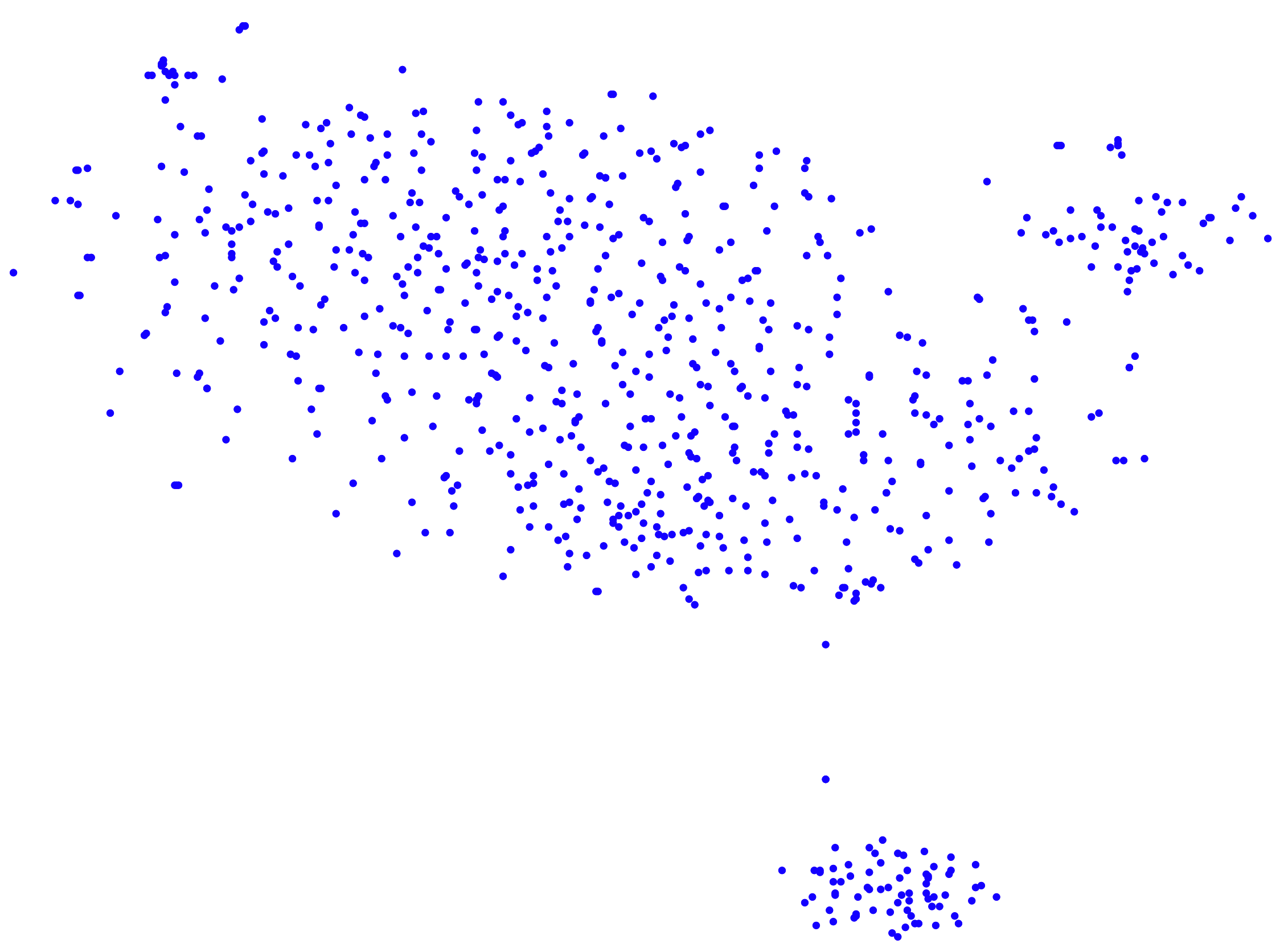

Supplement: Supplementary file 2 — ZIP archive containing VizBin visualization screenshots of the individual bins for the three datasets (37A, 37B, and SRS013705) originally reported in [ 16 ]. [file 40168_2014_66_MOESM2_ESM.zip › 37A_37B_SRS013705/SRS013705/SRS13705.out.001.png]

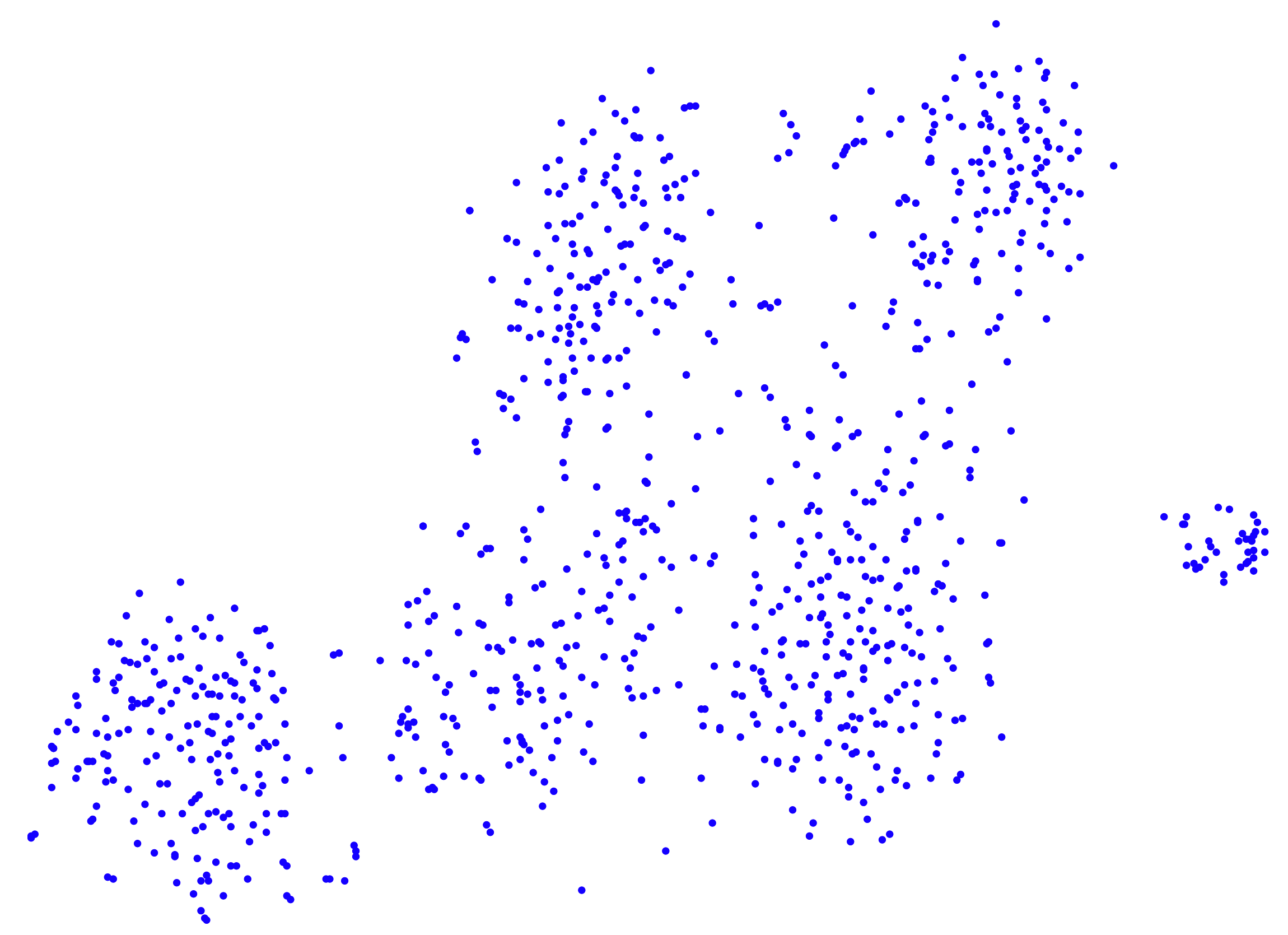

Supplement: Supplementary file 2 — ZIP archive containing VizBin visualization screenshots of the individual bins for the three datasets (37A, 37B, and SRS013705) originally reported in [ 16 ]. [file 40168_2014_66_MOESM2_ESM.zip › 37A_37B_SRS013705/SRS013705/SRS13705.out.002.png]

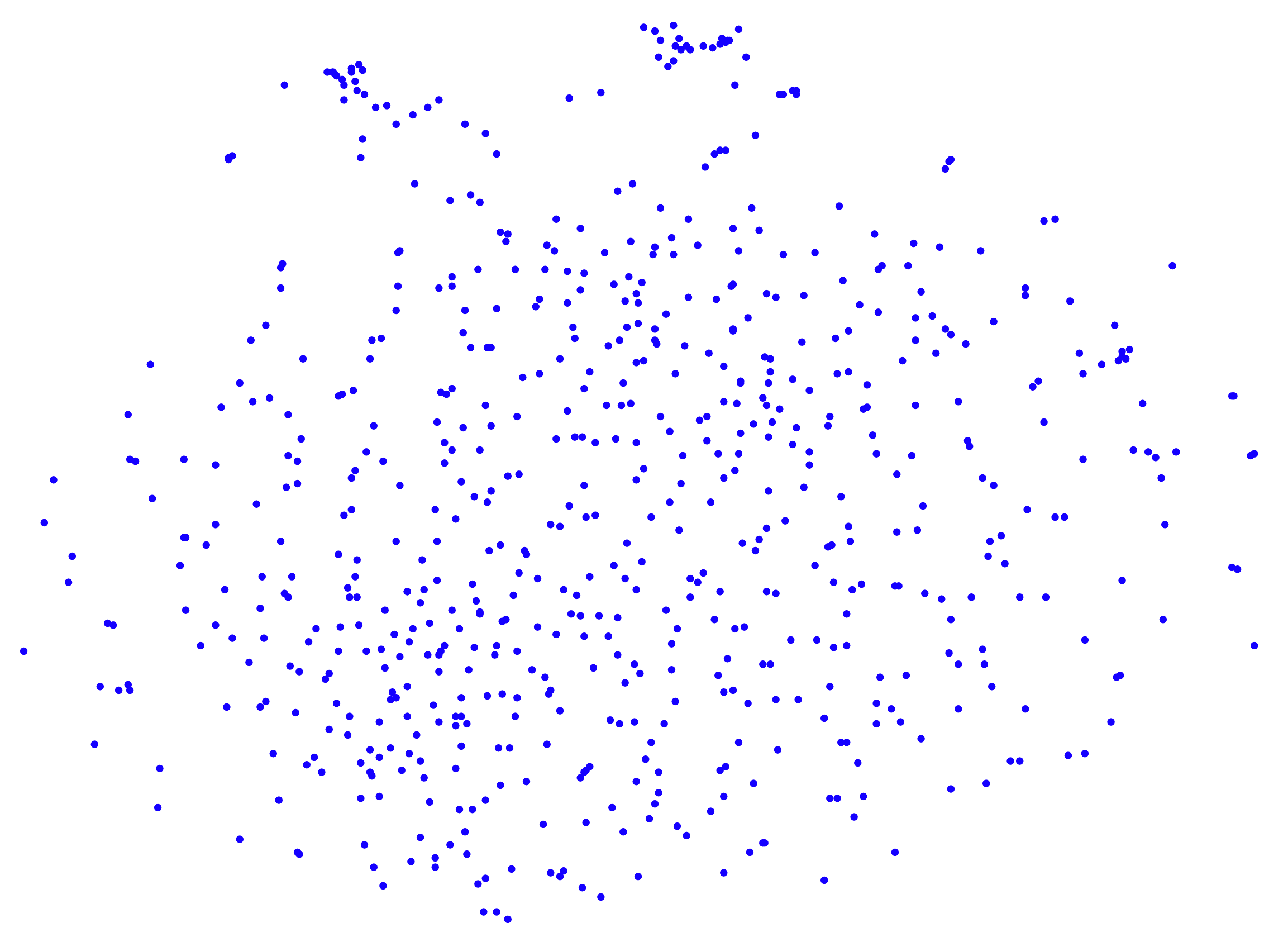

Supplement: Supplementary file 2 — ZIP archive containing VizBin visualization screenshots of the individual bins for the three datasets (37A, 37B, and SRS013705) originally reported in [ 16 ]. [file 40168_2014_66_MOESM2_ESM.zip › 37A_37B_SRS013705/SRS013705/SRS13705.out.003.png]

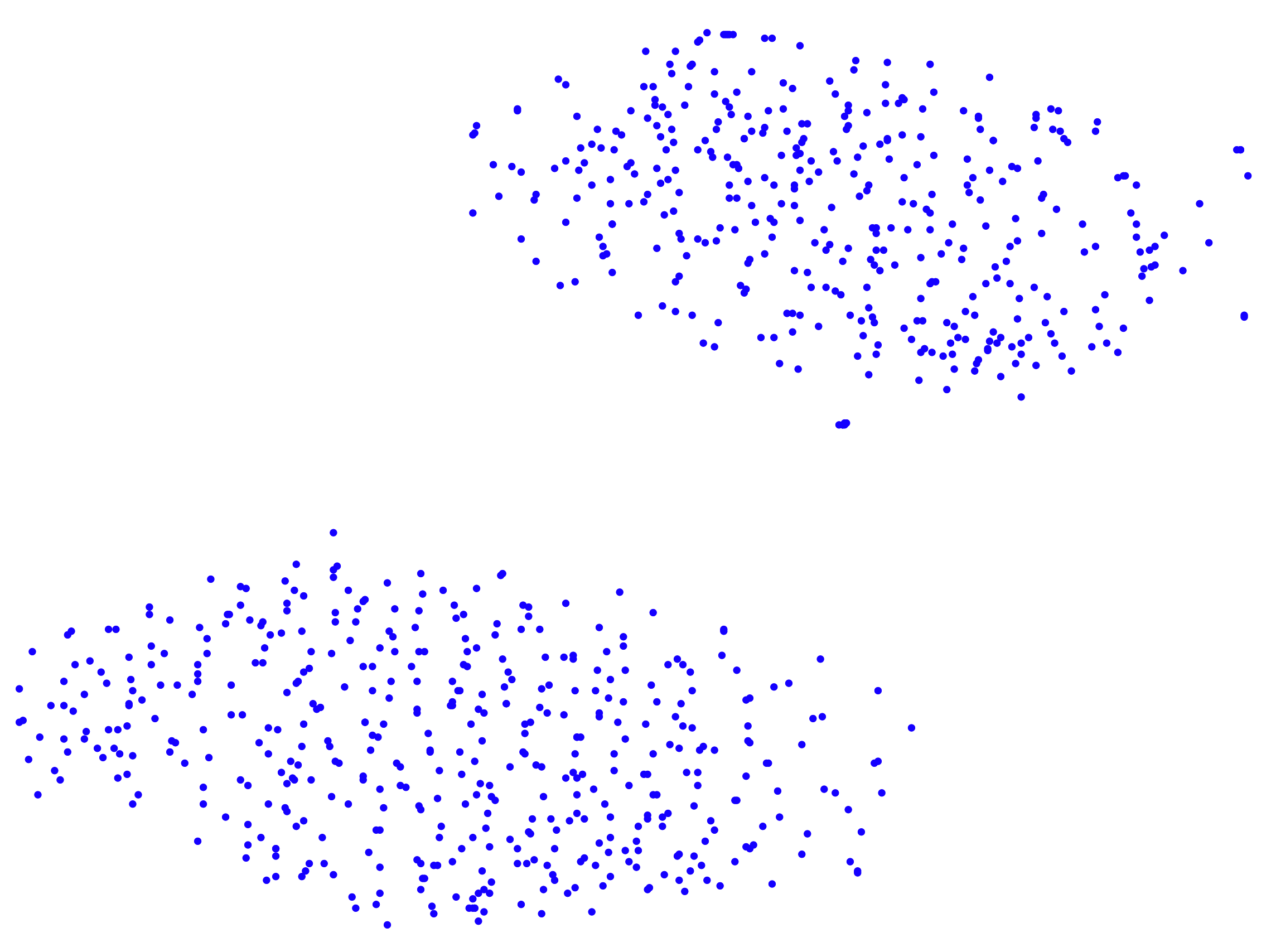

Supplement: Supplementary file 2 — ZIP archive containing VizBin visualization screenshots of the individual bins for the three datasets (37A, 37B, and SRS013705) originally reported in [ 16 ]. [file 40168_2014_66_MOESM2_ESM.zip › 37A_37B_SRS013705/SRS013705/SRS13705.out.004.png]

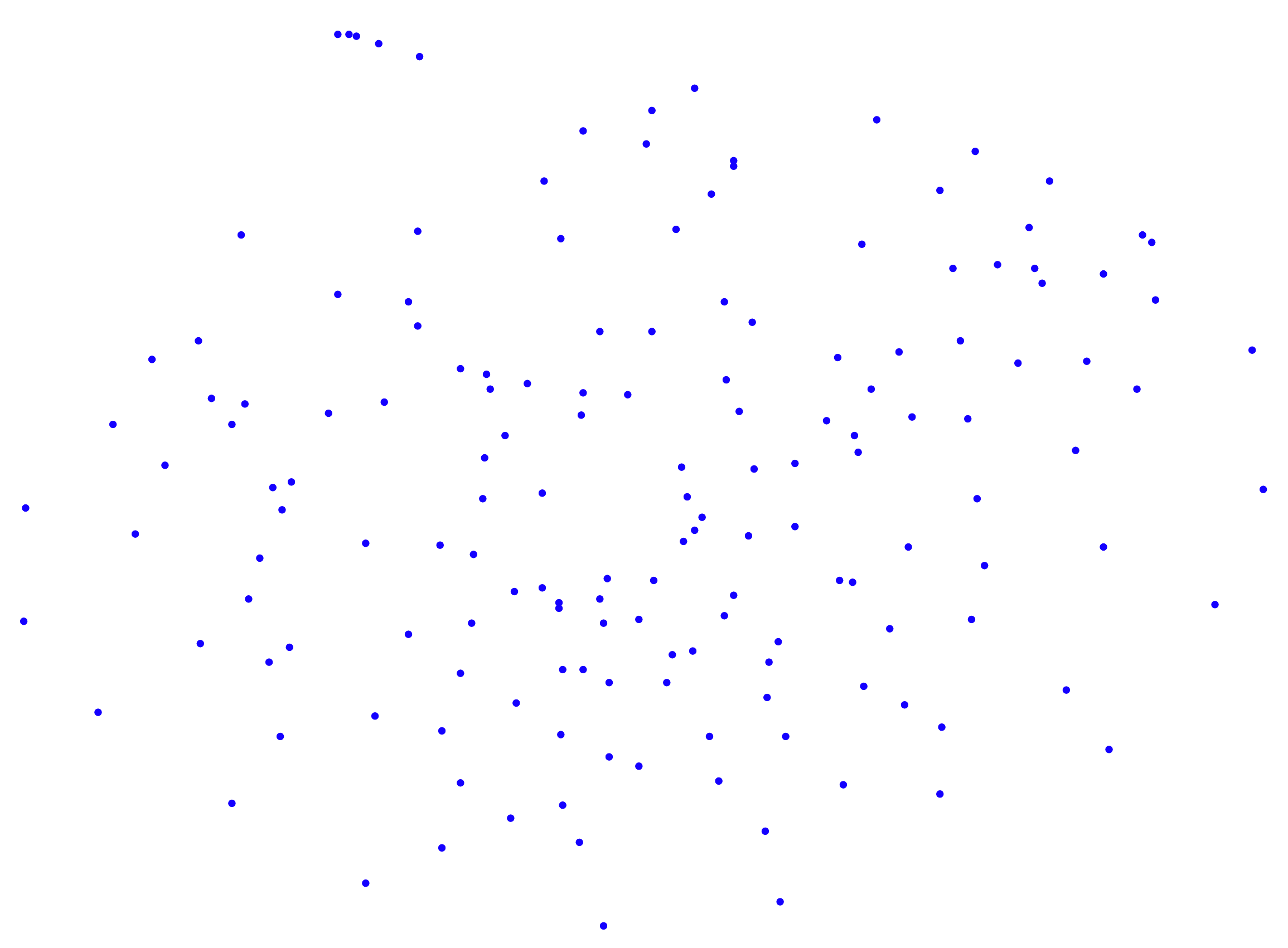

Supplement: Supplementary file 2 — ZIP archive containing VizBin visualization screenshots of the individual bins for the three datasets (37A, 37B, and SRS013705) originally reported in [ 16 ]. [file 40168_2014_66_MOESM2_ESM.zip › 37A_37B_SRS013705/SRS013705/SRS13705.out.005.png]

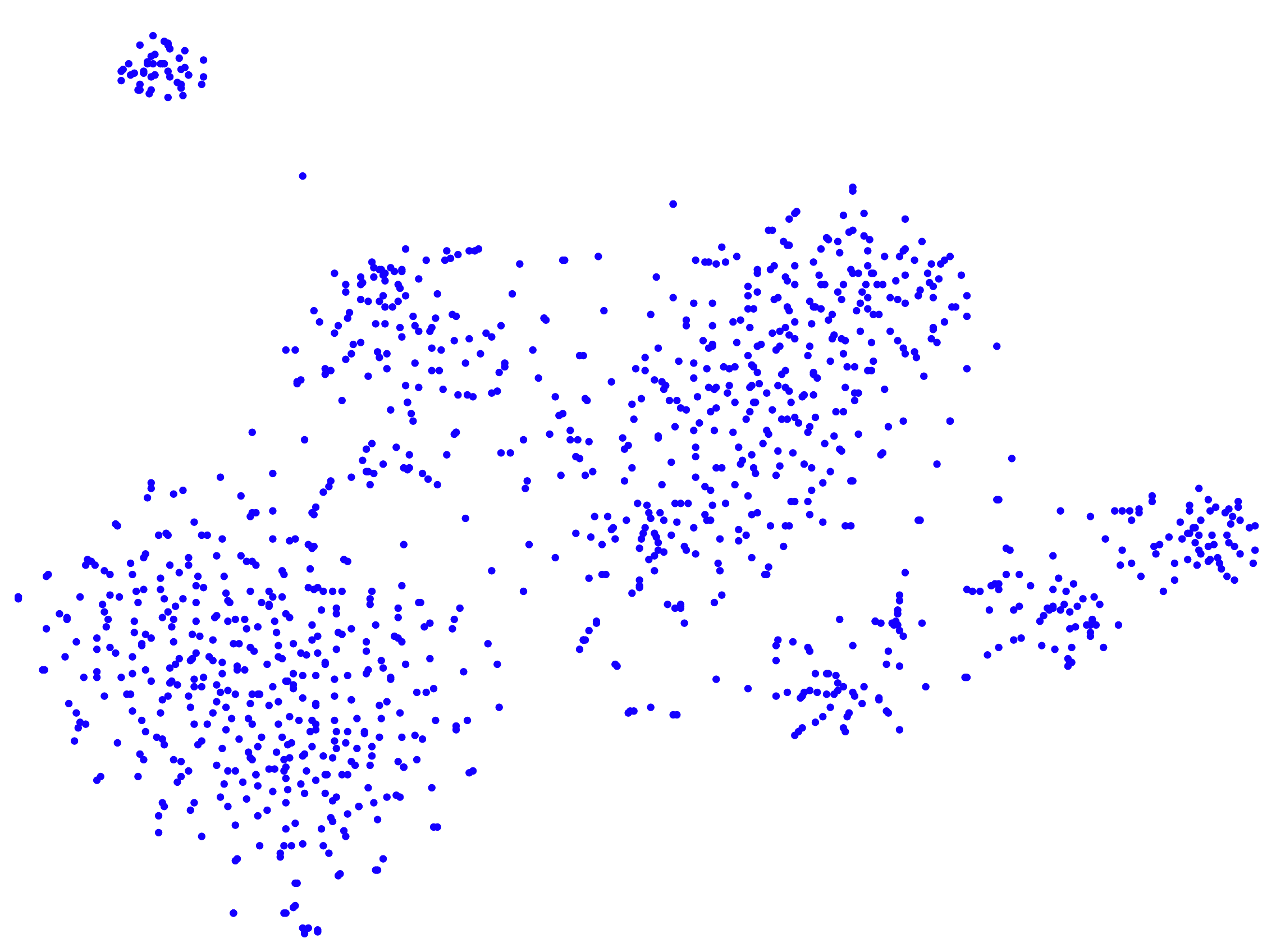

Supplement: Supplementary file 2 — ZIP archive containing VizBin visualization screenshots of the individual bins for the three datasets (37A, 37B, and SRS013705) originally reported in [ 16 ]. [file 40168_2014_66_MOESM2_ESM.zip › 37A_37B_SRS013705/SRS013705/SRS13705.out.006.png]

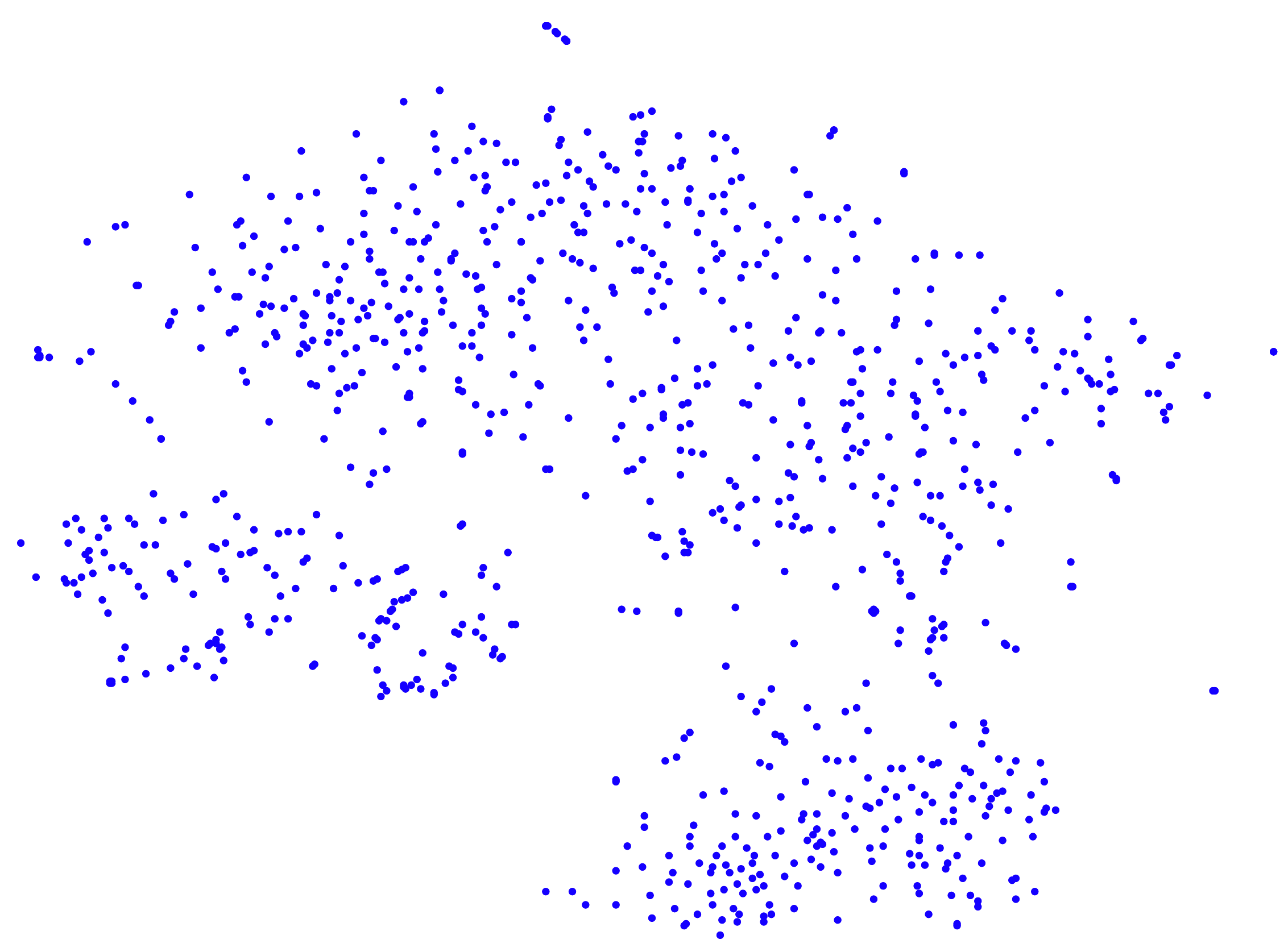

Supplement: Supplementary file 2 — ZIP archive containing VizBin visualization screenshots of the individual bins for the three datasets (37A, 37B, and SRS013705) originally reported in [ 16 ]. [file 40168_2014_66_MOESM2_ESM.zip › 37A_37B_SRS013705/SRS013705/SRS13705.out.007.png]

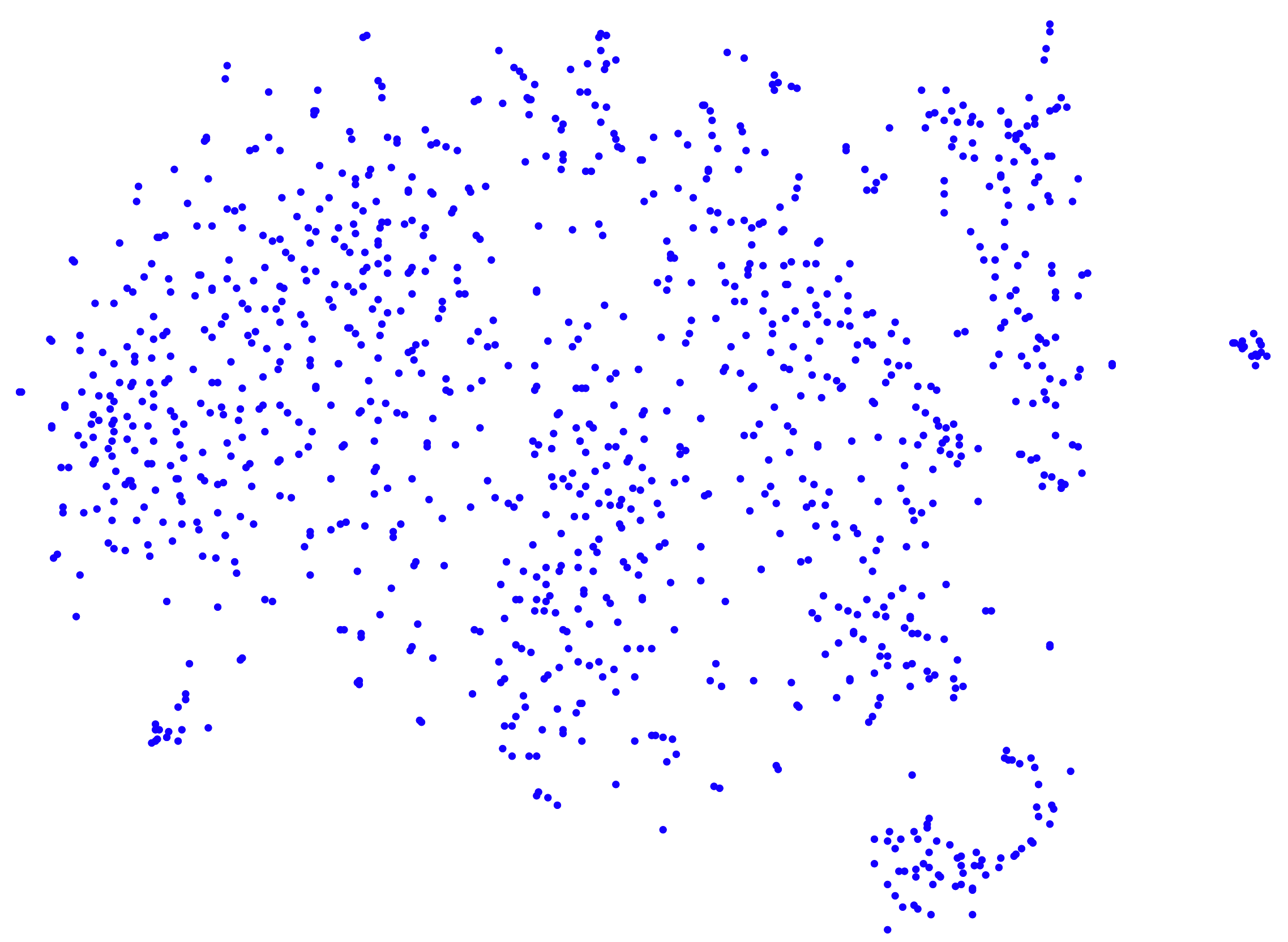

Supplement: Supplementary file 2 — ZIP archive containing VizBin visualization screenshots of the individual bins for the three datasets (37A, 37B, and SRS013705) originally reported in [ 16 ]. [file 40168_2014_66_MOESM2_ESM.zip › 37A_37B_SRS013705/SRS013705/SRS13705.out.008.png]

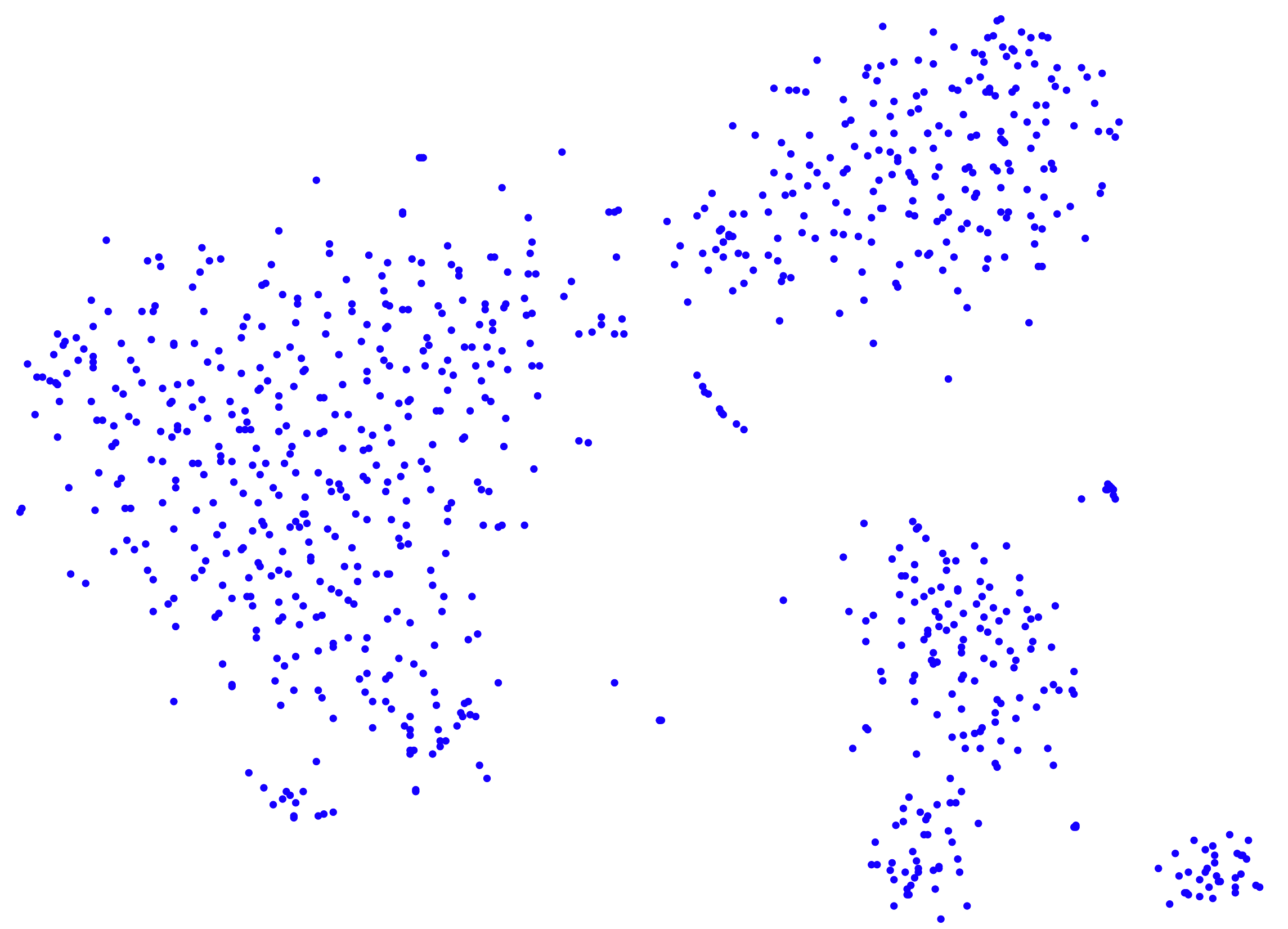

Supplement: Supplementary file 2 — ZIP archive containing VizBin visualization screenshots of the individual bins for the three datasets (37A, 37B, and SRS013705) originally reported in [ 16 ]. [file 40168_2014_66_MOESM2_ESM.zip › 37A_37B_SRS013705/SRS013705/SRS13705.out.009.png]

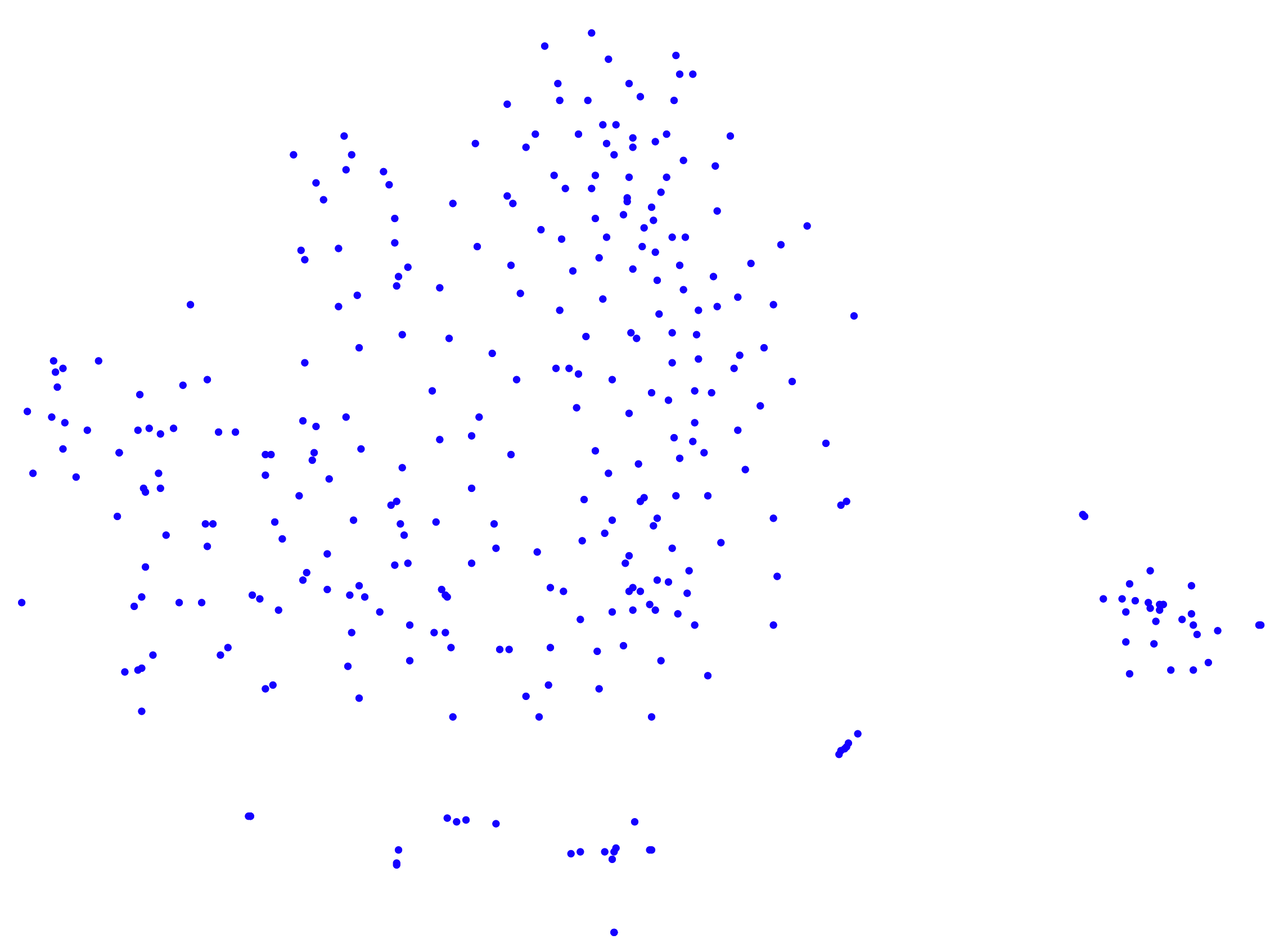

Supplement: Supplementary file 2 — ZIP archive containing VizBin visualization screenshots of the individual bins for the three datasets (37A, 37B, and SRS013705) originally reported in [ 16 ]. [file 40168_2014_66_MOESM2_ESM.zip › 37A_37B_SRS013705/SRS013705/SRS13705.out.010.png]

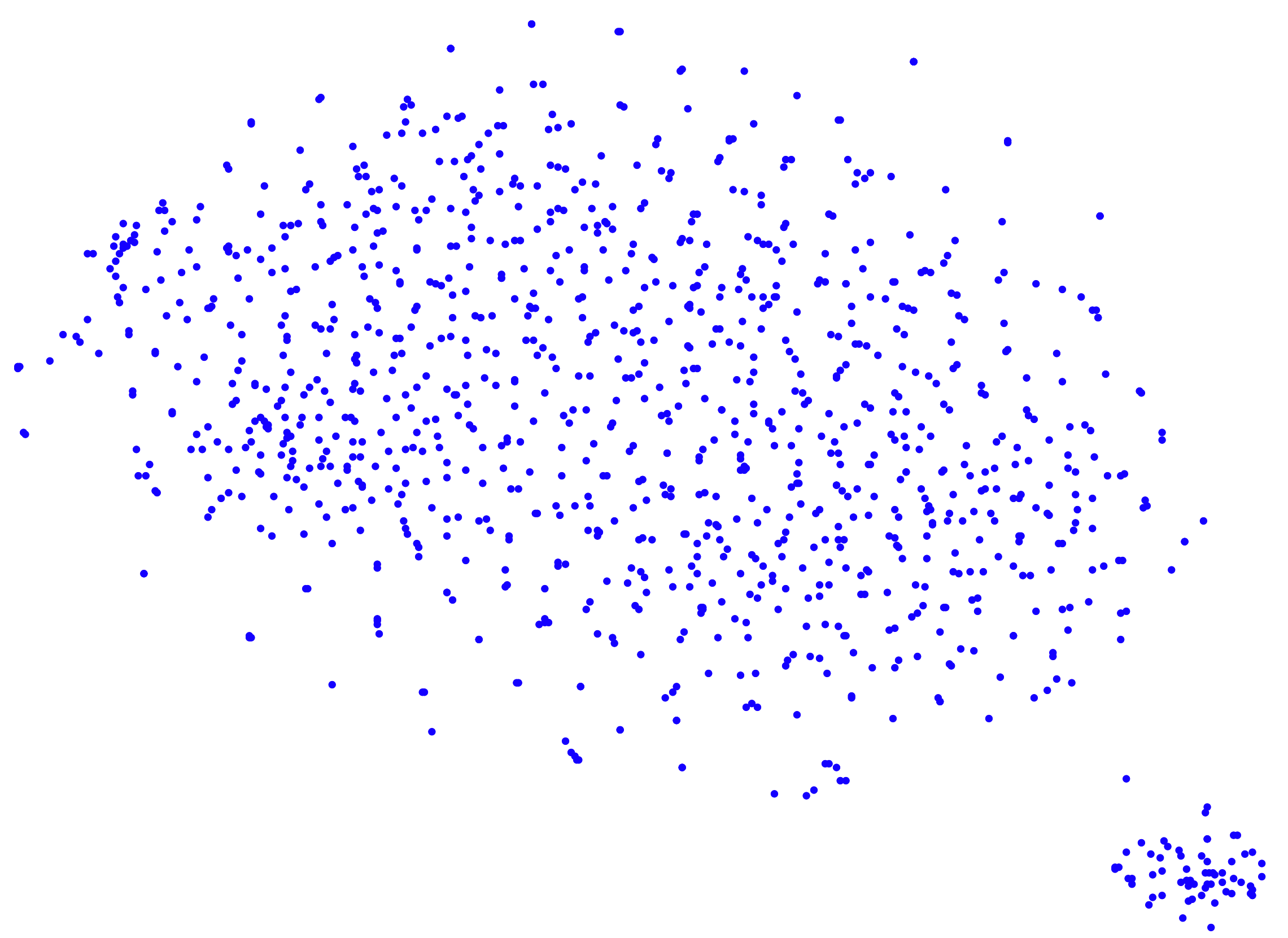

Supplement: Supplementary file 2 — ZIP archive containing VizBin visualization screenshots of the individual bins for the three datasets (37A, 37B, and SRS013705) originally reported in [ 16 ]. [file 40168_2014_66_MOESM2_ESM.zip › 37A_37B_SRS013705/SRS013705/SRS13705.out.011.png]

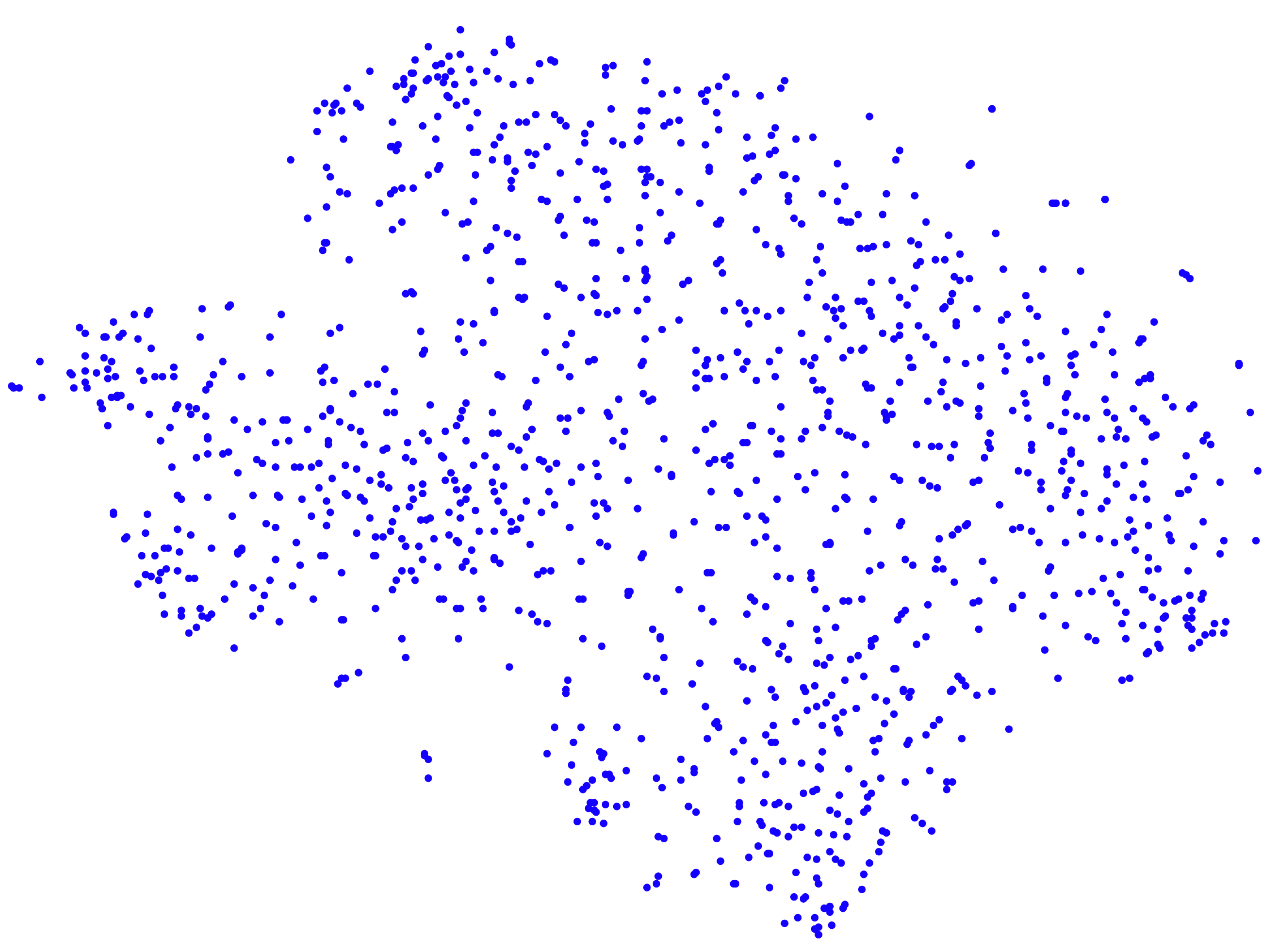

Supplement: Supplementary file 2 — ZIP archive containing VizBin visualization screenshots of the individual bins for the three datasets (37A, 37B, and SRS013705) originally reported in [ 16 ]. [file 40168_2014_66_MOESM2_ESM.zip › 37A_37B_SRS013705/SRS013705/SRS13705.out.012.png]

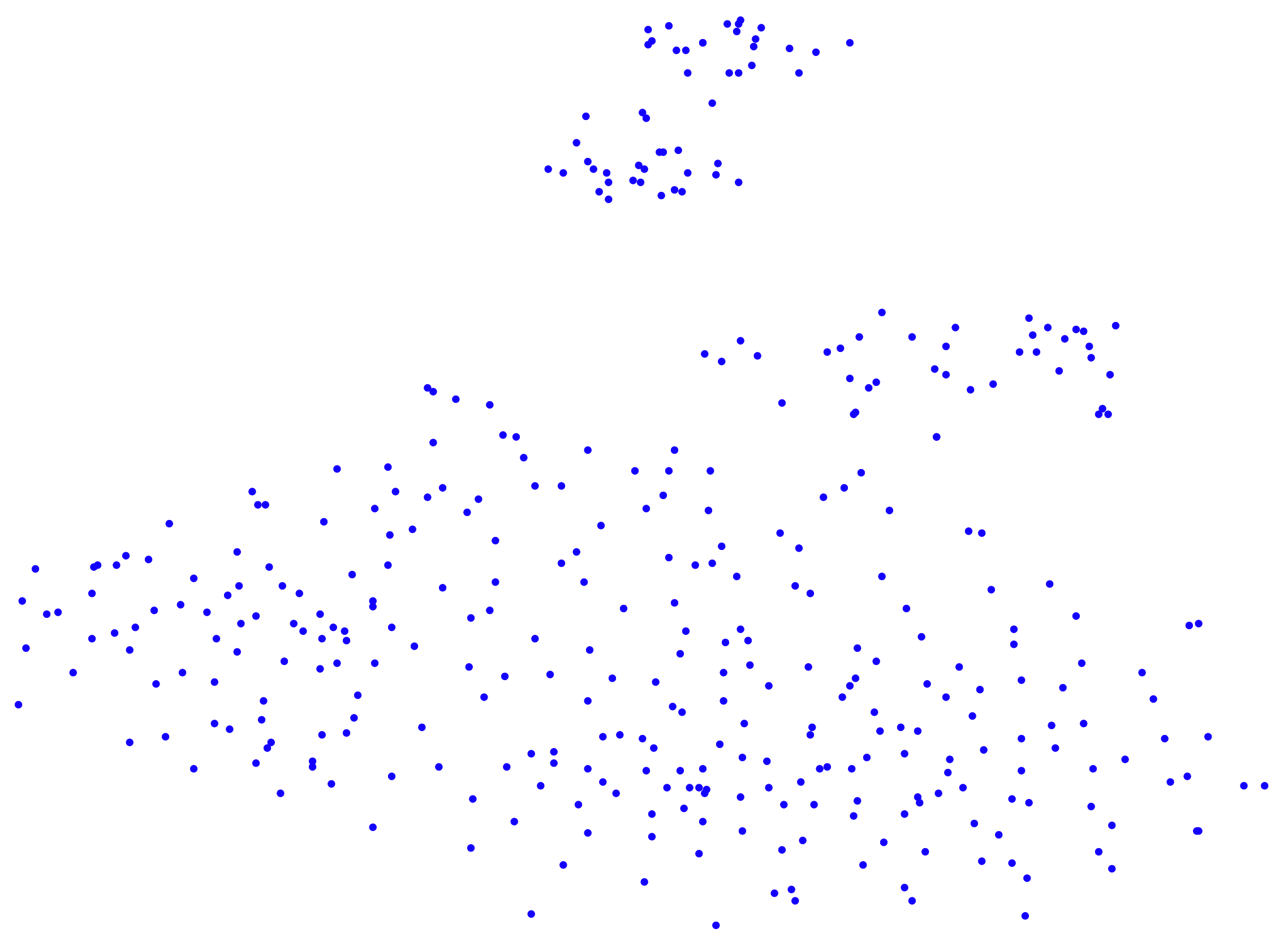

Supplement: Supplementary file 2 — ZIP archive containing VizBin visualization screenshots of the individual bins for the three datasets (37A, 37B, and SRS013705) originally reported in [ 16 ]. [file 40168_2014_66_MOESM2_ESM.zip › 37A_37B_SRS013705/SRS013705/SRS13705.out.013.png]

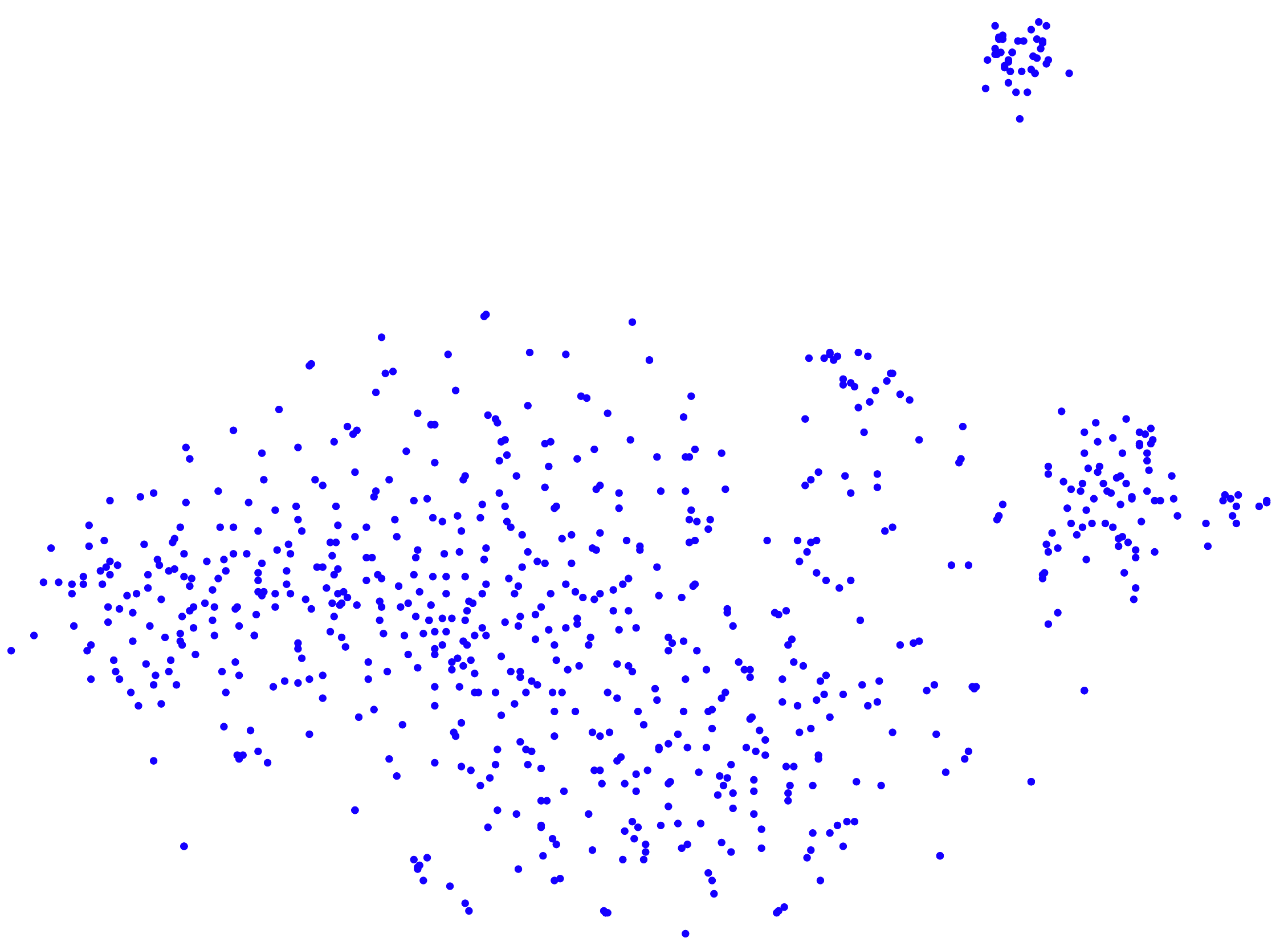

Supplement: Supplementary file 2 — ZIP archive containing VizBin visualization screenshots of the individual bins for the three datasets (37A, 37B, and SRS013705) originally reported in [ 16 ]. [file 40168_2014_66_MOESM2_ESM.zip › 37A_37B_SRS013705/SRS013705/SRS13705.out.014.png]

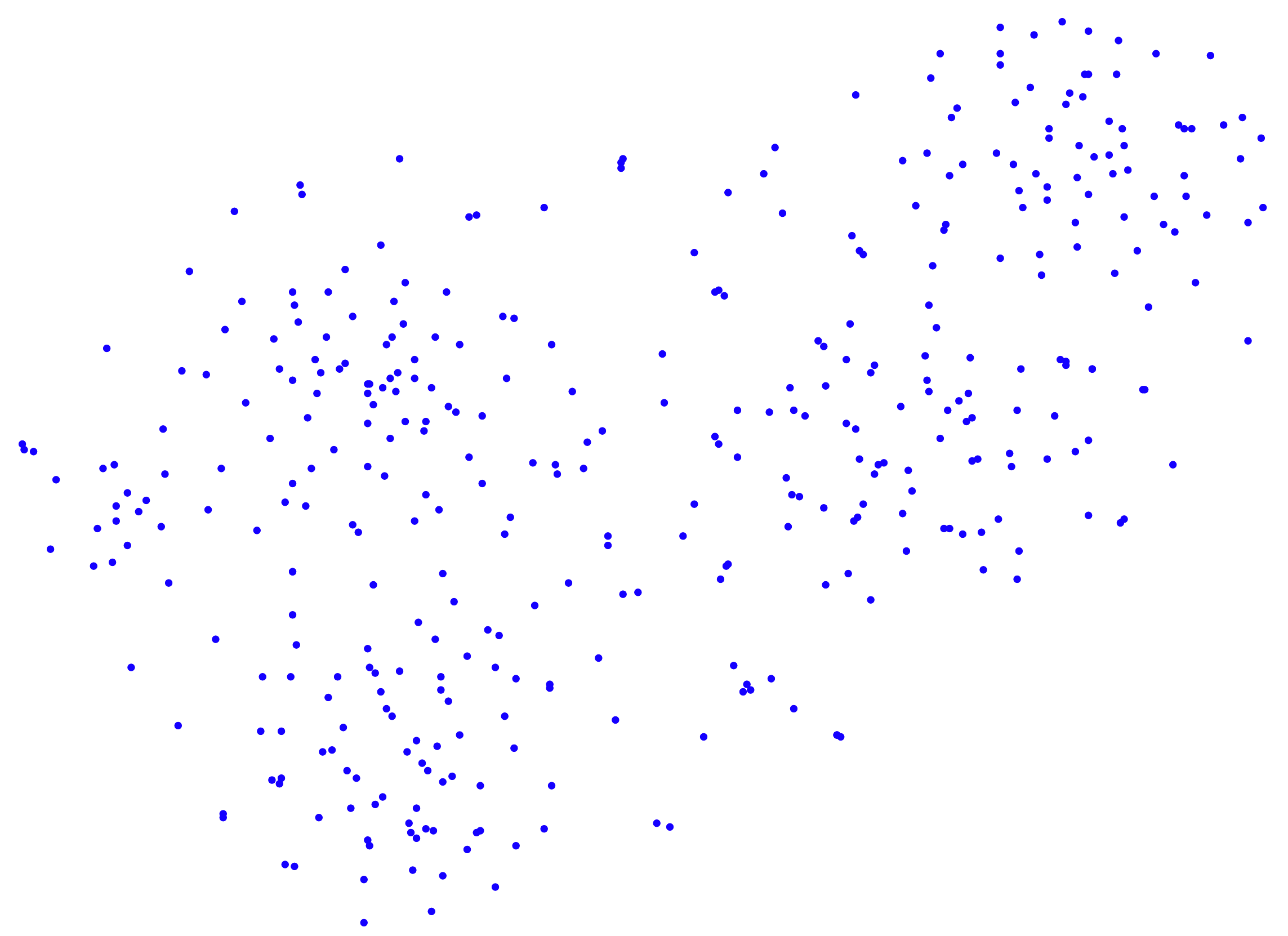

Supplement: Supplementary file 2 — ZIP archive containing VizBin visualization screenshots of the individual bins for the three datasets (37A, 37B, and SRS013705) originally reported in [ 16 ]. [file 40168_2014_66_MOESM2_ESM.zip › 37A_37B_SRS013705/SRS013705/SRS13705.out.015.png]

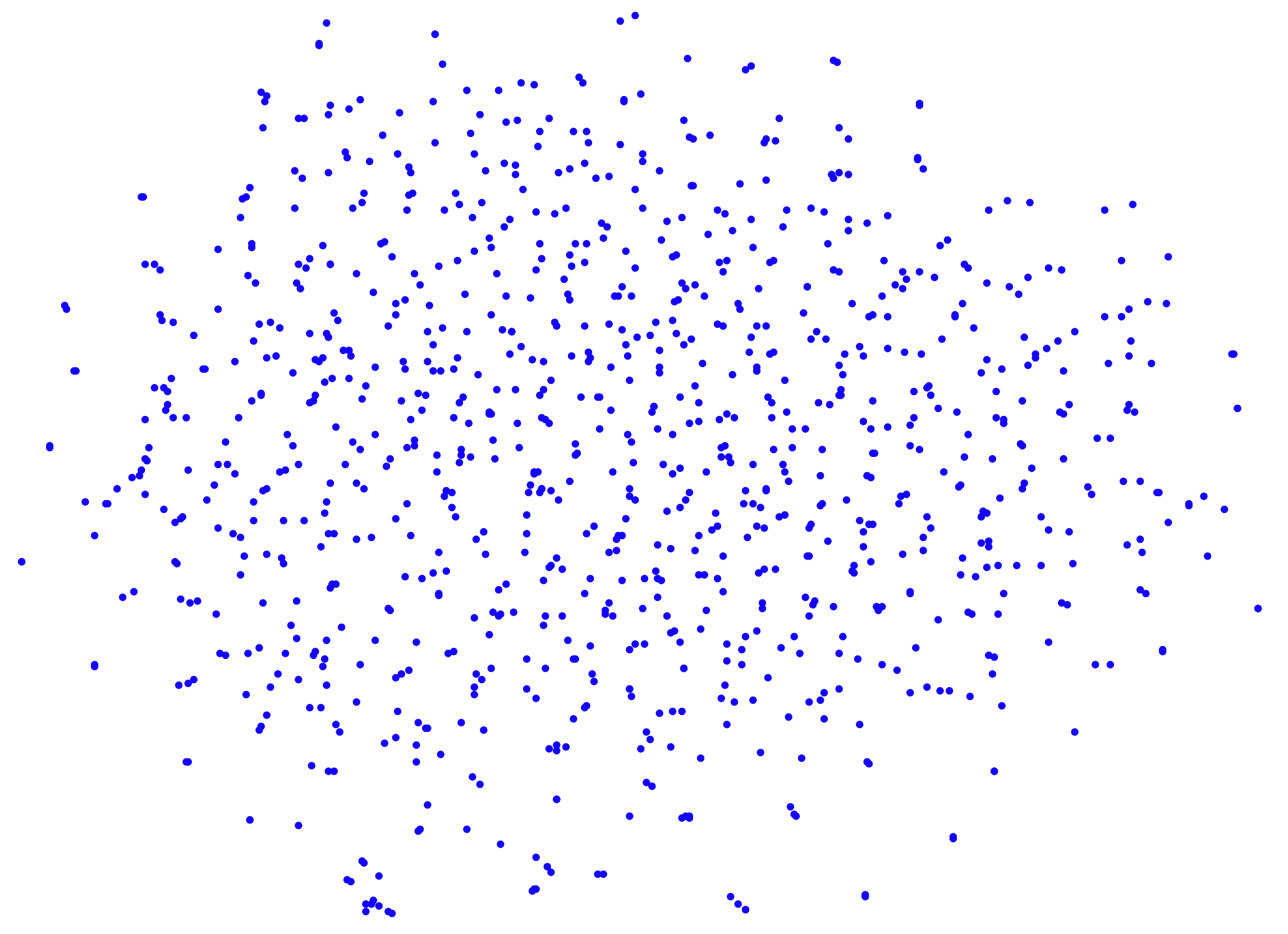

Supplement: Supplementary file 2 — ZIP archive containing VizBin visualization screenshots of the individual bins for the three datasets (37A, 37B, and SRS013705) originally reported in [ 16 ]. [file 40168_2014_66_MOESM2_ESM.zip › 37A_37B_SRS013705/SRS013705/SRS13705.out.016.png]

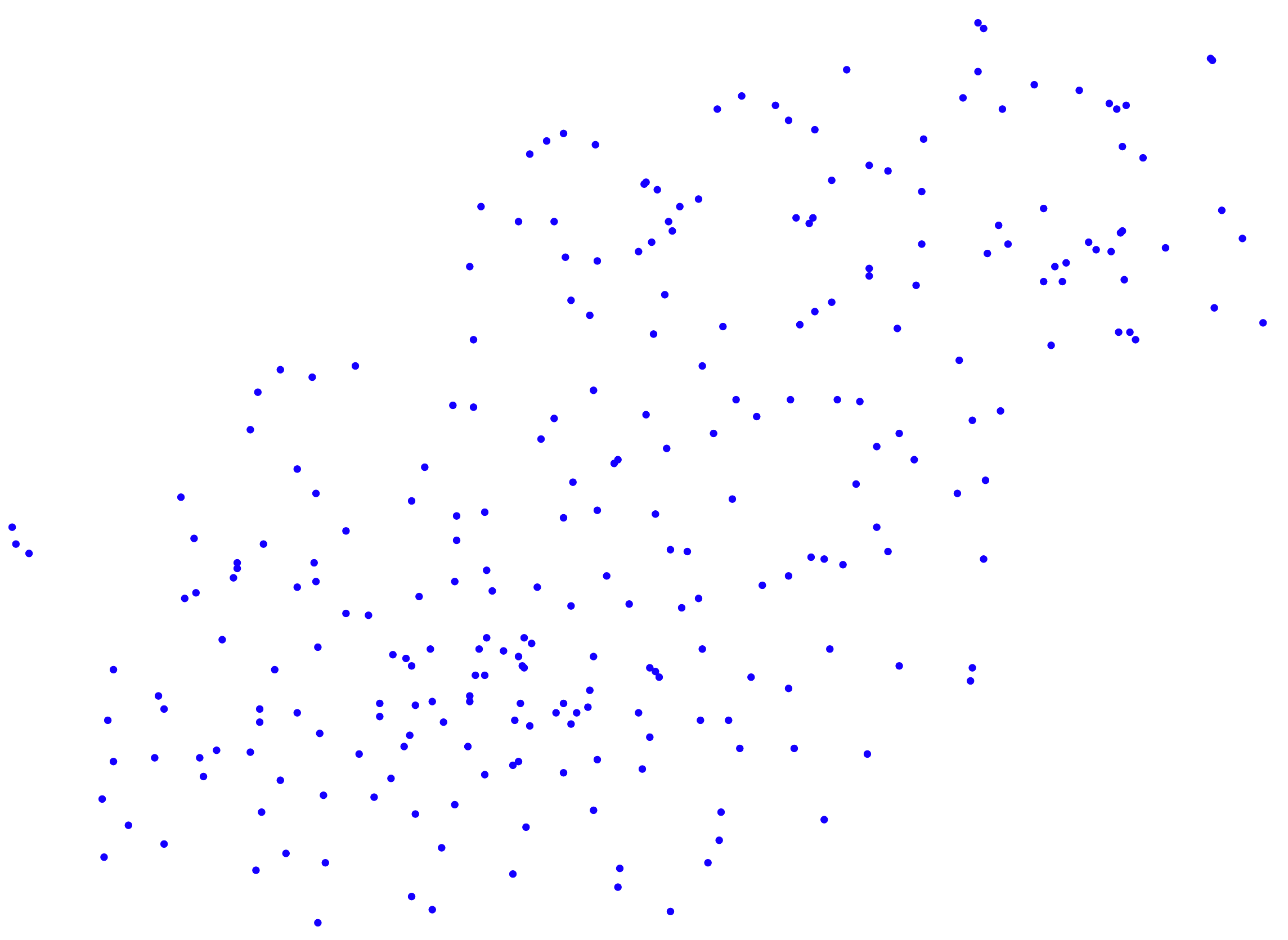

Supplement: Supplementary file 2 — ZIP archive containing VizBin visualization screenshots of the individual bins for the three datasets (37A, 37B, and SRS013705) originally reported in [ 16 ]. [file 40168_2014_66_MOESM2_ESM.zip › 37A_37B_SRS013705/SRS013705/SRS13705.out.017.png]

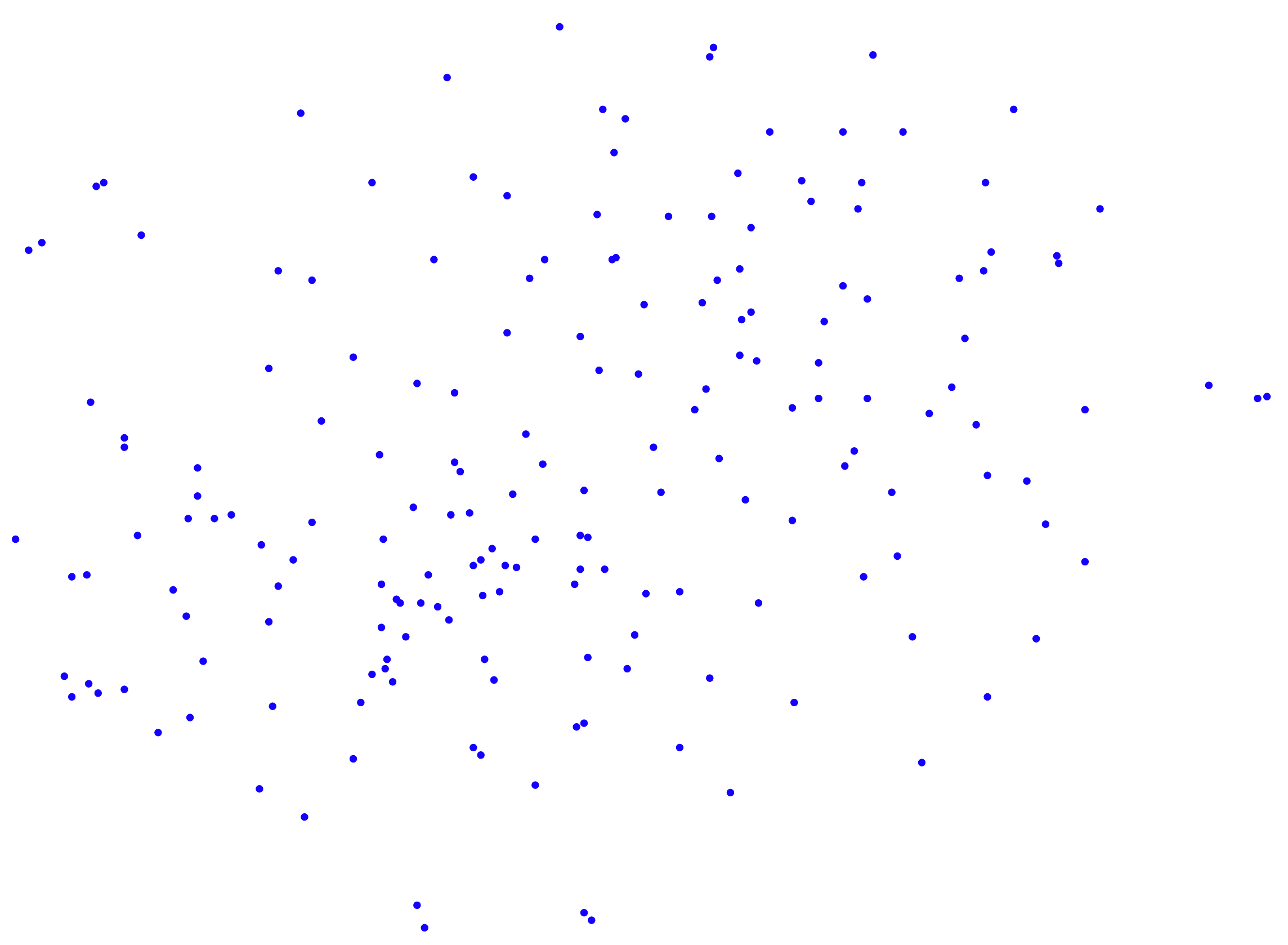

Supplement: Supplementary file 2 — ZIP archive containing VizBin visualization screenshots of the individual bins for the three datasets (37A, 37B, and SRS013705) originally reported in [ 16 ]. [file 40168_2014_66_MOESM2_ESM.zip › 37A_37B_SRS013705/SRS013705/SRS13705.out.018.png]

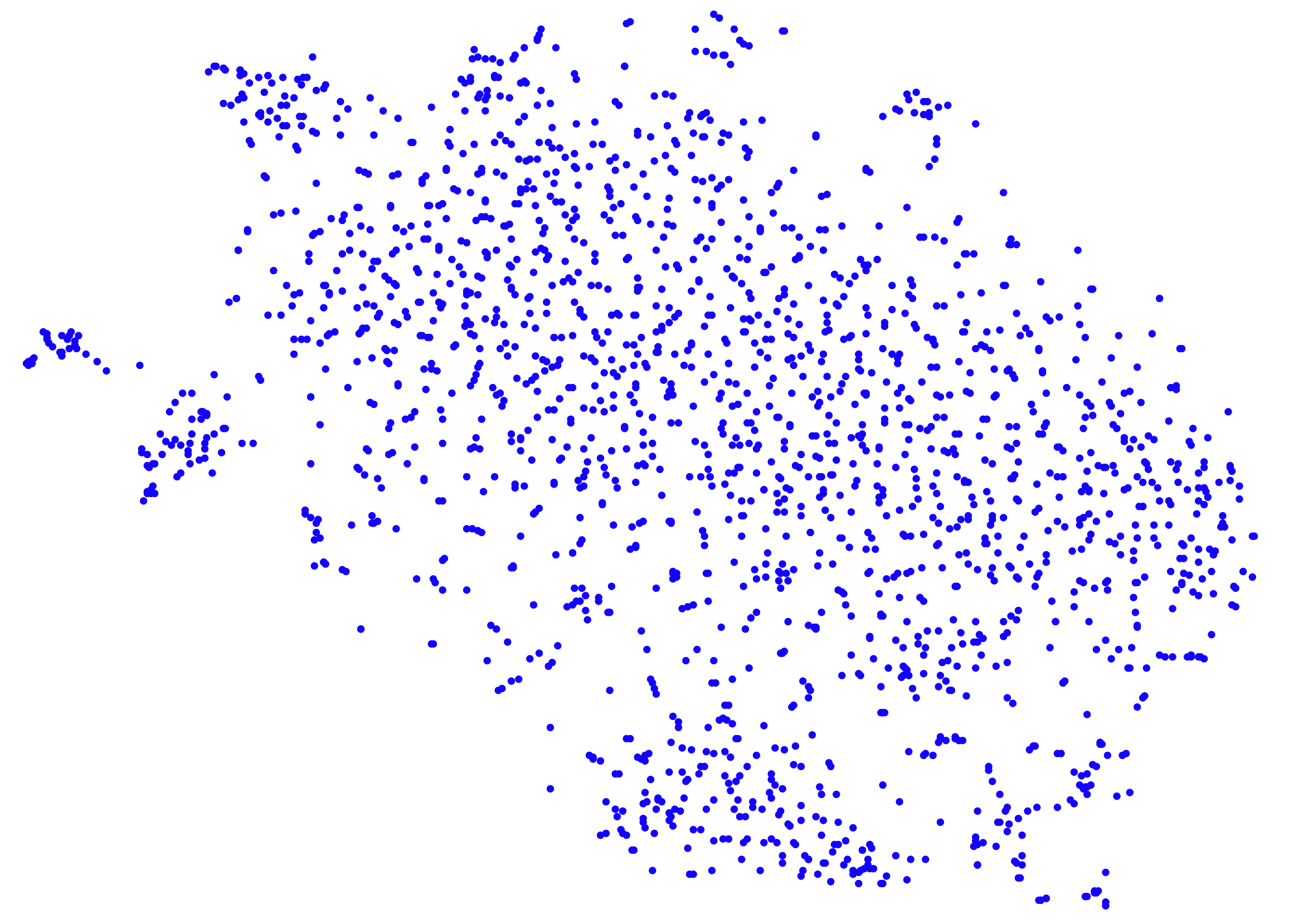

Supplement: Supplementary file 2 — ZIP archive containing VizBin visualization screenshots of the individual bins for the three datasets (37A, 37B, and SRS013705) originally reported in [ 16 ]. [file 40168_2014_66_MOESM2_ESM.zip › 37A_37B_SRS013705/SRS013705/SRS13705.out.019.png]

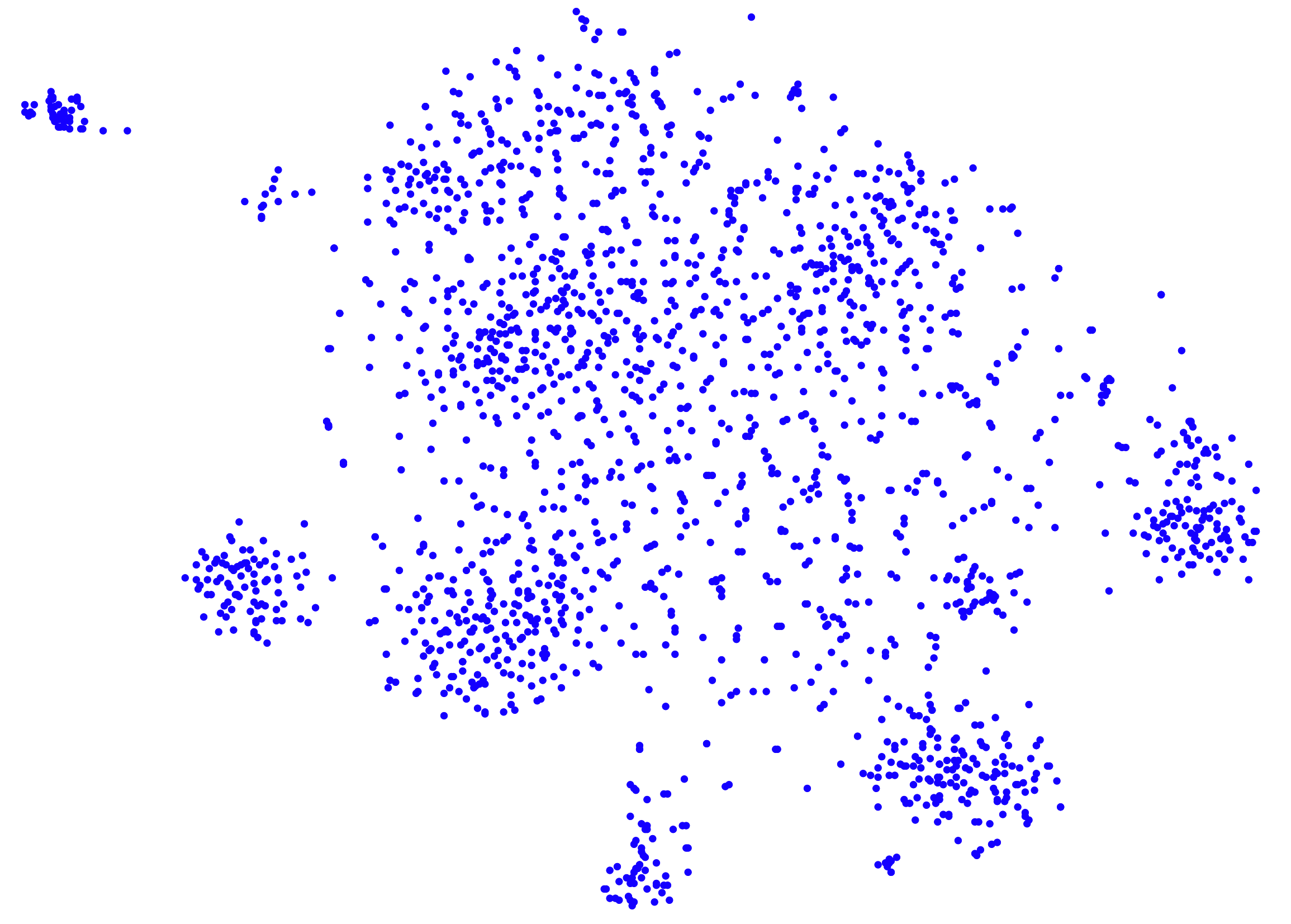

Supplement: Supplementary file 2 — ZIP archive containing VizBin visualization screenshots of the individual bins for the three datasets (37A, 37B, and SRS013705) originally reported in [ 16 ]. [file 40168_2014_66_MOESM2_ESM.zip › 37A_37B_SRS013705/SRS013705/SRS13705.out.020.png]

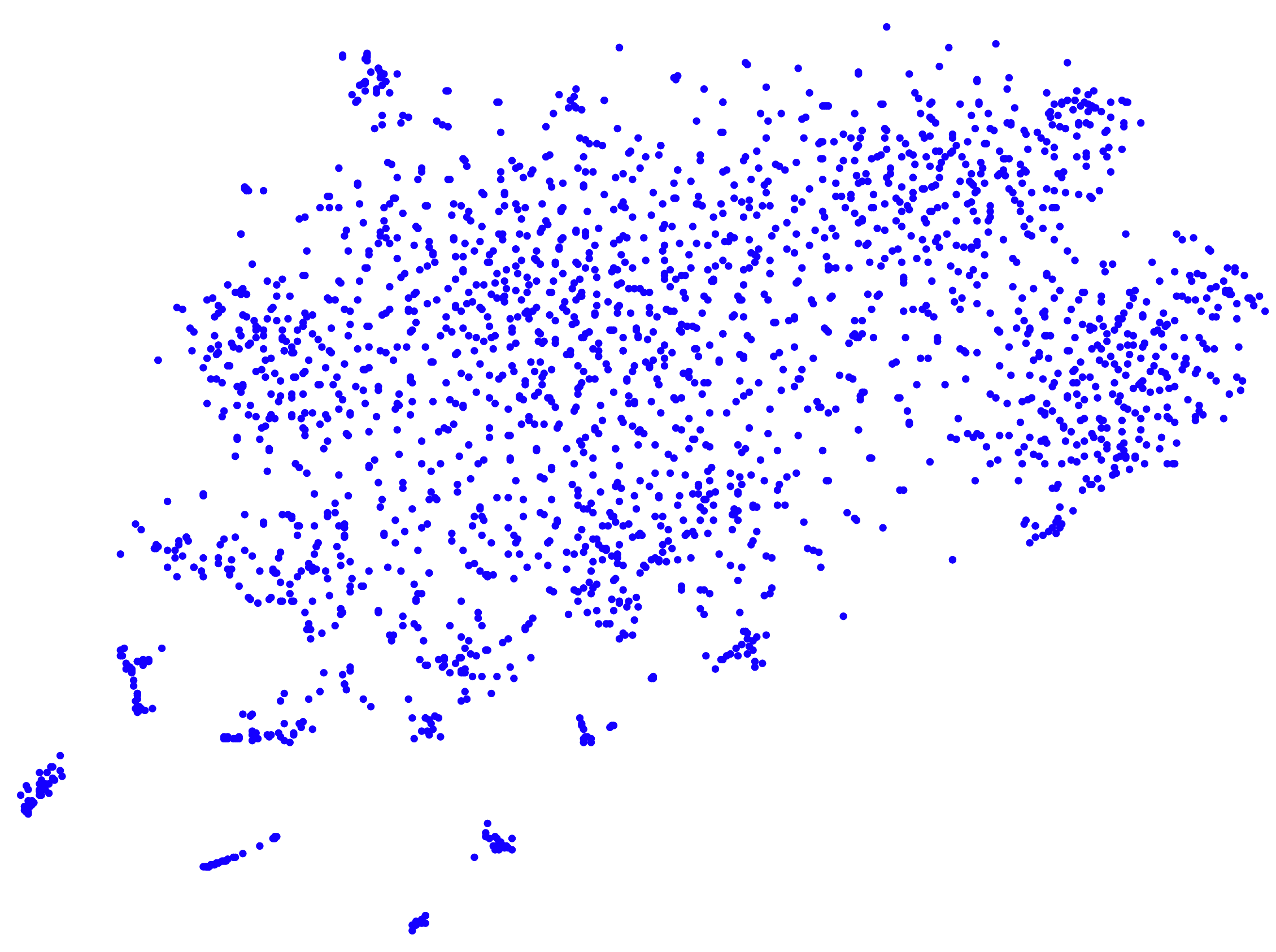

Supplement: Supplementary file 2 — ZIP archive containing VizBin visualization screenshots of the individual bins for the three datasets (37A, 37B, and SRS013705) originally reported in [ 16 ]. [file 40168_2014_66_MOESM2_ESM.zip › 37A_37B_SRS013705/SRS013705/SRS13705.out.021.png]

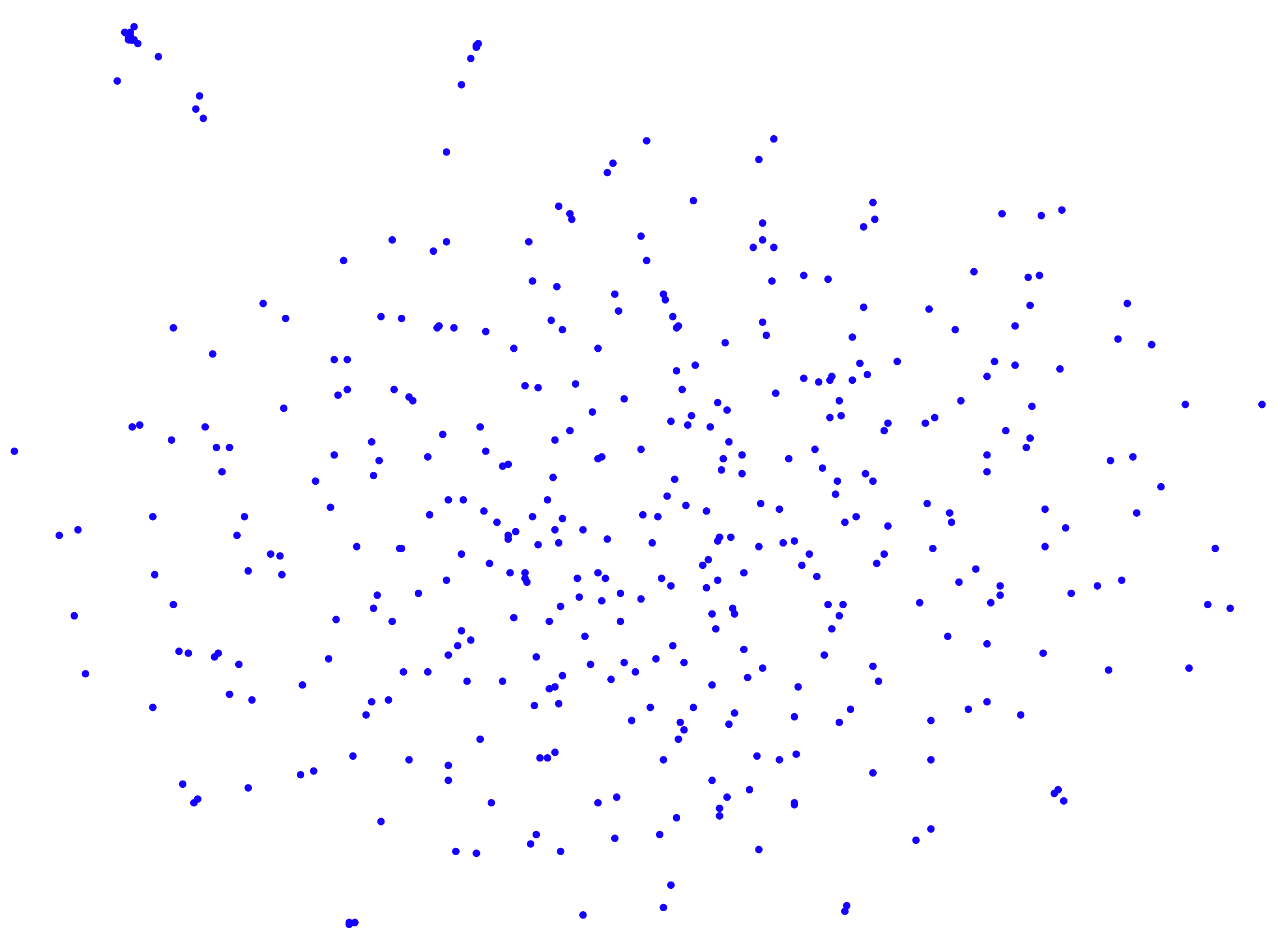

Supplement: Supplementary file 2 — ZIP archive containing VizBin visualization screenshots of the individual bins for the three datasets (37A, 37B, and SRS013705) originally reported in [ 16 ]. [file 40168_2014_66_MOESM2_ESM.zip › 37A_37B_SRS013705/SRS013705/SRS13705.out.022.png]

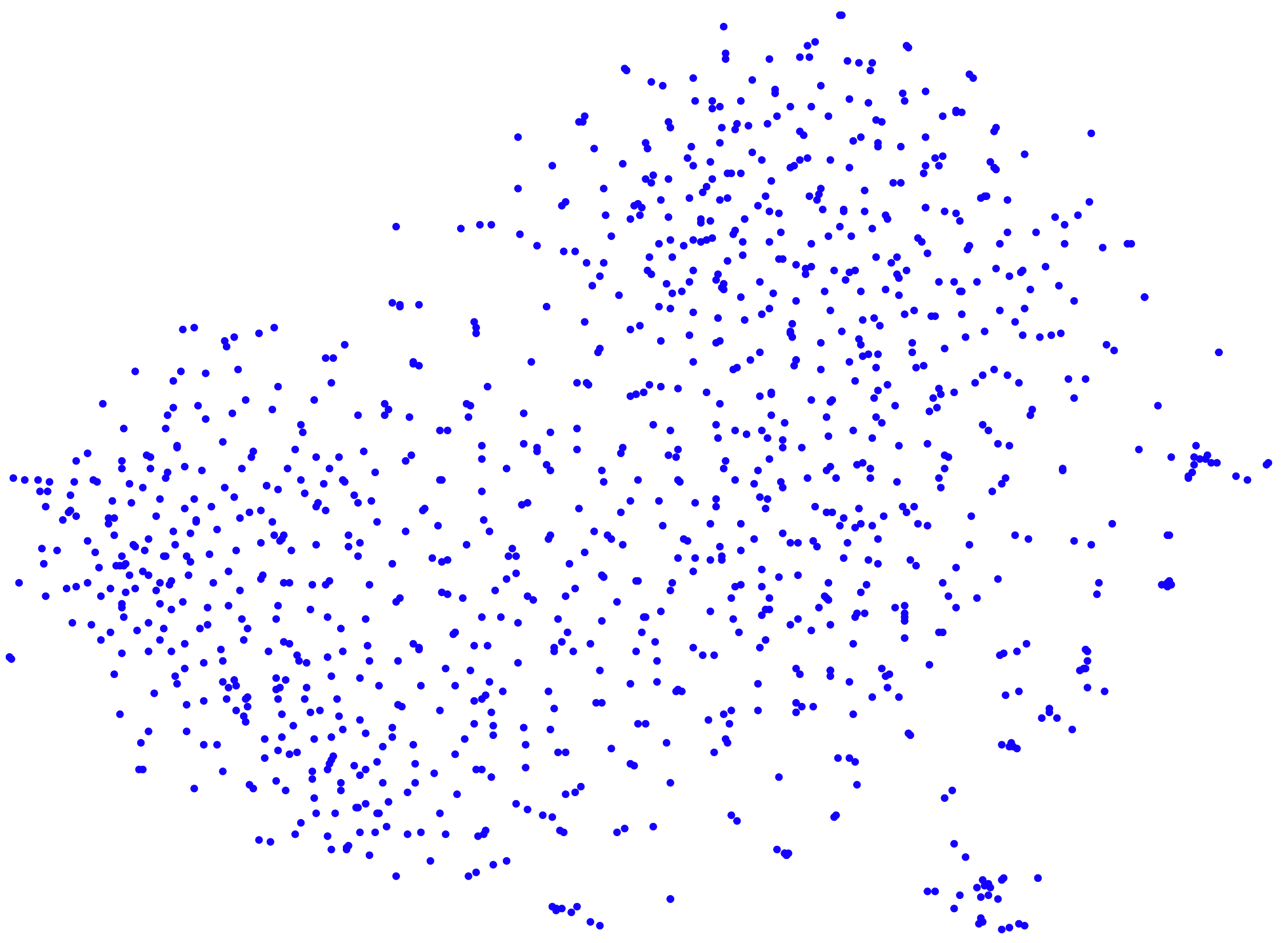

Supplement: Supplementary file 2 — ZIP archive containing VizBin visualization screenshots of the individual bins for the three datasets (37A, 37B, and SRS013705) originally reported in [ 16 ]. [file 40168_2014_66_MOESM2_ESM.zip › 37A_37B_SRS013705/SRS013705/SRS13705.out.023.png]

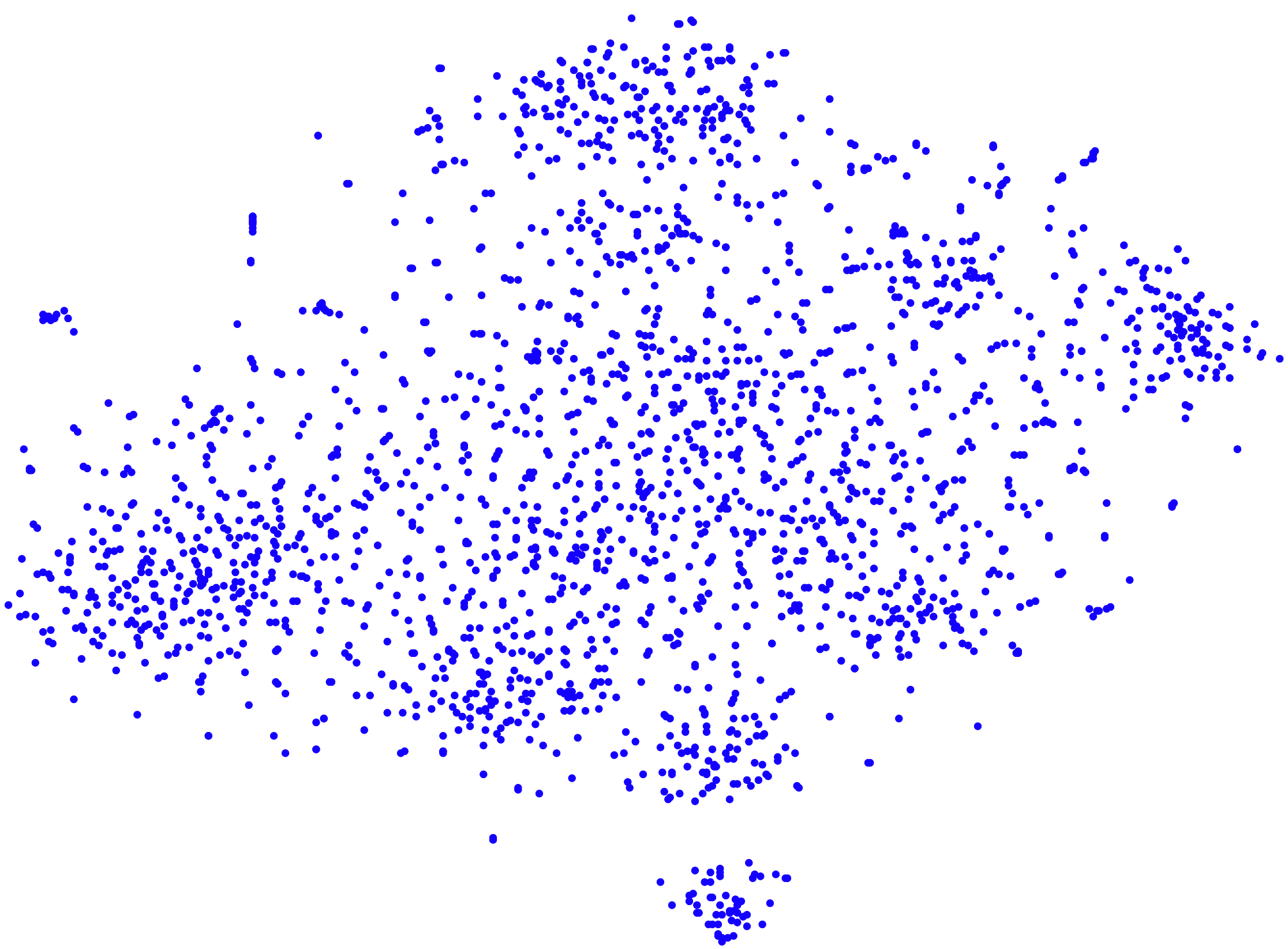

Supplement: Supplementary file 2 — ZIP archive containing VizBin visualization screenshots of the individual bins for the three datasets (37A, 37B, and SRS013705) originally reported in [ 16 ]. [file 40168_2014_66_MOESM2_ESM.zip › 37A_37B_SRS013705/SRS013705/SRS13705.out.024.png]

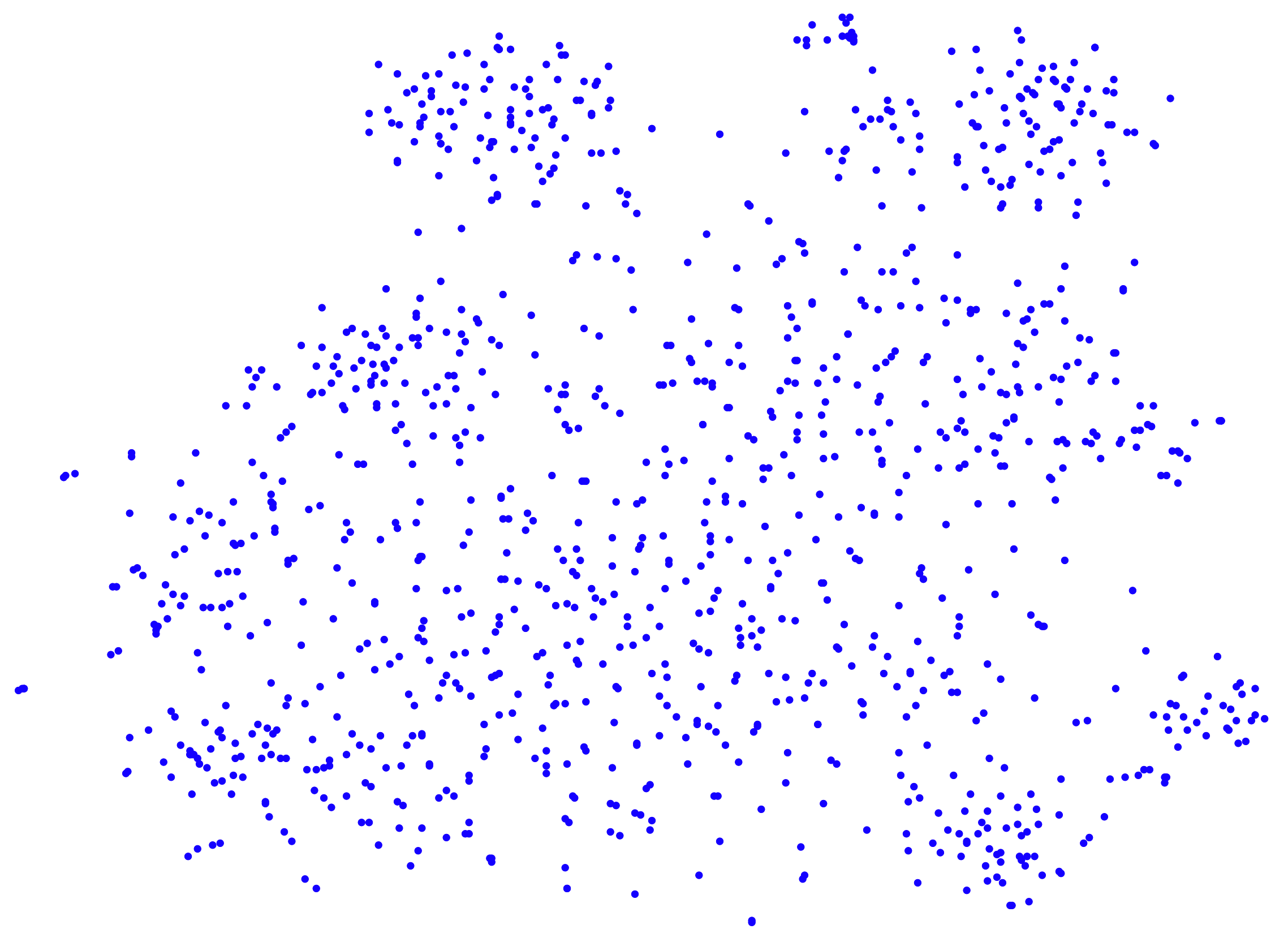

Supplement: Supplementary file 2 — ZIP archive containing VizBin visualization screenshots of the individual bins for the three datasets (37A, 37B, and SRS013705) originally reported in [ 16 ]. [file 40168_2014_66_MOESM2_ESM.zip › 37A_37B_SRS013705/SRS013705/SRS13705.out.025.png]

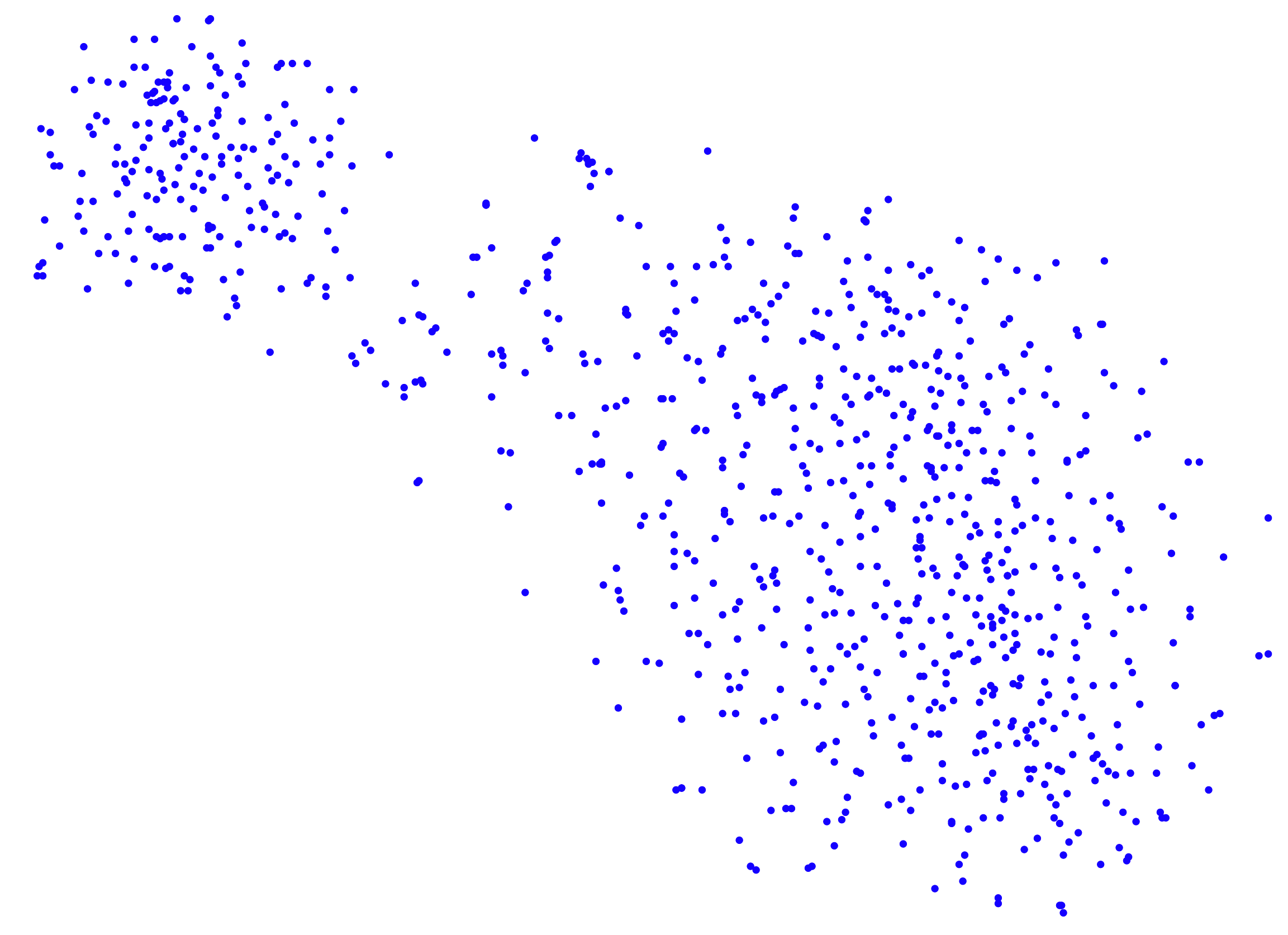

Supplement: Supplementary file 2 — ZIP archive containing VizBin visualization screenshots of the individual bins for the three datasets (37A, 37B, and SRS013705) originally reported in [ 16 ]. [file 40168_2014_66_MOESM2_ESM.zip › 37A_37B_SRS013705/SRS013705/SRS13705.out.026.png]

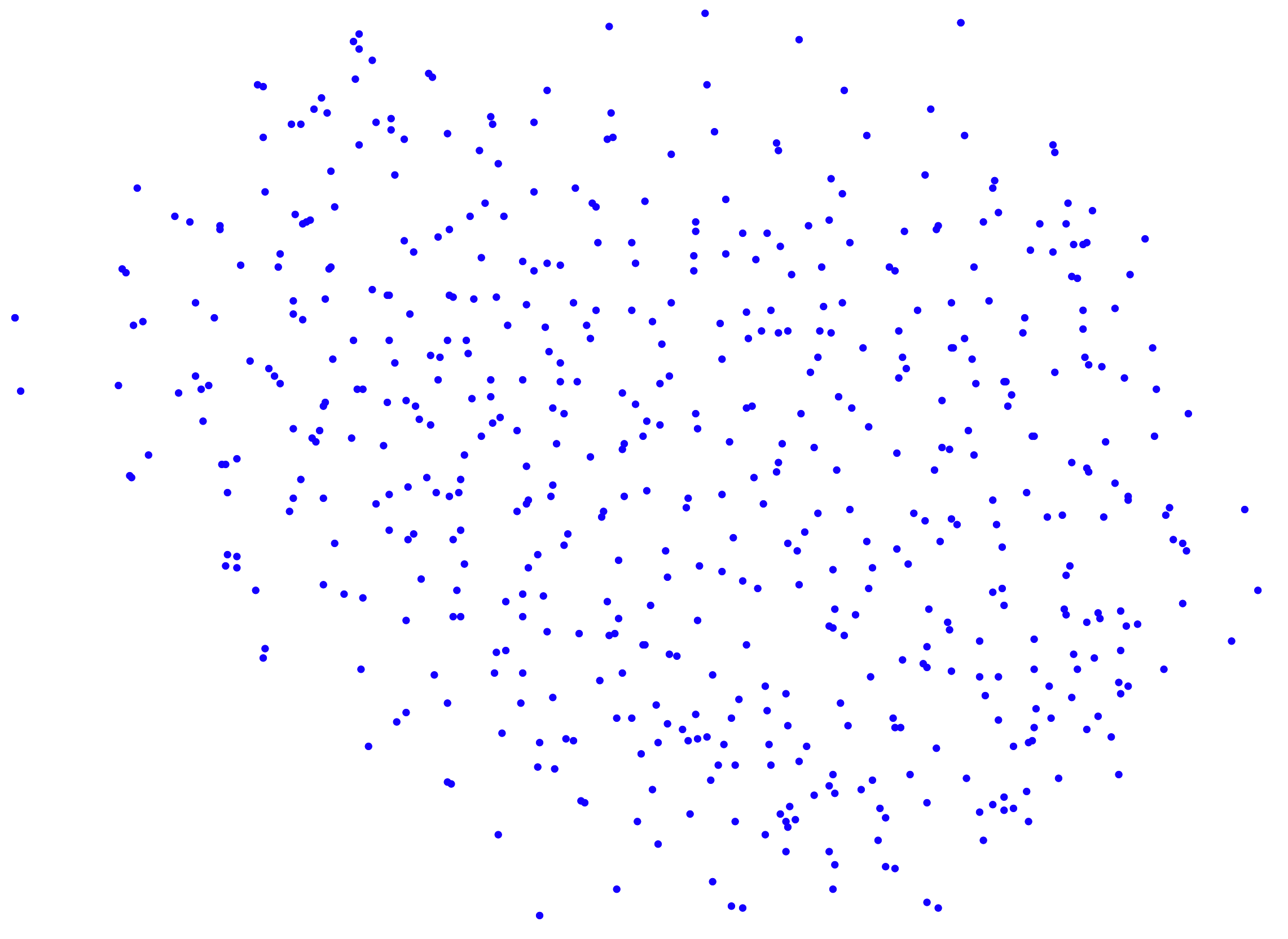

Supplement: Supplementary file 2 — ZIP archive containing VizBin visualization screenshots of the individual bins for the three datasets (37A, 37B, and SRS013705) originally reported in [ 16 ]. [file 40168_2014_66_MOESM2_ESM.zip › 37A_37B_SRS013705/SRS013705/SRS13705.out.027.png]

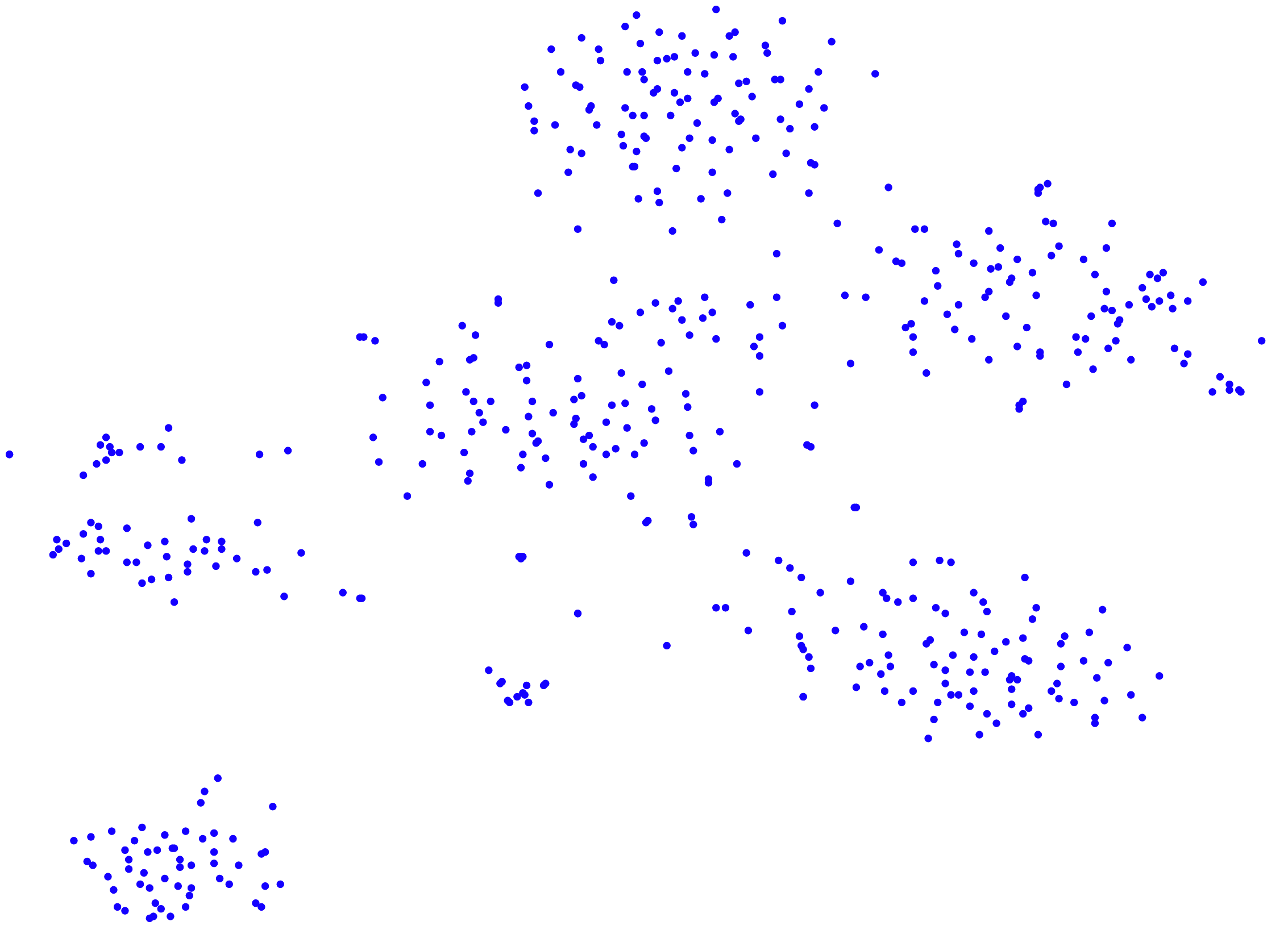

Supplement: Supplementary file 2 — ZIP archive containing VizBin visualization screenshots of the individual bins for the three datasets (37A, 37B, and SRS013705) originally reported in [ 16 ]. [file 40168_2014_66_MOESM2_ESM.zip › 37A_37B_SRS013705/SRS013705/SRS13705.out.028.png]

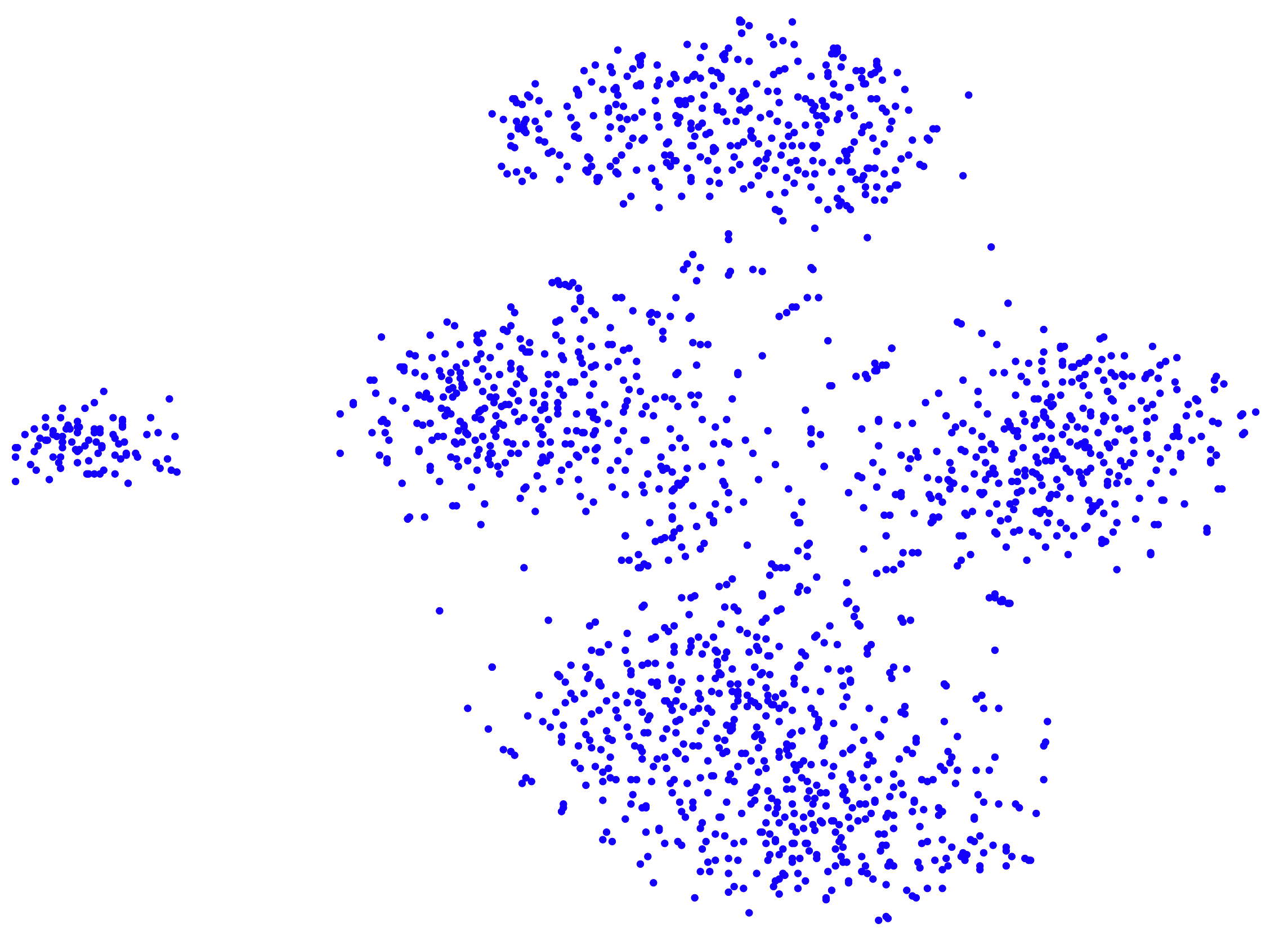

Supplement: Supplementary file 2 — ZIP archive containing VizBin visualization screenshots of the individual bins for the three datasets (37A, 37B, and SRS013705) originally reported in [ 16 ]. [file 40168_2014_66_MOESM2_ESM.zip › 37A_37B_SRS013705/SRS013705/SRS13705.out.029.png]

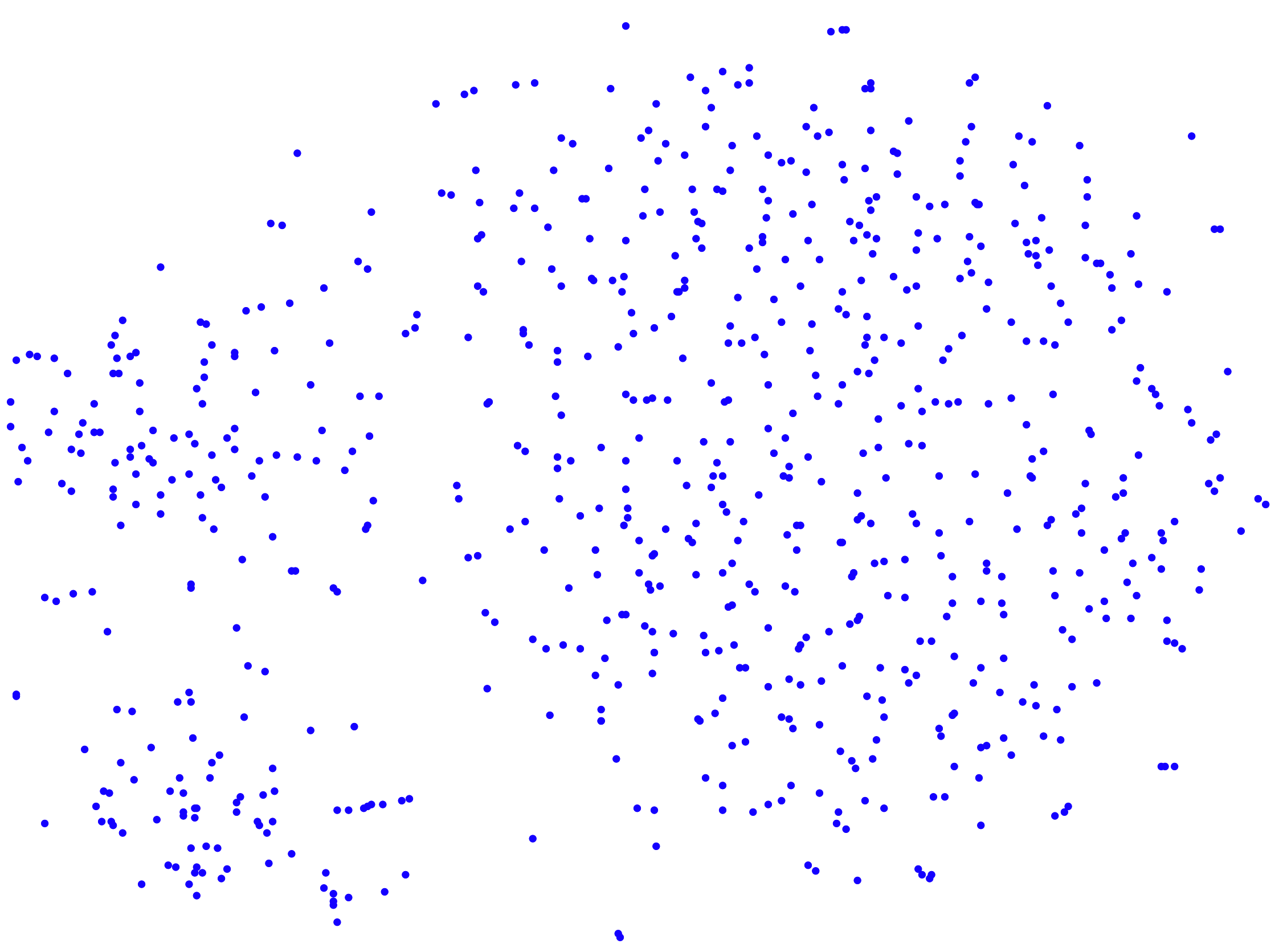

Supplement: Supplementary file 2 — ZIP archive containing VizBin visualization screenshots of the individual bins for the three datasets (37A, 37B, and SRS013705) originally reported in [ 16 ]. [file 40168_2014_66_MOESM2_ESM.zip › 37A_37B_SRS013705/SRS013705/SRS13705.out.030.png]

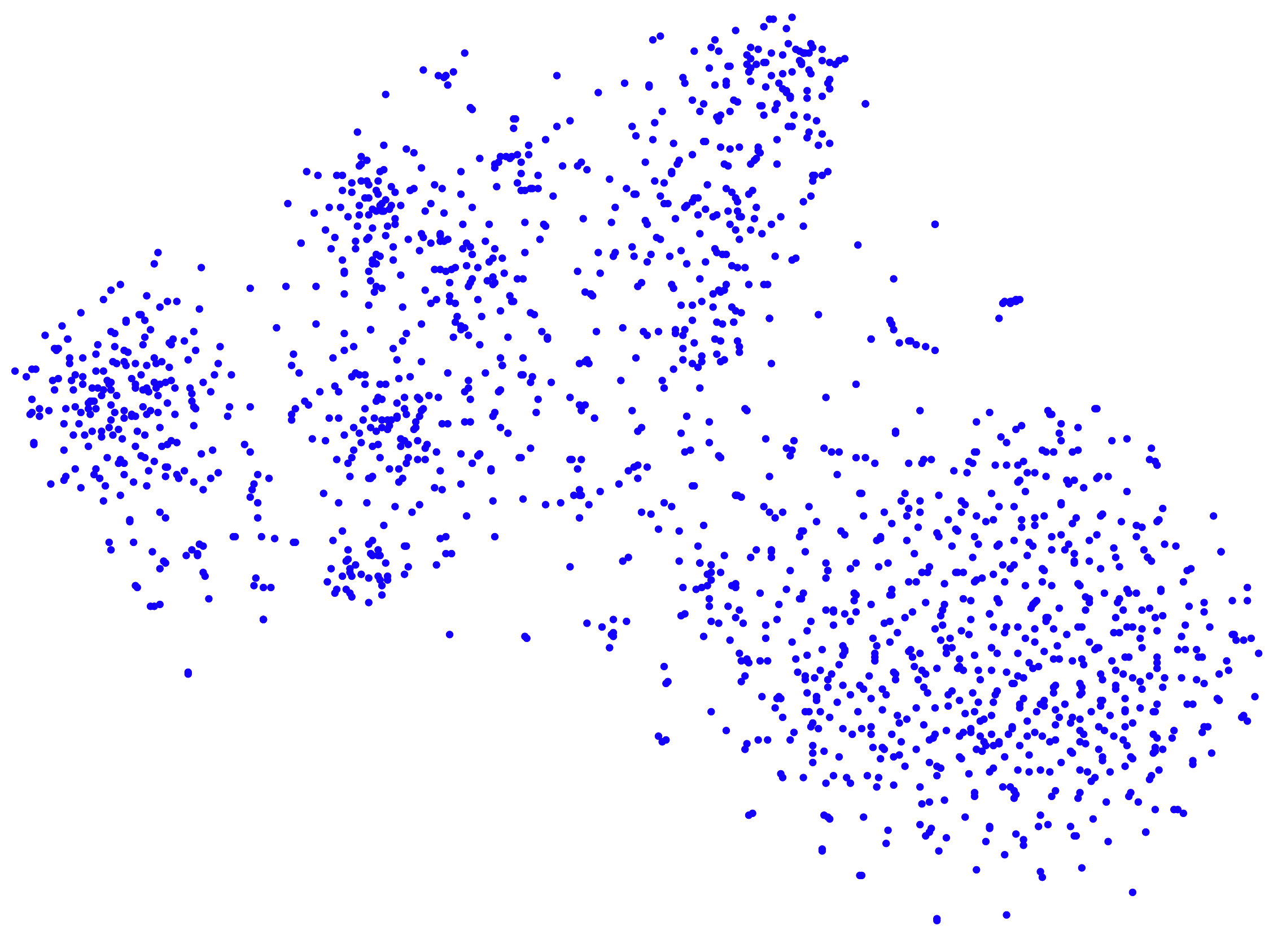

Supplement: Supplementary file 2 — ZIP archive containing VizBin visualization screenshots of the individual bins for the three datasets (37A, 37B, and SRS013705) originally reported in [ 16 ]. [file 40168_2014_66_MOESM2_ESM.zip › 37A_37B_SRS013705/SRS013705/SRS13705.out.031.png]
